# Evolutionary robustness of killer meiotic drives

**DOI:** 10.1002/evl3.255

**Published:** 2021-09-12

**Authors:** Philip G. Madgwick, Jason B. Wolf

**Affiliations:** ^1^ Milner Centre for Evolution, Department of Biology and Biochemistry University of Bath Bath UK

**Keywords:** Gamete killers, gene drive, segregation distorters, selfish genetic elements, suppressors

## Abstract

A meiotic driver is a selfish genetic element that interferes with the process of meiosis to promote its own transmission. The most common mechanism of interference is gamete killing, where the meiotic driver kills gametes that do not contain it. A killer meiotic driver is predicted to spread rapidly through a population at the expense of other genes in the rest of the genome. The rapid spread of a killer meiotic driver is expected to be chased by the rapid spread of a suppressor that returns fair meiosis. Paradoxically, while this might imply that meiotic drivers should be evolutionarily transient, numerous ancient killer meiotic drivers have been discovered that have persisted for millions of years. To understand the rationale that could potentially explain such evolutionary robustness, we explore different possible mechanisms of killer meiotic drive and the different possible associated mechanisms of suppression. We use a framework that considers how the different stages of meiosis result in different structured interactions among cells with different genotypes in various combinations. Across possible interactions, we show that there are three genotypically distinct drive mechanisms that create alternative selective conditions for the spread of different types of suppressors. We show that killer meiotic drivers are more evolutionarily robust if they operate among sister cells (after meiosis I and before meiosis II) than at any other point during meiosis. The different drive mechanisms we identify make testable predictions that could explain why some killer meiotic drivers are transient while others are ancient.

**Impact summary**

Meiotic drivers are a natural form of gene drive that spreads through a population by biasing the process of gamete production to favor their own transmission. Gamete killers, the most common type of meiotic drive, impose a cost on their carriers by producing fewer and/or damaged gametes, which can both directly and indirectly contribute to infertility. Consequently, the theoretical expectation is that killer meiotic drivers give rise to strong counter‐selection that favors suppressing mechanisms that generate resistance to them, suggesting that killer meiotic drivers would appear and rapidly disappear on short evolutionary timescales. But researchers have identified a number of “ancient” killer meiotic drivers that are evolutionarily robust in having persisted for millions of years. The reason why some killer meiotic drivers are more evolutionarily robust than others is almost certainly related to the differences in the mechanisms of gamete killing, but we currently know very little about those mechanisms – and also about what aspects of the mechanisms of gamete‐killing are salient to evolutionary robustness. To address this puzzle, we examine different logical alternatives for how gamete killing could take place and explore their impact on the selection of different types of suppressing mechanisms. We analyze the evolutionary robustness of different gamete killing mechanisms and predict the mechanism that “ancient” killer meiotic drivers are likely to use sister cell killing. Although (as we discuss) current evidence is not able to distinguish whether ancient killer meiotic drivers use this mechanism, we identify plausible molecular pathways to sister killing through heterochromatin disruption and cytoplasmic bridges to pave the way for future experimental research. Additionally, the arising insights of our analysis of the evolutionary robustness of natural gene drives may also aid the design of synthetic gene drives for population control by linking possible molecular mechanisms to their evolutionary consequences.

Meiotic drivers spread through populations by biasing gamete production during meiosis to favor their own transmission (Sandler and Novitski 1957; Zimmering et al. 1970). The most common type of meiotic drive discovered to date uses the gamete‐killing mechanism, where the meiotic driver biases its own transmission by selectively killing more gametes that do not carry the meiotic driver than carry the meiotic driver (Burt and Trivers 2008; Lindholm et al. 2016; Bravo Núñez et al. 2018). Such killer meiotic drivers impose a fertility cost on their carriers by producing fewer and/or damaged gametes (see Zanders and Unckless 2019 for a recent review of mechanisms). Hence, a gamete‐killing meiotic driver is a selfish genetic element that enhances its own transmission at the expense of other genes in the rest of the genome (Leigh 1971; Werren *et al*. 1988; Burt and Trivers 2008). The fitness cost imposed by a meiotic driver generates selection favoring mechanisms that suppress it, either through a resistant allele at the drive locus or a modifier allele at some locus elsewhere in the genome (Leigh 1971; Crow 1991; Hatcher 2000; Price et al. 2019., 2020.). Consequently, while meiotic drivers are predicted to spread rapidly through a population, their fertility costs mean that they are also predicted to be rapidly eliminated through suppression (Crow 1991; Hatcher 2000; Burt and Trivers 2008; Price et al. 2019., 2020.). For this reason, meiotic drivers are thought to appear and disappear on short timescales as transient evolutionary phenomena (Lindholm et al. 2016; McLaughlin and Malik 2017; Price et al. 2019).

Despite the expectation that meiotic drivers should show rapid evolutionary turnover, several relatively ancient meiotic drives have been identified, suggesting that some drive mechanisms are robust to suppression (Hatcher 2000; Lindholm et al. 2016; Price et al. 2019). For example, segregation distorter (SD) in *Drosophila melanogaster* has persisted for over 1 million years (Kovacevic and Schaeffer 2000), the *t*‐haplotype in *Mus musculus* has persisted over 2 million years (Silver 1993), and spore killer (Sk) in *Neurospora intermedia* may have persisted for up to half a million years (Svedberg *et al*. 2018). The problem of the persistence of drive mechanisms is not exclusively about the persistence of polymorphism of drivers and/or modifiers over long timespans (Price et al. 2019., 2020.), but also the persistence of fertility costs with the fixation of drivers (Zanders and Unckless 2019). Meiotic drivers are often discovered after hybridization in a heterozygote (as reviewed in Bravo Núñez et al. 2018), which suggests that these drivers are maintained by selection at fixation to remain robust against the invasion of drive‐suppressing mutations. The evolutionary persistence of meiotic drivers presents a real challenge for current research, both in terms of explaining why some drivers are robust in remaining functional despite their fertility costs, and why other drivers are more transient in succumbing to suppression (Lindholm et al. 2016; Price et al. 2019).

The robustness of killer meiotic drivers to the evolution of suppression likely arises from some specific property of the gamete‐killing mechanism (Price et al. 2019). Although the molecular mechanisms of killer meiotic drivers are still poorly understood, two logical alternatives have been discerned (Lindholm et al. 2016; McLaughlin and Malik 2017; Bravo Núñez et al. 2018), which are supported by different examples (see Bravo Núñez et al. 2018 for a recent review). The first is a “poison‐antidote” drive system, wherein a gene encodes a diffusible poison and its non‐diffusible antidote; the poison diffuses away from cells that produce it but the antidote remains cell‐bound. Consequently, the poison only kills cells that do not produce the antidote. The second is a “killer‐target” drive system, wherein a gene encodes a specific killer element but not its target element and cells with the killer element only kill other cells that have the target element. The central difference between poison‐antidote and killer‐target drive systems is whether gametes are killed based on the absence of the antidote or the presence of the target element, respectively. While this difference could give poison‐antidote and killer‐target drive systems somewhat different evolutionary dynamics (McLaughlin and Malik 2017; Price et al. 2019), this detail of the molecular mechanisms of killer meiotic drive is not sufficient to explain the major differences in the evolutionary dynamics of drive suppressors. For example, ancient examples of both poison‐antidote and killer‐target drive systems have persisted despite the threat of suppressors, including the poison‐antidote *t*‐haplotype in house mice (Silver 1993) and the killer‐target *SD* in fruit‐flies (Kovacevic and Schaeffer 2000). Therefore, the critical details of the molecular mechanisms of killer meiotic drives that explain the differences in evolutionary robustness of the drive systems remain unknown.

To understand the variability in the evolutionary robustness of killer meiotic drives we develop a set of simple models based on different mechanisms of gamete killing. By systematically partitioning mechanisms of killer meiotic drives into different classes, we generate predictions about their relative susceptibility to different modes of suppression. By providing predictions for the properties that explain the variable robustness of killer meiotic drivers, our framework contributes to ongoing empirical research by helping to focus studies on the most likely underlying causes. Further, by providing an understanding of why some natural gene drives are more robust than others, our framework may also provide important insights for the development of effective “synthetic” gene drives (Burt 2014) for population control (Lindholm et al. 2016; Bravo Núñez et al. 2018; Price et al. 2019., 2020.).

## Model and Results

We consider evolution at a killer meiotic drive locus, which we describe in the terms of the poison‐antidote drive system, but the structure of our framework also applies to killer‐target drive systems. We assume that the locus is autosomal, has no effect on sex ratio (because this always favors drive suppression), and its effect is not sex‐limited, although this can be easily incorporated (but does not alter the outcome). This setup follows a classic model of a meiotic drive (Leigh 1971) and so is only briefly described here (while a more detailed description and explanation of assumptions is provided in Appendix A).

The model of meiotic drive assumes a single locus at which a drive allele (D, with frequency $f_{D}$) competes against a nondrive allele (d, with frequency $f_{d}$). The dd homozygote is assigned a fitness of 1. In the DD homozygote, fitness is reduced by a toxicity effect (s, scaled to vary between 0 and 1). In the Dd heterozygotes, the toxicity effect (s) is modulated by the degree of dominance, h (*i.e*. the total toxicity effect is hs; where h can vary between 0 and 1), which can be interpreted as the impact of poison dilution on toxicity. The fact that the toxicity effect is suffered equally by all gametes (regardless of whether they have the drive or nondrive allele) implies that it is the diffusible poison that is costly and the non‐diffusible antidote that is cost‐free. In Dd heterozygotes, the selective killing of gametes with the nondrive allele secures a transmission advantage (+e, where e is scaled to vary between 0 and 1) for the drive allele and a transmission disadvantage (−e) for the nondrive allele. The fitness components arising from the toxicity (s) and transmission effects (e) are multiplicative: the toxicity effect determines the number of viable gametes produced while the transmission effect determines the proportion of those gametes that contains a given allele. In this single‐locus two‐allele scenario, the drive allele can invade whenever:

(1) $$e\left(1-hs\right)-hs>0$$

**Figure 1:**
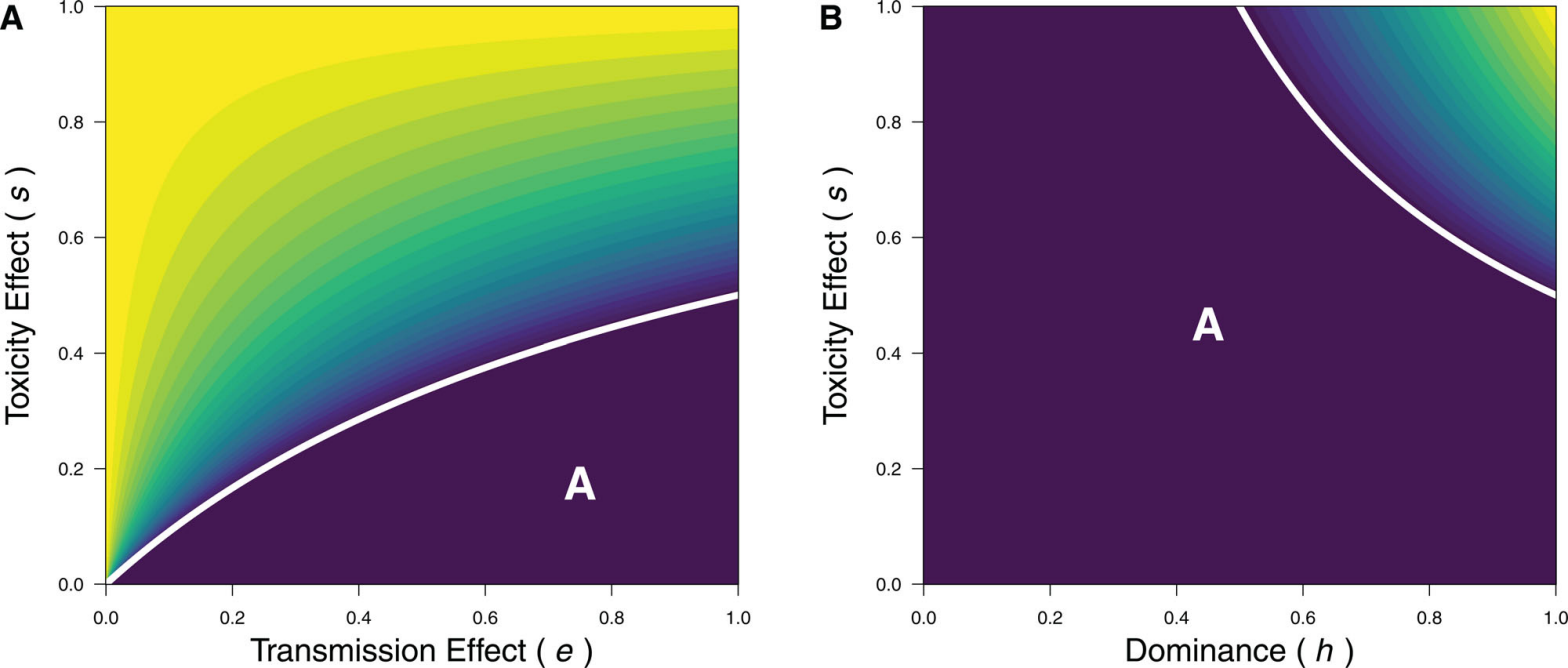
The frequency threshold where a drive allele has higher fitness than a nondrive allele and will invade across parameter space for the single locus scenario (see eqn. 1). Assuming the nondrive allele is at fixation ( $f_{m}=\;1$) for an invasion analysis, the frequency threshold of the drive allele ${f_{D}}^{*}=\frac{1}{s}\;\left(1-(1+e)(1-hs)\right)$ specifies the drive allele frequency where a drive allele has equal fitness to a nondrive allele (derived from eqn. 1). As the drive allele is favored when $f_{D}>{f_{D}}^{*}$, the frequency threshold of the drive allele (${f_{D}}^{*}$) gives the colour scale on the range between ≥1 (yellow) when the drive allele never invades and ≤0 (purple) when the drive allele always invades (labeled “A”; below the limit ${f_{D}}^{*}=\;0$ when s=e/((2+e)h−1); white line). To show the range of selective conditions across parameter variations, two scenarios are given: (**A)** complete dominance of the toxicity effect (h=1) and (**B)** perfect transmission bias (e=1).

When the toxicity effect is dominant (h=1), equation (1) recapitulates a classic result (Leigh 1971) that the drive allele is always able to spread when the product of the fitness components from the toxicity and transmission effects are greater than one (i.e., (1+e)(1−s)>1; see Figure 1). After invasion, a drive allele may either spread to fixation or reach a stable polymorphic equilibrium:

(2) $$\hat{f_{D}}=\frac{e\left(1-hs\right)-hs}{s\left(1-2h\right)}\;$$

When the drive allele can invade, this polymorphic equilibrium is only possible when there is a large but recessive toxicity effect (see Appendix A for further details), which follows other models of meiotic drive (e.g., Lewontin and Dunn 1960; Curtsinger and Feldman 1980; Holman *et al*. 2015). These results provide a key baseline for understanding the conditions that favor a drive allele in more complex scenarios.

### INTRALOCUS SUPPRESSION BY A RESISTANT ALLELE

We can suppose that the drive locus could experience three qualitatively different kinds of mutation. First, a mutant could be incapable of producing both the poison and the antidote, which would render it equivalent to the nondrive allele. Second, a mutant could produce the poison but not the antidote, which would be nonviable because it would poison itself. Third, a mutant could produce the antidote but not the poison. This mutation would generate a viable resistant allele because the drive allele would not reap a transmission advantage (+e) against it and it would not suffer the toxicity effect (s) when homozygous. Assuming there is no cost to resistance (see Appendix A for a discussion of the impact of costly resistance), the fitness of the resistant allele is always greater than that of the nondrive allele because it does not suffer a transmission disadvantage. Whether or not there is a cost to resistance (from antidote production, which would be paid by both drive and resistant alleles), the fitness of the resistant allele is also always greater than the fitness of the drive allele in the absence of the nondrive allele because it suffers a dominance‐reduced toxicity cost (see Table S1; given that h ranges between 0 and 1). Hence, the drive allele is expected to spread through a population of nondrive alleles but, if a resistant allele arises by mutation, it would also spread, leading to intralocus suppression of the drive allele. Therefore, as is well‐known (e.g., Prout *et al*. 1973; Crow 1991; Scott and West 2019; see also Price *et al*. 2020), a drive allele is always susceptible to intralocus suppression by a resistant allele.

### INTERLOCUS SUPPRESSION BY MODIFIERS

A meiotic driver could also be suppressed by a modifier elsewhere in the genome. Such interlocus suppression could be achieved through various pathways and so we make several simplifying assumptions (which are further described and explained in Appendix A). We assume the modifier is cost‐free (like a resistant allele), can only modify the drive effect when in the same cell as the drive allele, and can only completely block poison and/or antidote production. With these restrictions, we can suppose two viable types of interlocus modifiers. First, a modifier could fully suppress the drive allele, making it incapable of producing the poison and the antidote. Second, a modifier could partially suppress the drive allele by blocking poison production but not antidote production. The third scenario of a modifier that partially suppresses the drive allele to produce the poison but not the antidote can be ignored since (as above) it would be nonviable owing to self‐poisoning.

**Figure 2:**
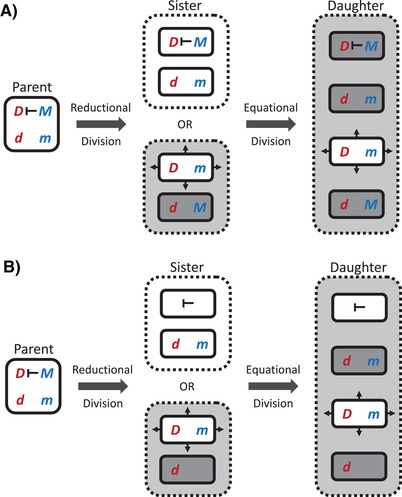
A simplified diagram showing the potential for different types of gamete‐killing gene drive throughout the steps of the meiotic divisions in a double heterozygote (patterns in other genotypes are illustrated in Figure S1). During meiosis, a parental cell undergoes a first reductional division into two sister cells followed by a second (mitosis‐like) equational division into four daughter cells. Different cells are presented with solid outlines, and different interactions between cells are presented with dashed outlines (except for the possible interaction between the parent cell and the sister cells it produced). Alleles at the drive locus are presented in red (D = drive and d = nondrive) and alleles at the modifier locus are presented in blue (M = modifier and m = nonmodifier). A drive allele for a poison‐antidote pair can distort its transmission to the next generation by producing a poison (indicated by out‐facing arrows from the producer cell) that leads to the presence of the poison within that given interaction (indicated by the light‐grey shading in dashed outlined box). The presence of the poison kills gametes that do not also produce the antidote with cell death indicated by the dark‐grey shading in solid outlined boxes). The modifier allele (M) only has an effect on the drive (D) allele if it is within the same cell, which is indicated by the turnstile symbol ⊢ that indicates that the drive allele has blocked some function of the drive allele (with different scenarios illustrated in A and B). As such, in the double heterozygote scenario presented here, there is no potential for pre‐meiotic killing, where the parent cell produces the poison that kills sister or daughter cells, because the modifier blocks poison production. However, there is potential for meso‐meiotic killing between sister cells (of which there are two potential genotypic combinations represented by different interactions in dashed boxes) and post‐meiotic killing between daughter cells (ignoring the potential for daughter cells to kill sister cells in this simplified diagram). (**A)** The case of a fully suppressing modifier that blocks poison and antidote production by the drive allele, rendering it susceptible to the poison produced by other cells. (**B)** The scenario of a partially suppressing modifier blocks that poison production only, and so remains resistant to the poison produced by other cells.

Because such modifiers have functional constraints on their consequences, the mechanism of meiotic drive can impact the evolution of interlocus suppression. Following‐on from other analyses of the structured interactions among different cell types during meiosis (see: Haig and Grafen 1991; Haig 1993, 2010.; Hurst and Randerson 2000), we can classify potential drive mechanisms based on the separation of the cell type that produces the poison and the cell type when/where the poison takes effect (Table 1). The critical detail that distinguishes cell types is not the number of alleles per cell, but the combination of alleles in and across interacting cells. This combination can be altered by recombination, crossing‐over, and linkage disequilibrium, but the extensive analysis in Appendix B demonstrates that these phenomena do not alter the basic results that are outlined here. In their absence, meiosis takes place when a parent cell undergoes reductive division (*sensu* cytology) that separates homologous chromosomes into two sister cells. Because these sister cells contain two copies of the same chromosome, they are homozygous at all loci. These cells then undergo equational division (*sensu* cytology) that separates sister chromatids to form four daughter cells that have one allele per locus. Across all possible combinations of interactions between cell types, there are three viable types of meiotic killers with genotypically distinct interaction structures. First, there is “pre‐meiotic killing” where poison is produced in a parental cell (or some other somatic gonadal cells) before meiosis, but the antidote that prevents killing is produced in the sister or daughter cells. Second, there is “meso‐meiotic killing” where poison and antidote are produced in the sister cells after the first reductional division of meiosis I and before the second equational division of meiosis II. Third, there is “post‐meiotic killing” for all other combinations where the poison and/or antidote is produced in the sister and/or daughter cell types. The difference between meso‐ and post‐meiotic killing is in the interaction structure; the products of independent assortment during meiosis I produce two genotypically distinct sister cell interactions as isolated dyads, whereas all four possible daughter cells produced by meiosis II interact as a tetrad (see Figure 2 for an illustration using the example of the double heterozygote). There is also a non‐viable type of meiotic killer (that we therefore ignore) where poison is produced in and takes effect on parent (or somatic) cells that contain all alleles, which does not produce a transmission effect.

**Table 1:** The different combinations of cell types that are poison producers and targets of the poison during meiosis. A producer describes what cell type expresses the poison and the target describes in what cell type poison takes effect (if a cell does not produce the antidote). The steps of meiosis have three different cell types: parent cells undergo reductional division in meiosis I to form two sister cells that undergo a further equational division in meiosis II to form four daughter cells. The sister and daughter cell types are distinguished based on the groupings of cell genotypes, where with the products of independent assortment during meiosis I leading to two genotypically‐distinct sister cell interactions as isolated dyads rather than all the products of meiosis II mixing as one interaction among the four possible daughters cell genotypes as a tetrad

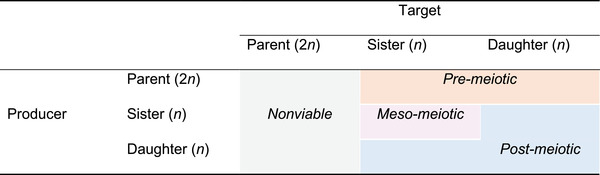

### SUPPRESSION OF A PRE‐MEIOTIC KILLER

We first evaluate the case with interlocus suppression of pre‐meiotic killing, where the drive allele is expressed in parent (or somatic) cells. Modifiers causing full or partial suppression have the same effect in this scenario because diploidy means that the modifier is always in the same cell as any potential drive alleles. Consequently, since the modifier blocks poison production, the drive allele is only active in the absence of the modifier, giving the modifier a “dominant” phenotype (with one allelic copy performing the same role as two copies). We model an interlocus modifier as a system with two unlinked loci: a modifier locus with the modifier allele M (with frequency $f_{M}$) and nonmodifier allele m (with frequency $f_{m}$), and a drive locus (as above) with the drive allele D and nondrive allele d. The fitness model for this scenario is in Table S2, which derives from the gamete fitness values in Table S3. The change in drive allele frequency is:

(3a) $$\Delta f_{D}=f_{D}\;f_{d}f_{m}^{2}[e\left(1-hs\right)-s\left(f_{D}+h\left(f_{d}-f_{D}\right)\right]/\bar{w}$$

which matches the single locus case (see eqn. S1 in Appendix A), except that selection on the drive allele depends on the frequency of the modifier allele, such that the value of equation (3a) goes to zero as the modifier allele goes to fixation (*i.e*., the modifier allele removes selection favoring the drive allele). The change in the modifier allele frequency is:

(3b) $$\Delta f_{M}=f_{D}\;f_{M}f_{m}^{2}s\left[f_{D}+2hf_{d}\right]/\bar{w}$$

Therefore, the modifier allele is always favored because all terms in equation (3b) are non‐negative, with the magnitude of its advantage depending on the frequency of the drive allele ($f_{D}$). When the modifier allele is at fixation the drive allele is selectively neutral. Accordingly, a drive allele for pre‐meiotic killing is always susceptible to an interlocus modifier for full and/or partial suppression.

### SUPPRESSION OF A POST‐MEIOTIC KILLER

In post‐meiotic killing, the drive allele is expressed in the daughter cells (i.e., gametes) after meiosis. In this scenario, a fully supressing modifier that blocks both poison and antidote production has no control over poison and antidote production in gametes that lack the modifier. Consequently, in double heterozygotes (DdMm), the modifier allele blocks expression of the poison and antidote in 25% of the gametes, while 25% still produce the poison and antidote (see Figure 2). Therefore, the modifier has a ‘recessive’ phenotype, as only modifier allele homozygotes block poison production in all gametes. This scenario leads to a symmetry between the fitness of the drive (D) and nonmodifier (m) alleles, and between the nondrive (d) and modifier (M) alleles. The modifier and nondrive alleles have similar fitness because they have the same phenotype (see Table S2 for the fitness of alleles and Table S4A for the fitness of gametes), which means the modifier avoids the toxicity effect but is susceptible to drive. The change in the frequency of the drive and modifier alleles are:

(4a) $$\begin{array}{ccc} \Delta f_{D} & = & f_{D}\;f_{d}f_{m}\left[e\left(1+f_{M}\right)\left(1-hs\right)\right.\\ & & \left.-\;s\left(f_{D}\left(f_{m}-2h\right)+h\left(1+f_{M}\right)\right)\right]/\bar{w} \end{array}$$

(4b) $$\begin{array}{ccc} \Delta f_{M} & = & f_{D}\;f_{M}f_{m}\left[-e\left(1+f_{d}\right)\left(1-hs\right)\right.\\ & & \left.+\;s\left(f_{D}\left(f_{m}-h\right)+2hf_{M}\right)\right]/\bar{w} \end{array}$$

Concurrently, the drive allele can invade when:

(5a) $$\left(1-f_{M}^{2}\right)\left(e\left(1-hs\right)-hs\right)>0$$

Hence, the drive allele may only invade when the nonmodifier allele is present, but otherwise equation (5a) recapitulates the condition for the drive allele to be favored in a one‐locus system (eqn. 1). The modifier can invade when:

(5b) $$f_{D}\left[-e\left(1+f_{d}\right)\left(1-hs\right)+f_{D}s\left(1-h\right)\right]>0$$

The conditions favouring invasion by the modifier allele are approximately (but not exactly) opposite to those that favor the drive allele, with a larger toxicity effect (s) and smaller transmission effect (e) favoring modifier invasion. However, the sign of the invasion criterion for the modifier allele directly depends on the frequency of the drive allele, such that higher drive allele frequencies favor modifier allele invasion. In the absence of the modifier allele, if the drive allele can invade then it will either spread to fixation or to a stable equilibrium (following the conditions in eqn. 2). To evaluate when a modifier allele can invade, we used a numerical analysis, which confirms that when the drive allele can invade and is at fixation or its polymorphic equilibrium, the modifier allele can never invade. Additionally, were the modifier allele to invade, the only stable equilibrium is the loss of the modifier allele. Therefore, with post‐meiotic killing, a modifier allele for full suppression is never favored by selection.

We next consider partial suppression by an interlocus modifier that blocks poison but not antidote production. In this scenario (see Table S4B) there is no change to the fitness of alleles at the drive locus, so the changes in allele frequencies are the same as with a fully suppressing modifier (eqn. 4a). However, the fitness of alleles at the modifier locus is changed because a fully suppressing modifier allele suffers the transmission disadvantage while a partially suppressing modifier does not. The change in the frequency of the modifier under the partial suppression scenario is:

(6) $$\Delta f_{M}=f_{D}\;f_{M}f_{m}s\left[f_{D}\left(f_{m}-h\right)+2hf_{M}\right]/\bar{w}$$

Consequently, the modifier allele can invade when $f_{D}^{2}s\left(1-h\right)>0$, which is always met when the drive allele is present ($f_{D}$ > 0) and there is a toxicity effect (s>0) and/or the toxicity effect is not completely dominance (h<1). Complete dominance of the toxicity effect does not prevent modifier allele invasion but would give it have the same phenotype as the nonmodifier allele (i.e., rendering it neutral). The modifier allele has no stable equilibria wherein the drive allele is also at a stable equilibrium. This should result in the loss of the drive allele, whereupon the modifier allele is a neutral variant; the subsequent persistence of the modifier allele could prevent the future invasion of a drive allele. Therefore, while a drive allele for post‐meiotic killing is not susceptible to the invasion of a fully suppressing modifier, it is susceptible to a partially suppressing modifier.

### SUPPRESSION OF A MESO‐MEIOTIC KILLER

In meso‐meiotic killing, the drive allele is expressed in sister cells after the first reductional division of meiosis I and before the second equational division of meiosis II. Following the post‐meiotic killing scenario, a fully suppressing modifier only affects expression of the poison and antidote within the gametes that carry the modifier. The meso‐meiotic and post‐meiotic killing scenarios only differ in the pattern of allelic fitness in the double heterozygotes (DdMm) because post‐meiotic killing takes place between all four daughter cells (which have all possible haploid genotypes), while meso‐meiotic killing takes place between sister cells (which always have opposite genotypes; see Figure 2). For all other genotypes, the two sister cells have the same genotype as the four daughter cells, but in double heterozygotes the poison production only occurs in one sister cell pair (Dm×dM; not DM×dm), limiting which gametes suffer the toxicity effect (s) and transmission effect (e). The fitness of alleles is provided in Table S2 and the fitness of gametes in Table S5A. The change in drive and modifier allele frequencies are:

(7a) $$\Delta f_{D}=f_{D}\;f_{d}f_{m}\left[e\left(1-hs\right)-\;s\left(f_{D}f_{m}\left(1-2h\right)+h\right)\right]/\bar{w}$$

(7b) $$\Delta f_{M}=f_{D}\;f_{M}f_{m}\left[-e\left(1-hs\right)+s\left(f_{D}f_{m}\left(1-2h\right)+h\right)\right]/\bar{w}$$

The conditions favoring the drive allele are equal and opposite to those favoring the modifier allele. Consequently, the drive allele can invade (and hence the modifier cannot invade) when:

(8a) $$f_{m}\left[e\left(1-hs\right)-hs\right]>0$$

Therefore, the drive allele may only invade when the nonmodifier allele is present, but otherwise equation (8a) recapitulates the condition for the drive allele to be favored in a one‐locus system (eqn. 1). The modifier can invade when:

(8b) $$f_{D}\left[s\left(f_{D}\left(1-2h\right)+h\right)-e\left(1-hs\right)\right]>0$$

which is a trivial result in that the modifier can only invade when the drive allele is present, but the conditions that allow the drive allele to invade would prevent the modifier from invading. While this result is not immediately obvious from equation (8b), it can be confirmed by numerical simulation. Therefore, a drive allele for meso‐meiotic killing is not susceptible to a fully supressing modifier.

For a partially suppressing modifier, the fitness model is very similar to full suppression except that, when the drive allele is homozygous and the modifier allele is heterozygous (DDMm), there is no drive despite poison production (by the Dm sister cell) because both sister genotypes produce the antidote. Consequently, the fitness of the alleles at the drive locus follows the full suppression scenario (see Table S2 and Table S5B for the fitness of gametes). Therefore, the drive allele is favored and can invade under the same conditions (see eqns. 7a and 8a). However, the fitness of the alleles at the modifier locus differs under this scenario. Consequently, change in the frequency of a modifier allele is:

(9) $$\Delta f_{M}=f_{D}\;f_{M}f_{m}\left[-ef_{d}\left(1-hs\right)+s\left(f_{D}f_{m}\left(1-2h\right)+h\right)\right]/\bar{w}$$

And the invasion criterion for a partially supressing modifier is:

(10) $$f_{D}\left[s\left(f_{D}\left(1-2h\right)+h\right)-ef_{d}\left(1-hs\right)\right]>0$$

which is nearly identical to equation (8b) except for the presence of the $f_{d}$ term that relaxes the invasion conditions such that a partially suppressing modifier is increasingly favored as the drive allele frequency ($f_{D}$) increases. This is due to the fact that the modifier allele's invasion depends on its fitness when in the DdMm double heterozygote (see Table S5B), where the modifier allele (M) experiences a transmission disadvantage when in a sister cell with a nondrive allele (d), even though it blocks poison production to restore fair meiosis when it is with a drive allele (D). The modifier allele can always invade when the drive allele is at fixation or its polymorphic equilibrium (eqn. 2), but it cannot always invade when the drive allele is at a non‐equilibrium frequency, which can be confirmed by numerical simulation. When the modifier allele can invade there are no stable equilibria at which the drive allele is also at a stable equilibrium. This should lead to loss of the drive allele, which renders the modifier allele neutral. The subsequent persistence of the modifier allele could prevent future invasion by drive alleles. Therefore, a meso‐meiotic killer can resist the spread of fully suppressing modifier and also, conditionally, a partially suppressing modifier.

## Discussion

To understand the evolutionary robustness of killer meiotic drive to the threat of suppression, we systematically considered different mechanisms of killer meiotic drive through the combinations of potential interactions among cells during meiosis. The phases of meiosis alter the arrangements of cells among combinations of genotypes, which dictates the opportunity for the drive mechanism to promote itself and potentially be suppressed by a modifier. Our analysis indicates that there are three plausible types of meiotic killers: a pre‐meiotic killer is expressed in the parent cell and takes effect in either sister or daughter cell types, a meso‐meiotic killer is expressed in and takes effect in the sister cell types, and a post‐meiotic killer is expressed in and takes effect in the other combinations of sister and daughter cell types. These different types of killer systems are potentially open to suppression through mechanisms that either block both poison and antidote production by the killer drive allele (‘full suppression’) or only block poison production (“partial suppression”). Both types of suppression could arise from a mutation at the drive locus or from a modifier allele elsewhere in the genome. Because of the different patterns of interactions between cells with different genotypes, the robustness of a killer meiotic drive system to different types of suppression (full vs. partial, intralocus vs. interlocus) will depend critically on which phase of meiosis the drive mechanism takes place.

Through the systematic consideration of combinations of mutations to the poison and antidote components of the drive system, we show that all three types of killer meiotic drive are equally threatened by a resistant allele arising at the drive locus that produces the antidote but not the poison. However, the different types of killer meiotic drives differ in their susceptibility to interlocus modifiers causing full or partial suppression. We find that pre‐meiotic killers are the least evolutionarily robust because they are susceptible to suppression by a full or partial suppressor at a modifier locus, while post‐meiotic killers are more robust because they are only susceptible to a partial suppressor. In contrast, meso‐meiotic killers are the most evolutionarily robust because they are invulnerable to a full suppressor and are conditionally invulnerable to a partial suppressor. Meso‐meiotic killers are always susceptible to partial suppression when the drive locus is in equilibrium but can be robust under nonequilibrium conditions because a modifier allele causing partial suppression can suffer a transmission disadvantage to a nonmodifier allele in double heterozygotes (Table S5B). Therefore, our analysis provides the general prediction that the killer meiotic drivers that evade suppression to persist over long stretches of evolutionary time are most likely to be meso‐meiotic killers (see Table 2).

**Table 2:** A summary of the main results showing the susceptibility of three types of meiotic killer to three types of suppressor. Given that the drive allele is able to invade the nondrive allele, the suppressing modifier allele is “Always” (red) or “Never” (blue) or “Conditionally” (purple) favored by natural selection against the nonmodifier allele

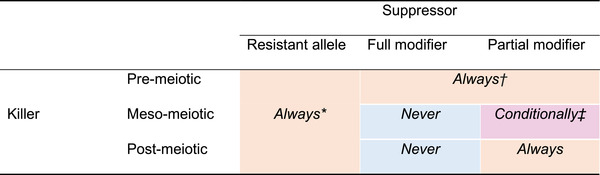

* All meiotic killers have the same evolutionary robustness to a resistant allele (see eqn. S4 Appendix A).

^†^ A fully and partially suppressing modifier are evolutionarily equivalent under pre‐meiotic killing.

^‡^ A modifier is more likely to be favoured when the drive allele is nearer fixation (see eqn. 9).

An important caveat to the general prediction is that our analysis has assumed that any type of suppressor is cost‐free, which provides the most generous conditions for the invasion and spread of suppressors. We assumed that the fitness cost of meiotic drive is from poison production, but suppression would be costly if there is a fitness cost from antidote production. This would favor the nondrive and fully suppressing modifier alleles that do not produce the antidote but would otherwise have no effect on the selection of drive, resistant, or partially suppressing modifier alleles (see Appendix A). If a suppressor has an additional cost, other studies have shown that it can be disfavored (Scott and West 2019; see also: Prout *et al*. 1973; Crow 1991; Burt and Trivers 2008; Price *et al*. 2020), which could help explain the evolutionary persistence of ancient meiotic drives (especially against resistant alleles). This can complement our analysis, which shows that modifiers can be disfavoured because of the phase of meiosis where drive occurs; yet, without an explanation of why such additional costs arise in some circumstances but not in others, it does not explain why some meiotic drives are ancient whilst others are transient.

Meso‐meiotic killers are biologically plausible within known mechanisms of meiotic drive, but they may be challenging to identify. Meiosis has general mechanisms that impede meiotic drive in recombination and crossing‐over (that are included in our analysis; see Appendix B), but there are specific opportunities to overcome these impediments (Leigh 1971; Crow 1991; Haig and Grafen 1991; Hurst and Pomiankowski 1991.; Haig 1993, 2010.; Hurst and Randerson 2000). Consequently, the identification of a meso‐meiotic killer requires detailed knowledge of when/where the drive mechanism is expressed and takes effect. Many ancient meiotic drives have been widely assumed to be post‐meiotic killers (Burt and Trivers 2008; Bravo Núñez et al. 2018) but could plausibly be meso‐meiotic killers because the currently available experimental evidence is often unable to distinguish between the two. One system where the critical data have been gathered is the Paris‐X^SR^ chromosome, which is not a meso‐meiotic killer because the killing protein is expressed, binds, and disrupts the Y‐chromosome heterochromatin as a pre‐meiotic killer, even though gamete destruction takes place in anaphase II through the mis‐segregation of Y‐chromatids (Cazemajor *et al*. 2000; Helleu *et al*. 2016). Nonetheless, this system points to a possible molecular mechanism for meso‐meiotic killing through heterochromatin disruption during meiosis I. An alternative mechanism of meso‐meiotic killing is also possible through the retention or distribution of poisons/antidotes via cytoplasmic bridges among sperm cells such as for the *t*‐haplotype (Zheng *et al*. 2001), which critically depends on the genetic identity of bridged cells; but, the *t*‐haplotype is a post‐meiotic killer because the bridges extend among cells that have had separate independent assortment events.

Beyond explaining the evolutionary robustness of natural meiotic drives, our results have the potential for application in the design of synthetic gene drives for population suppression (*cf*. Champer *et al*. 2020 ), suggesting that, where possible, the use of meso‐meiotic killers would provide the most robust mechanism. While gene drives that are invulnerable to suppression may be unsafe because they would produce irreversible effects, meso‐meiotic killers are still vulnerable to a resistant allele at the drive locus (assuming that it has a sufficiently low cost of resistance; Scott and West 2019), which could provide a means of controlling their release. Moreover, outside of direct application, our results suggest that the potential threat of suppression could be better understood by considering the molecular details of the drive mechanism, like the structure of interactions that bring about meiotic drive (see also: Haig and Grafen 1991; Haig 1993, 2010.; Hurst and Randerson 2000).

## Supporting information

Figure S1. Simplified diagrams of different types of gamete‐killing gene drive throughout the steps of the meiotic divisions across all genotypic combinations.

Table S1. Allelic fitness of drive, nondrive and resistant alleles in a single locus system.
Table S2. Allelic fitness across pre‐, post‐ and meso‐meiotic killer scenarios in the interlocus systems.
Table S3. Fitness of gametes produced by each of the two‐locus genotypes under pre‐meiotic killing.
Table S4. Fitness of gametes produced by each of the two‐locus genotypes under post‐meiotic killing.
Appendix A. Additional model description, explanation and analysis.
Appendix B. Analysis of impact of linkage disequilibrium, recombination, and crossovers.
