# Supplementary figures and images for: Evolutionary robustness of killer meiotic drives
Source: Evol Lett. 2021 Sep 12;5(5):541–50. doi: 10.1002/evl3.255 (PMC8484726; doi:10.1002/evl3.255)

| A)   | $mm$                                                                                | $Mm$                                                                                 | $MM$                                                                                  |
|------|-------------------------------------------------------------------------------------|--------------------------------------------------------------------------------------|---------------------------------------------------------------------------------------|
| $DD$ | 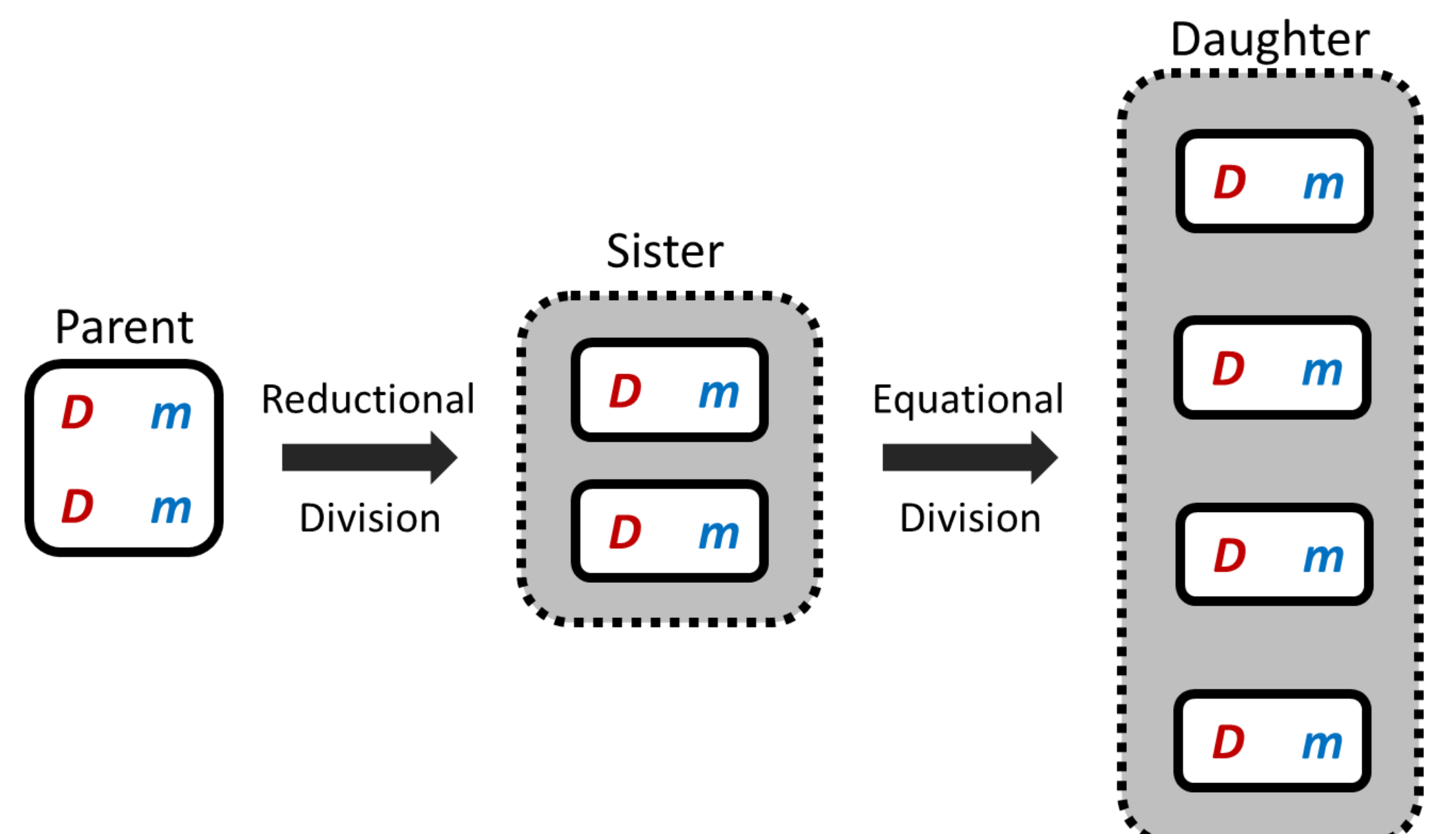   | 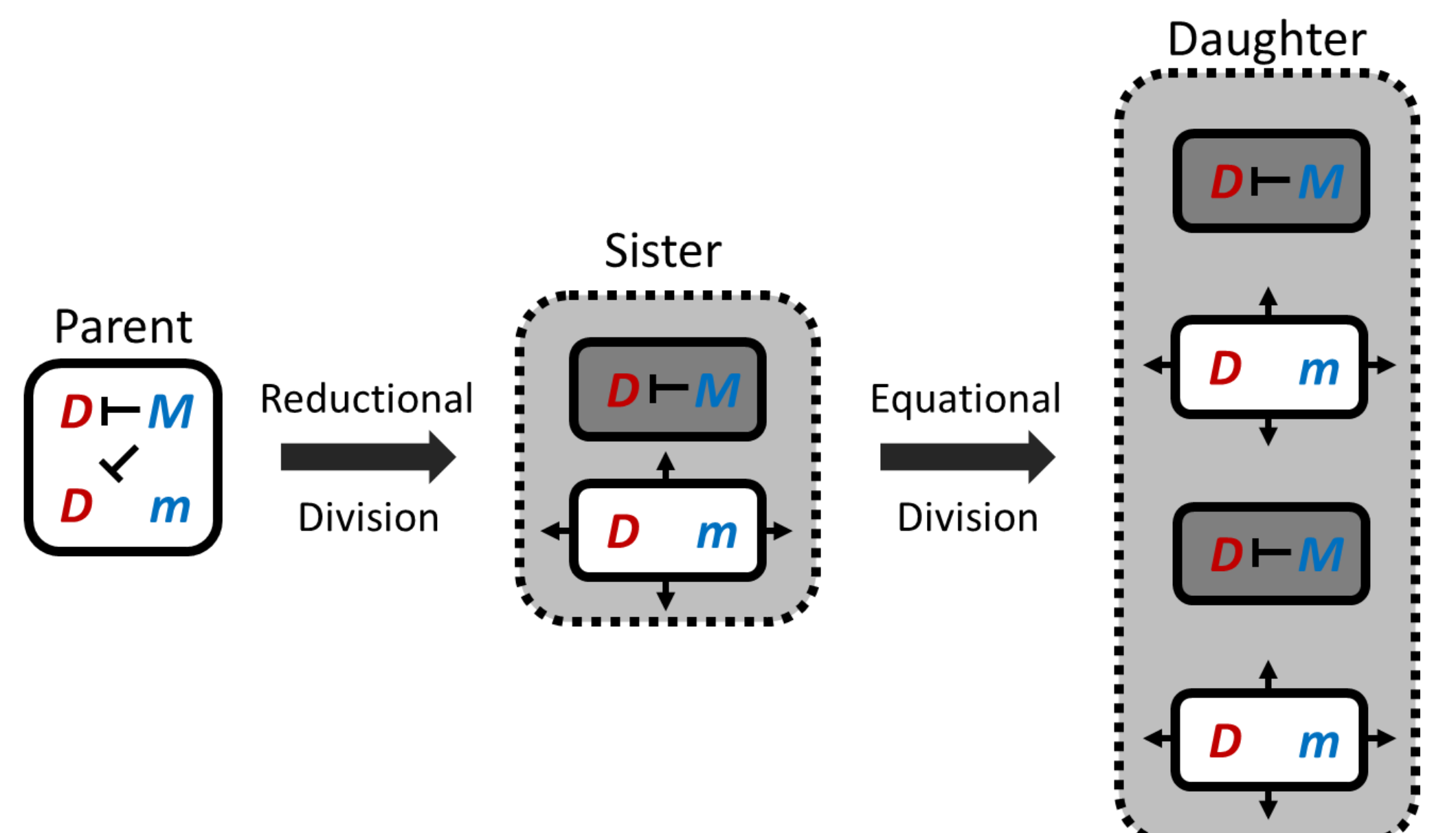   | 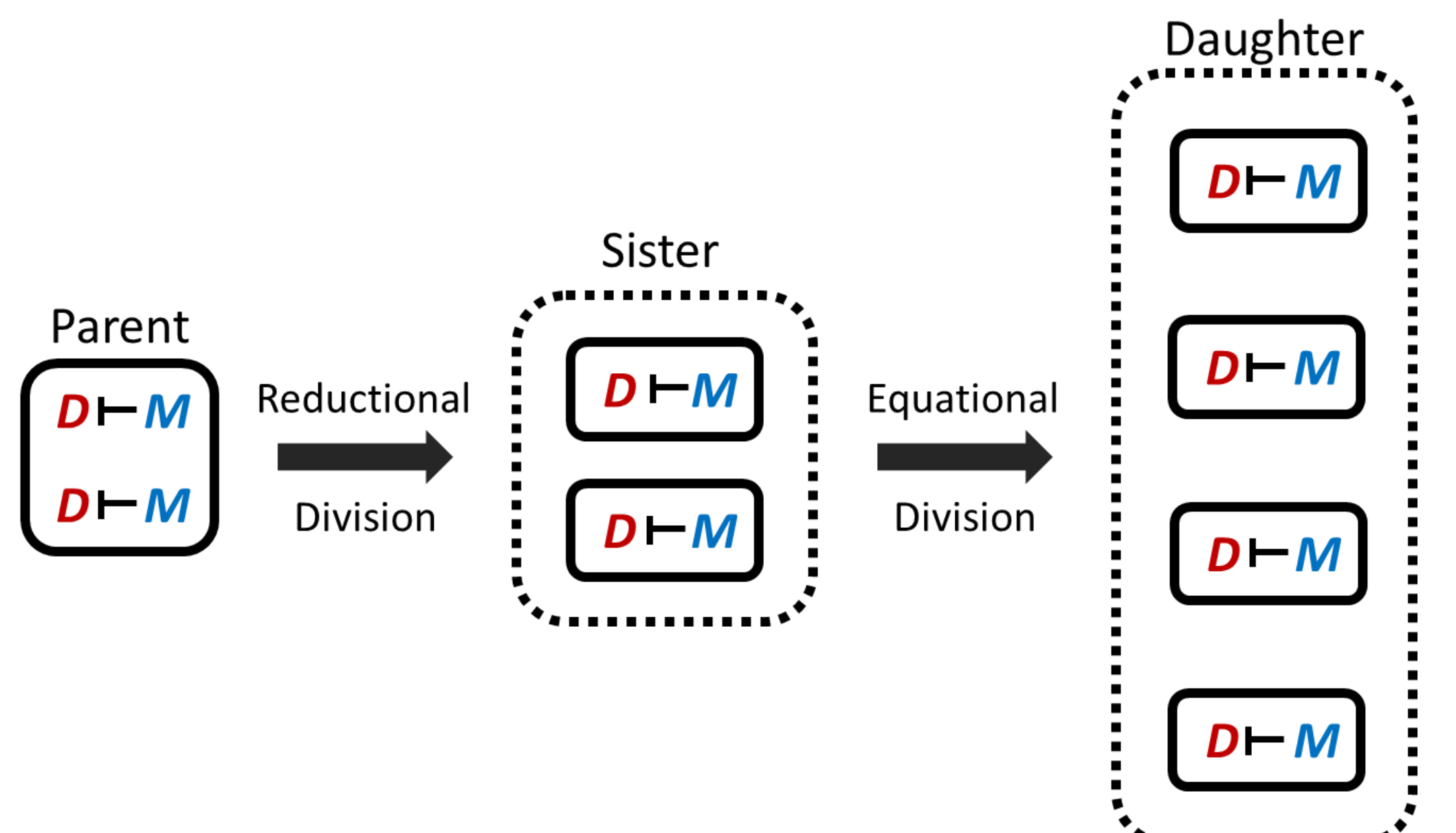   |
| $Dd$ | 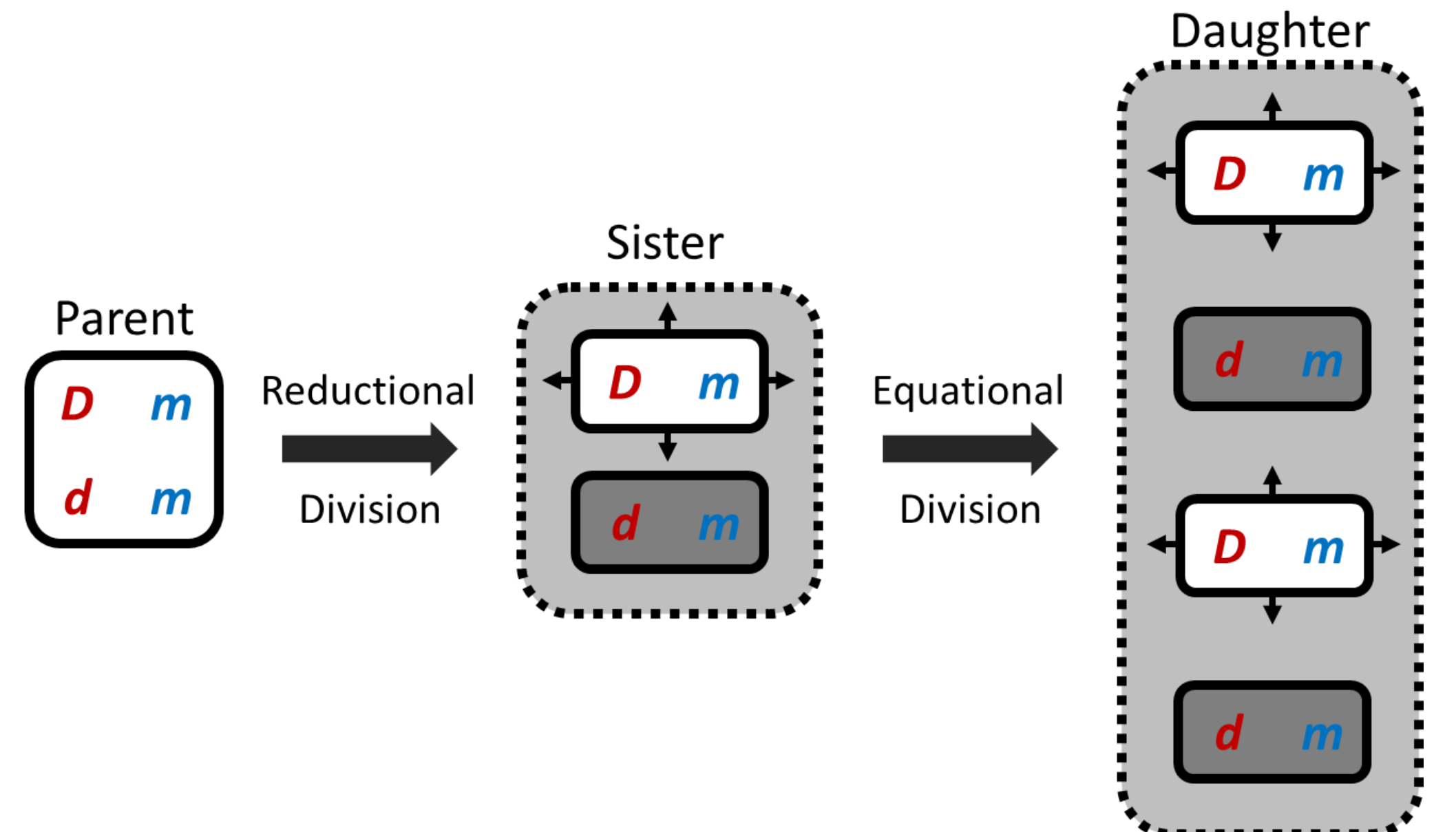 | 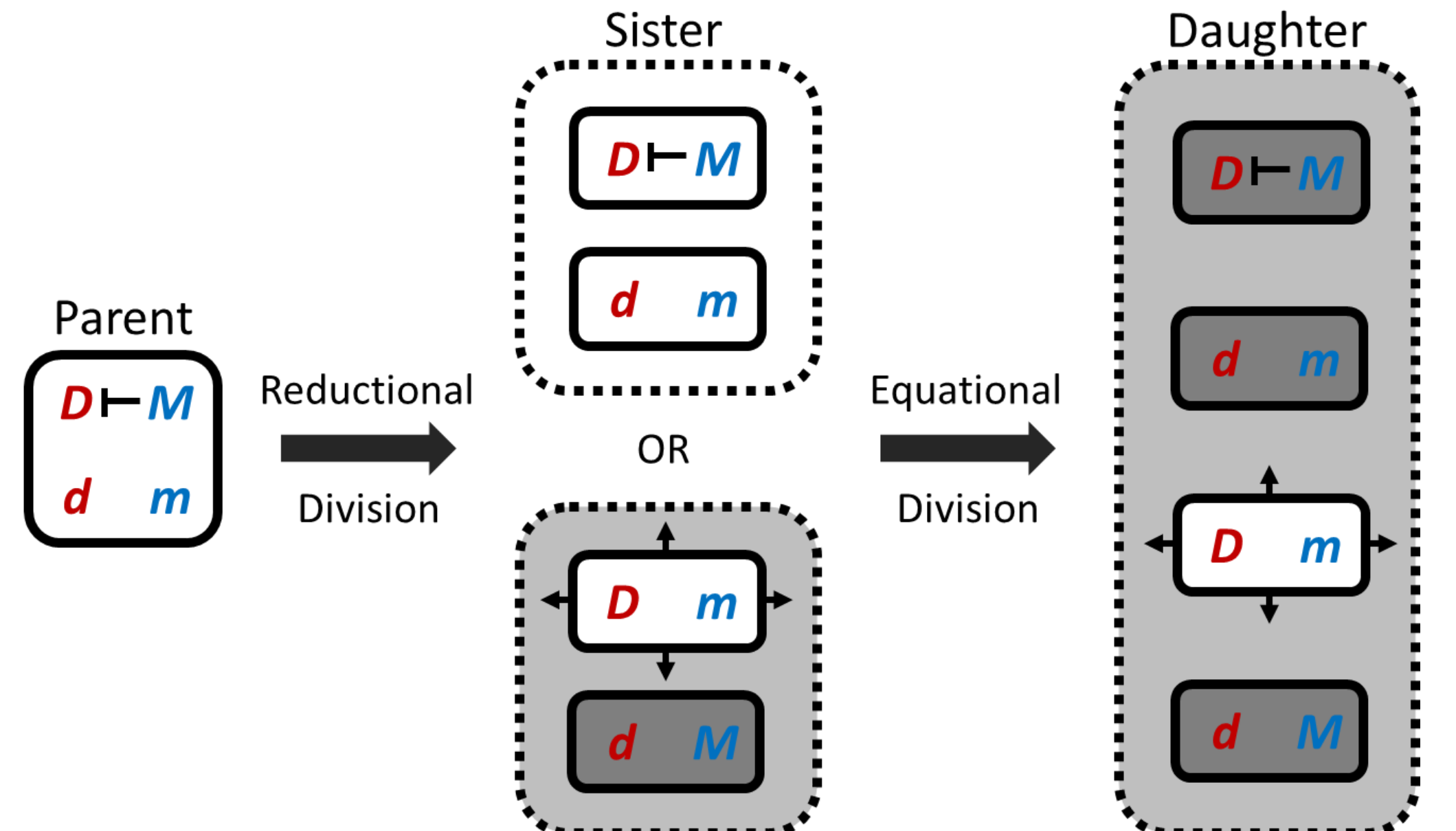 | 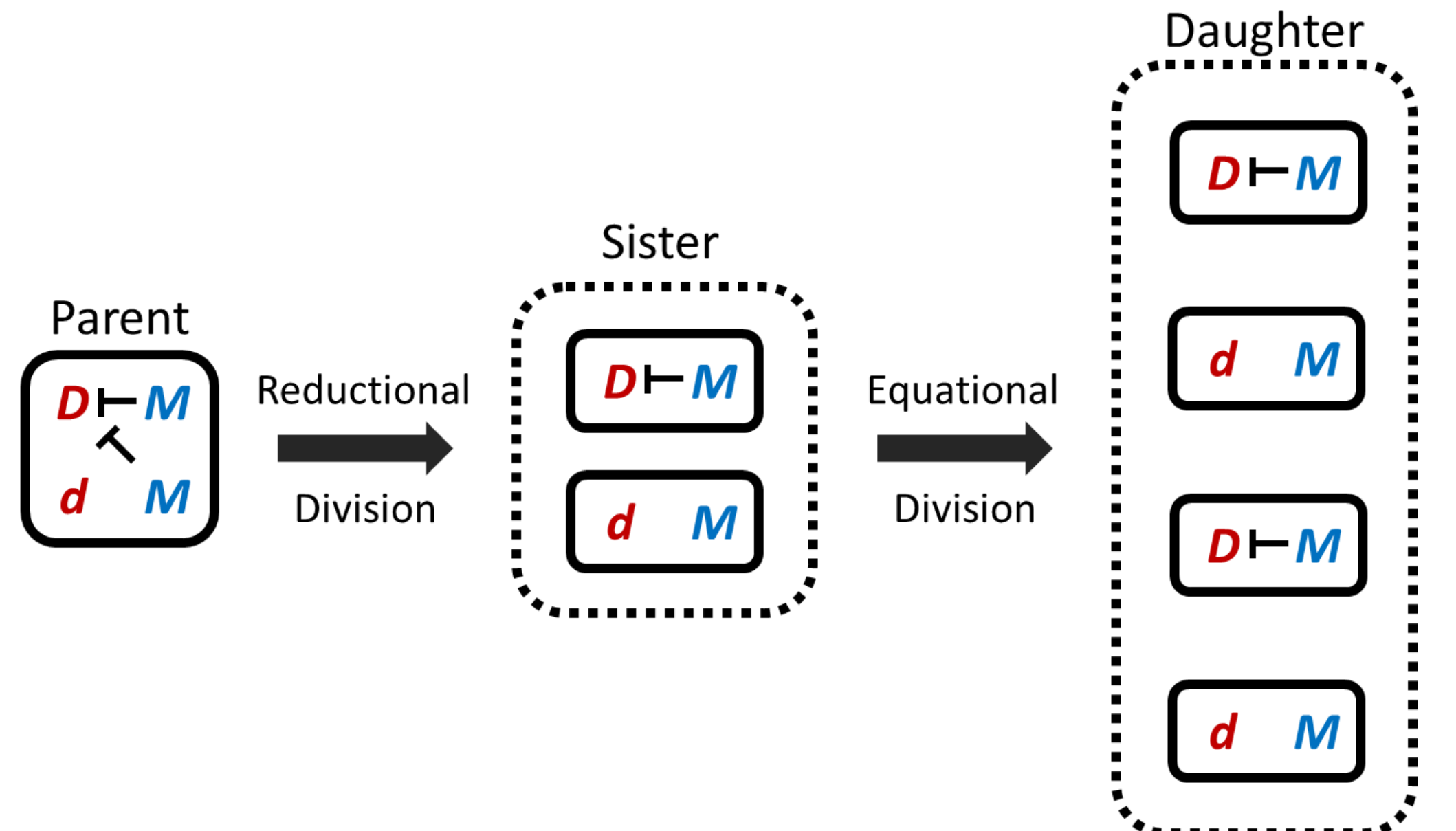 |
| $dd$ | 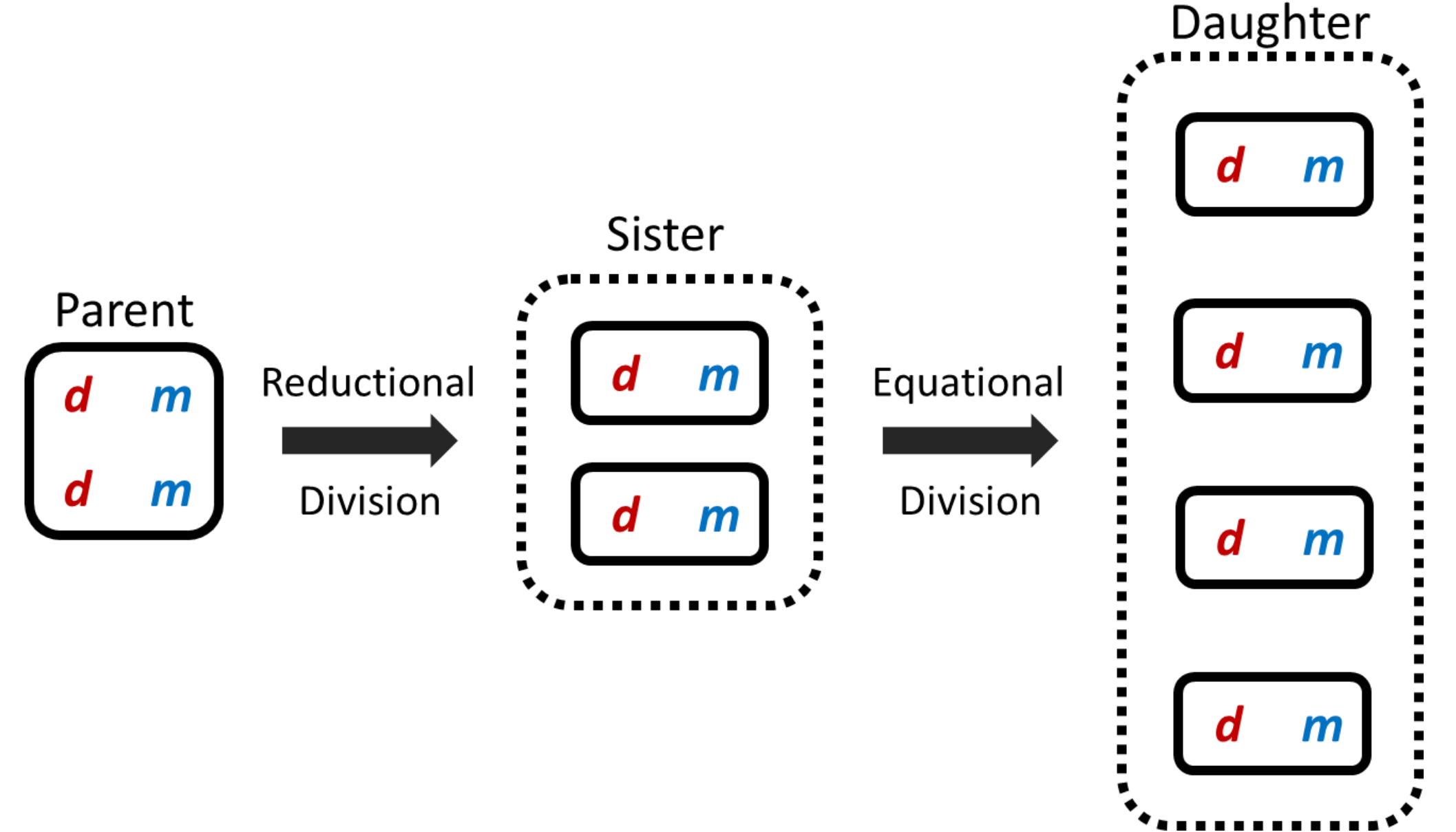 | 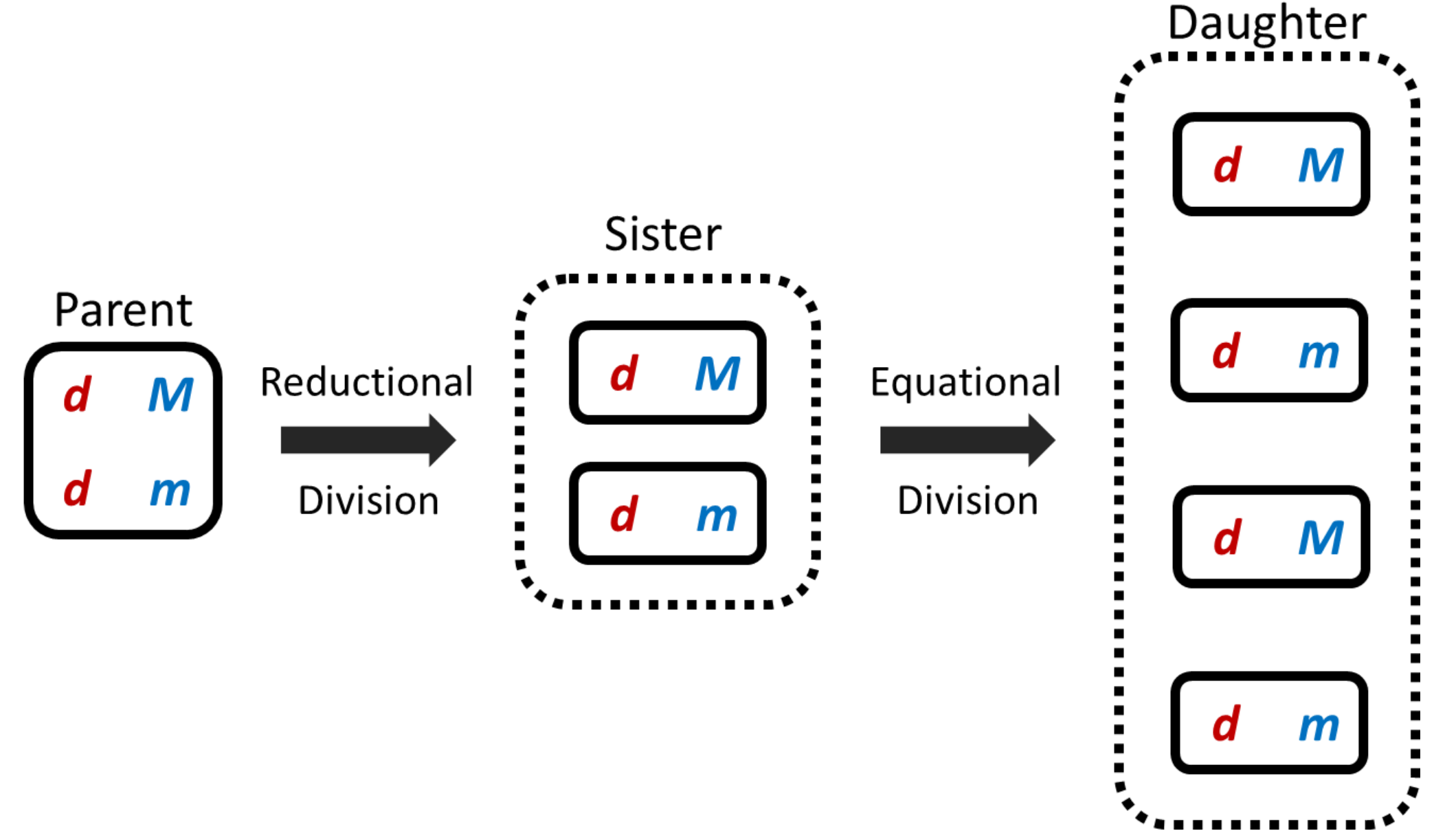 | 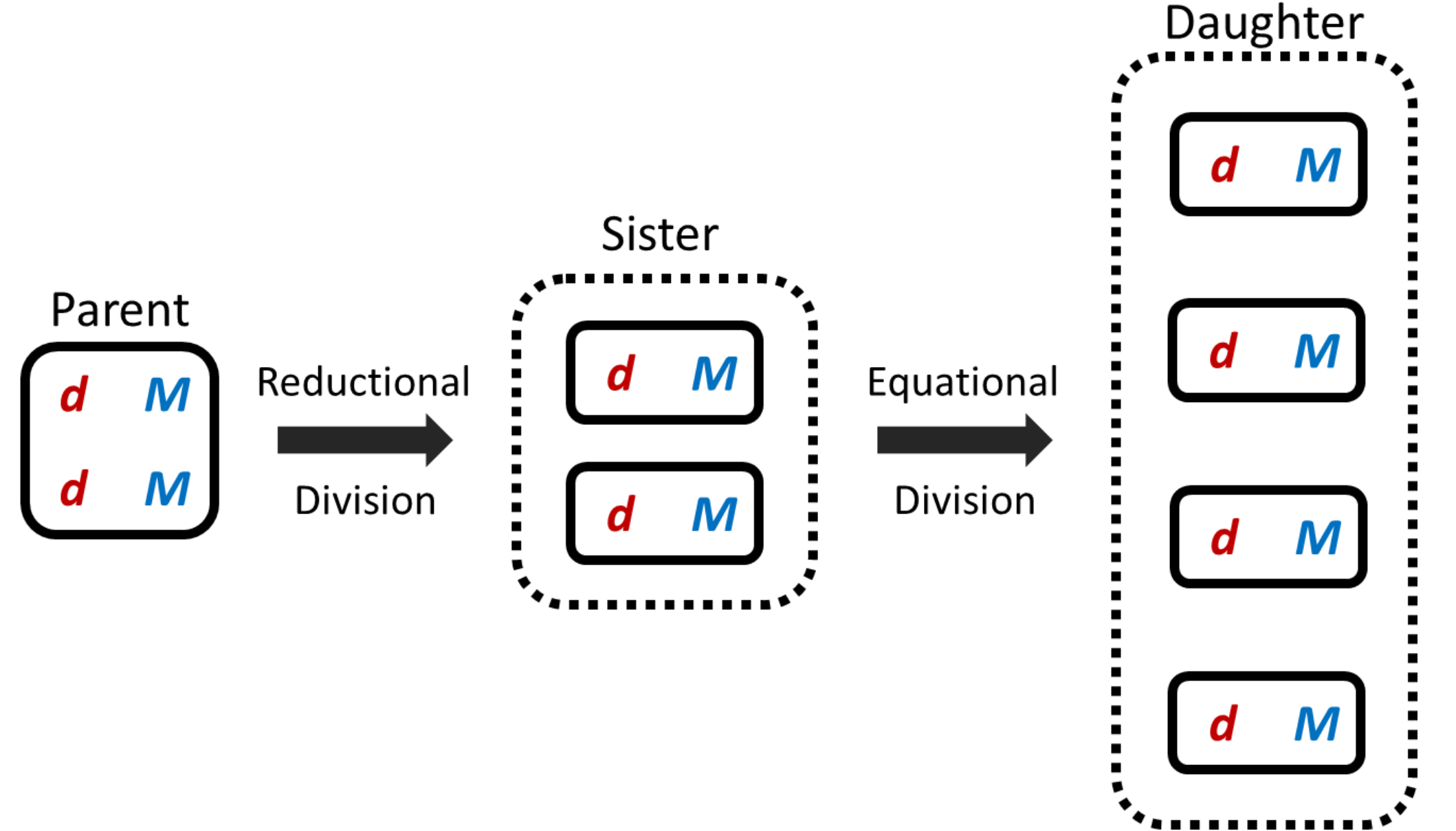 |

| B)   | $mm$                                                                                | $Mm$                                                                                 | $MM$                                                                                  |
|------|-------------------------------------------------------------------------------------|--------------------------------------------------------------------------------------|---------------------------------------------------------------------------------------|
| $DD$ | 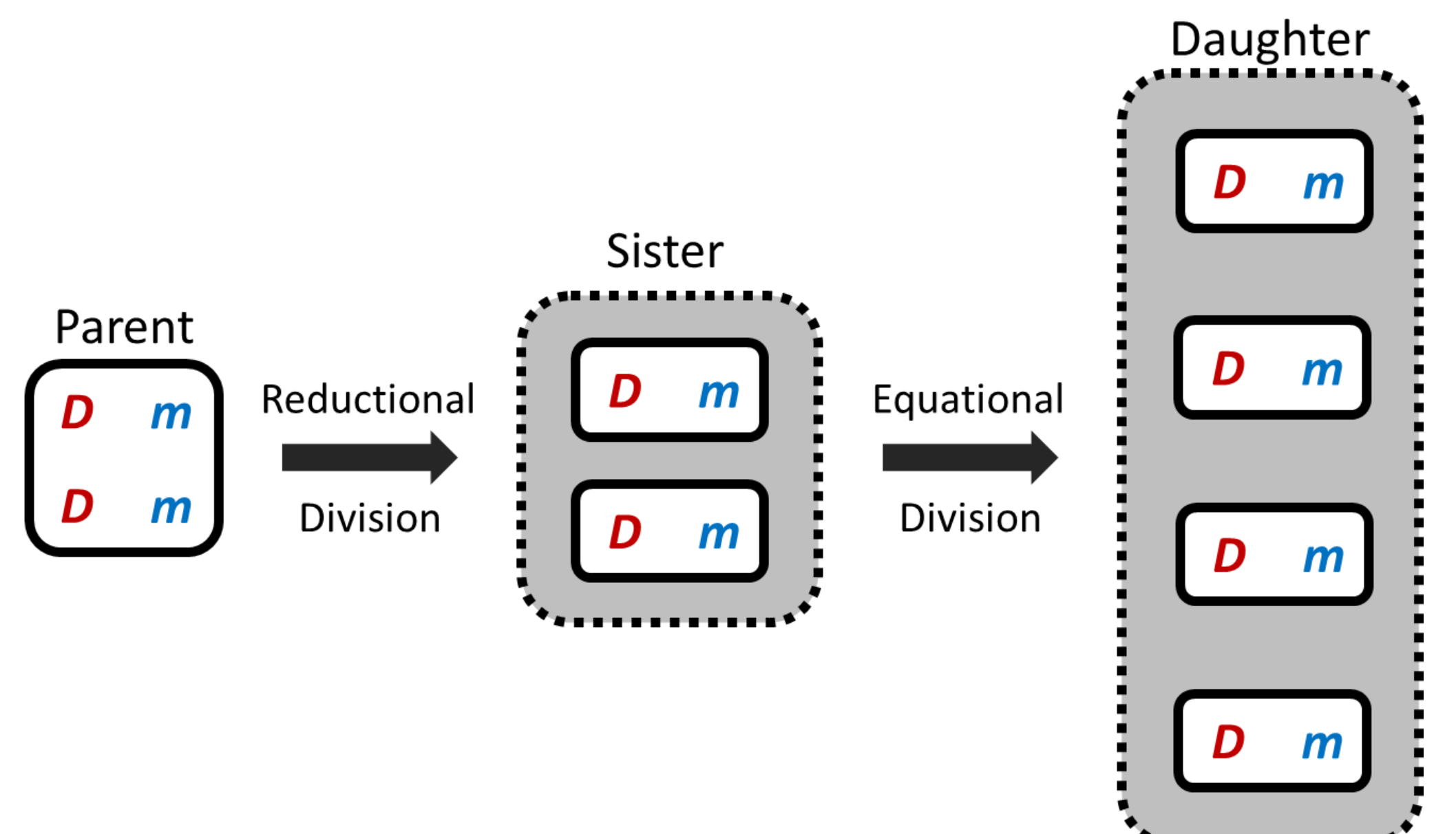   | 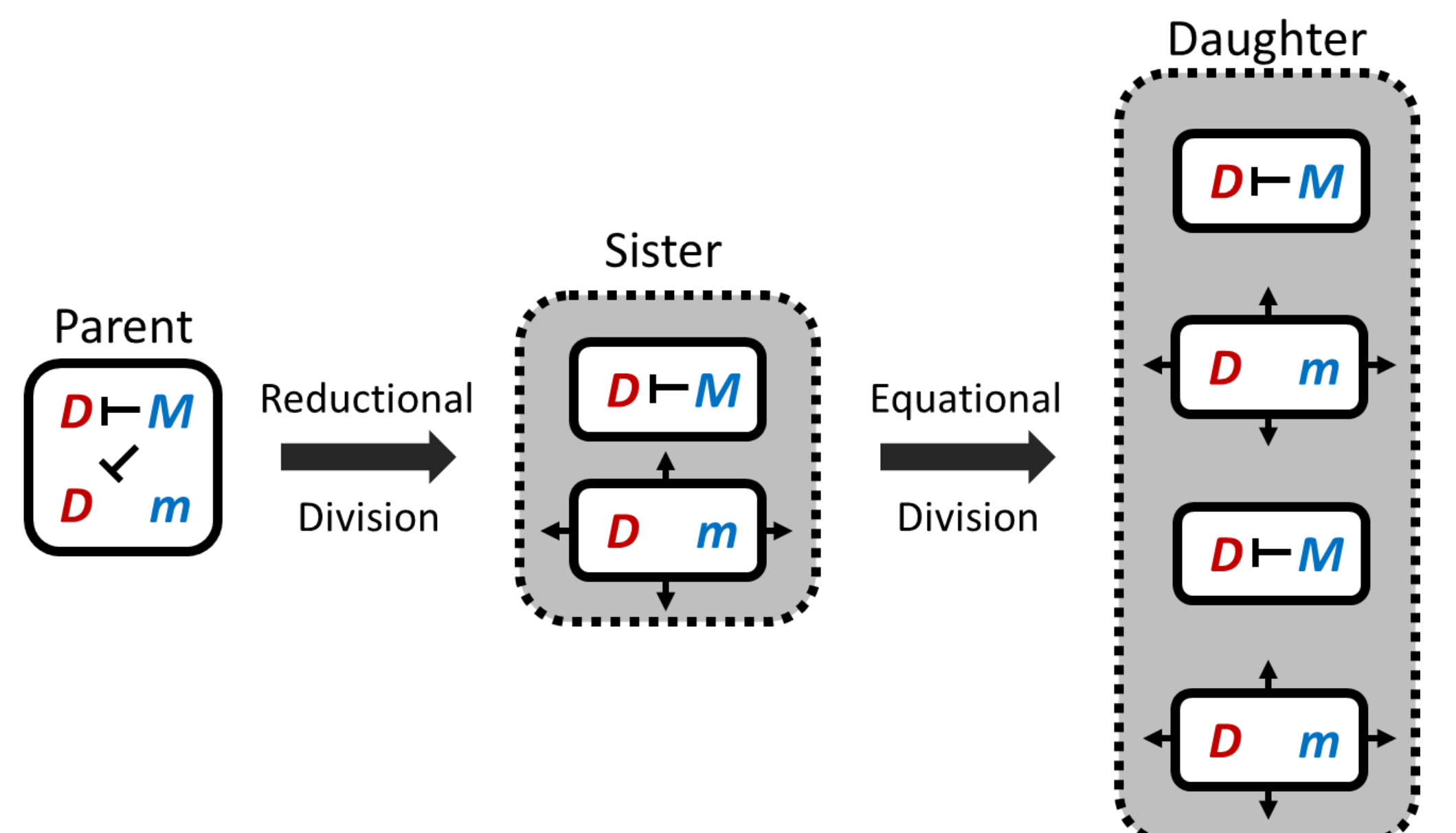   | 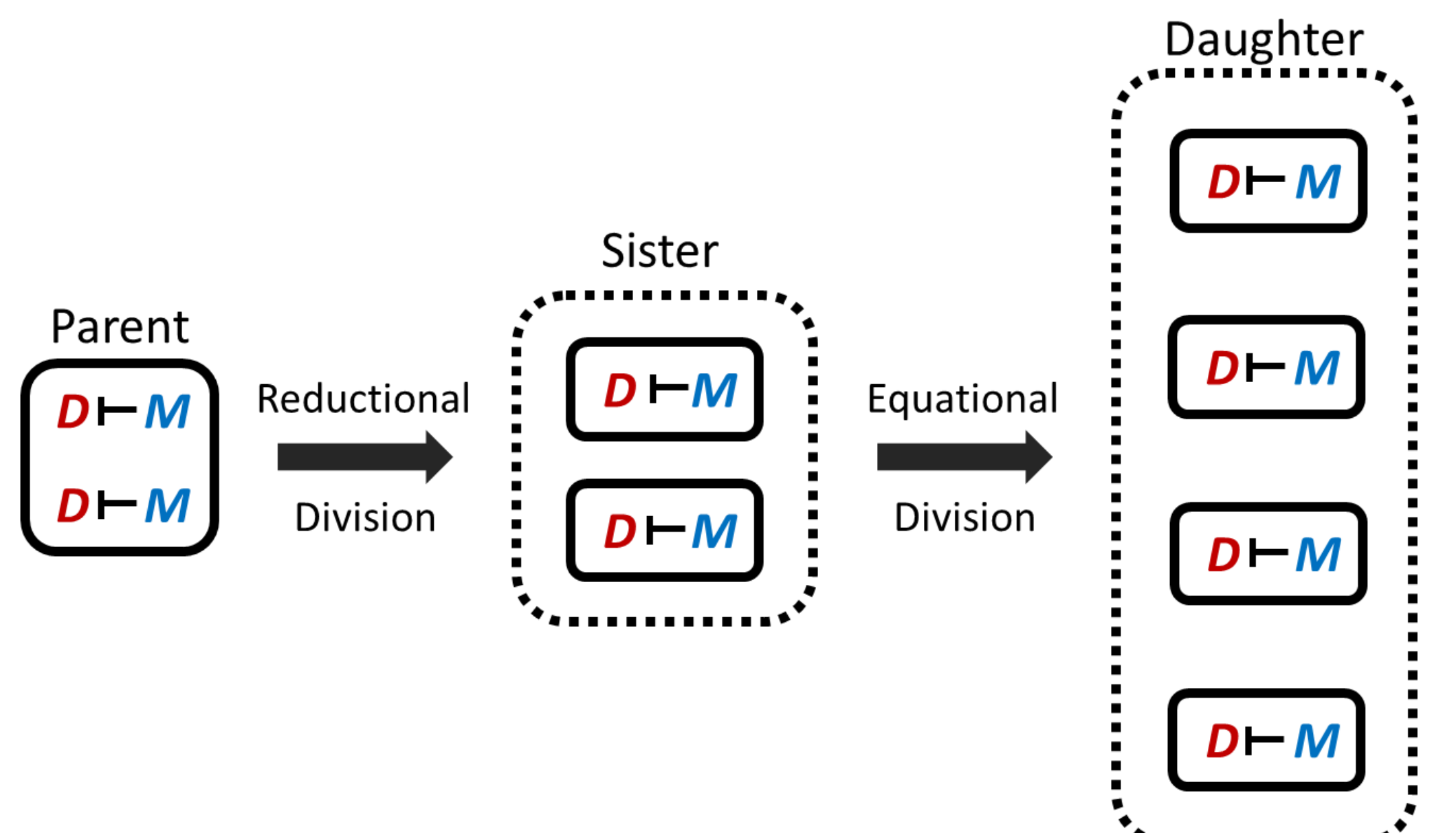   |
| $Dd$ | 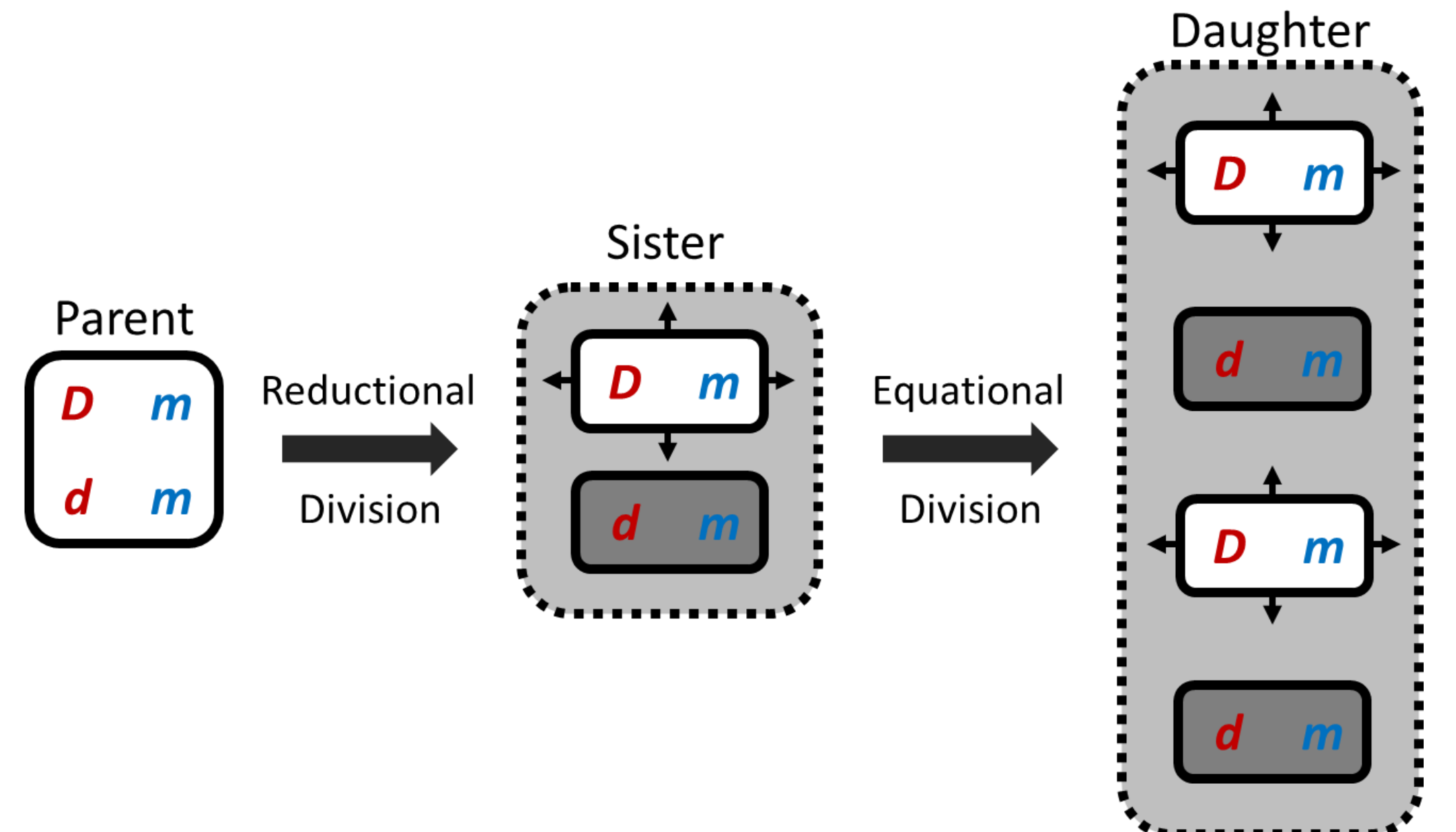 | 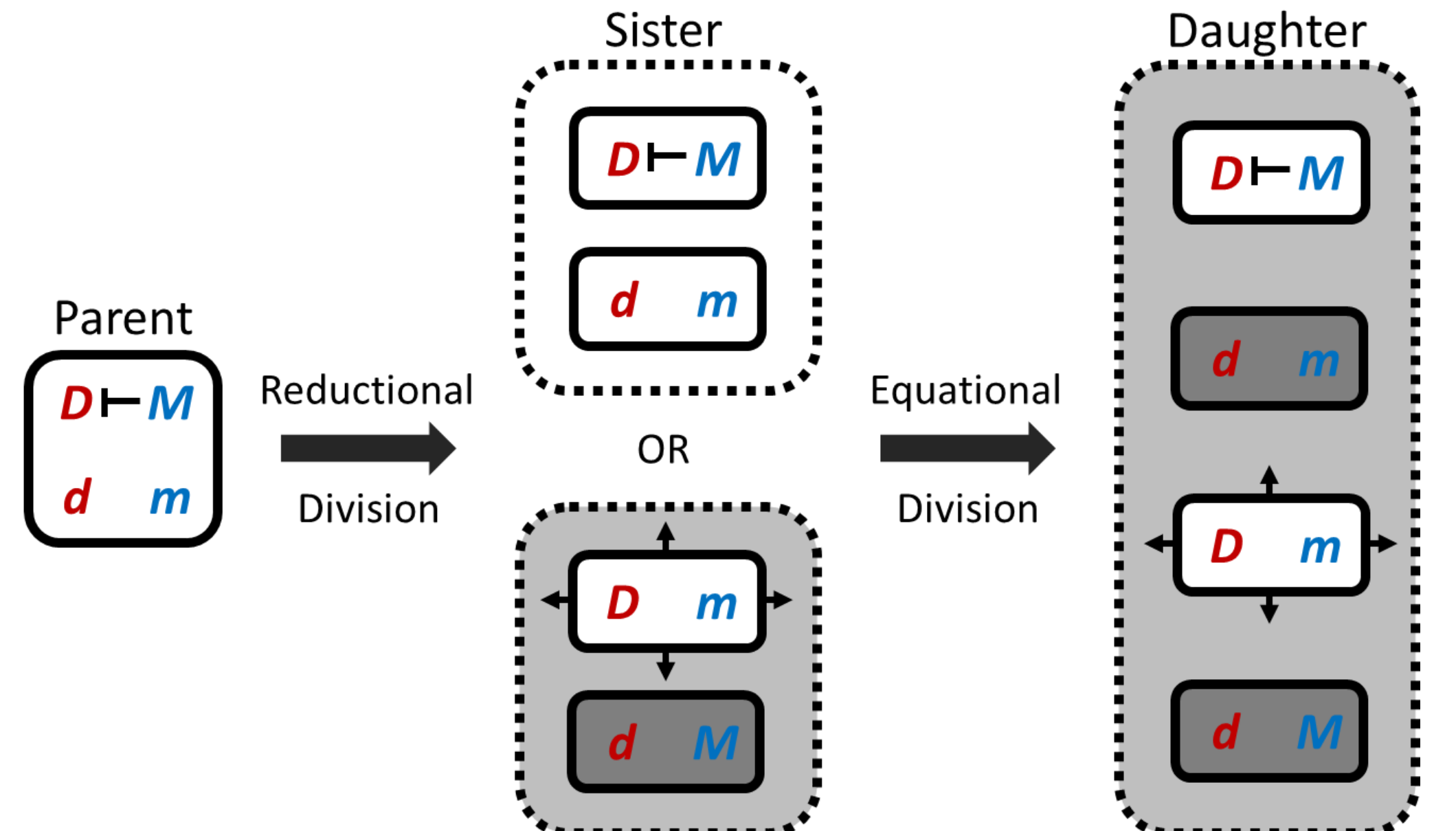 | 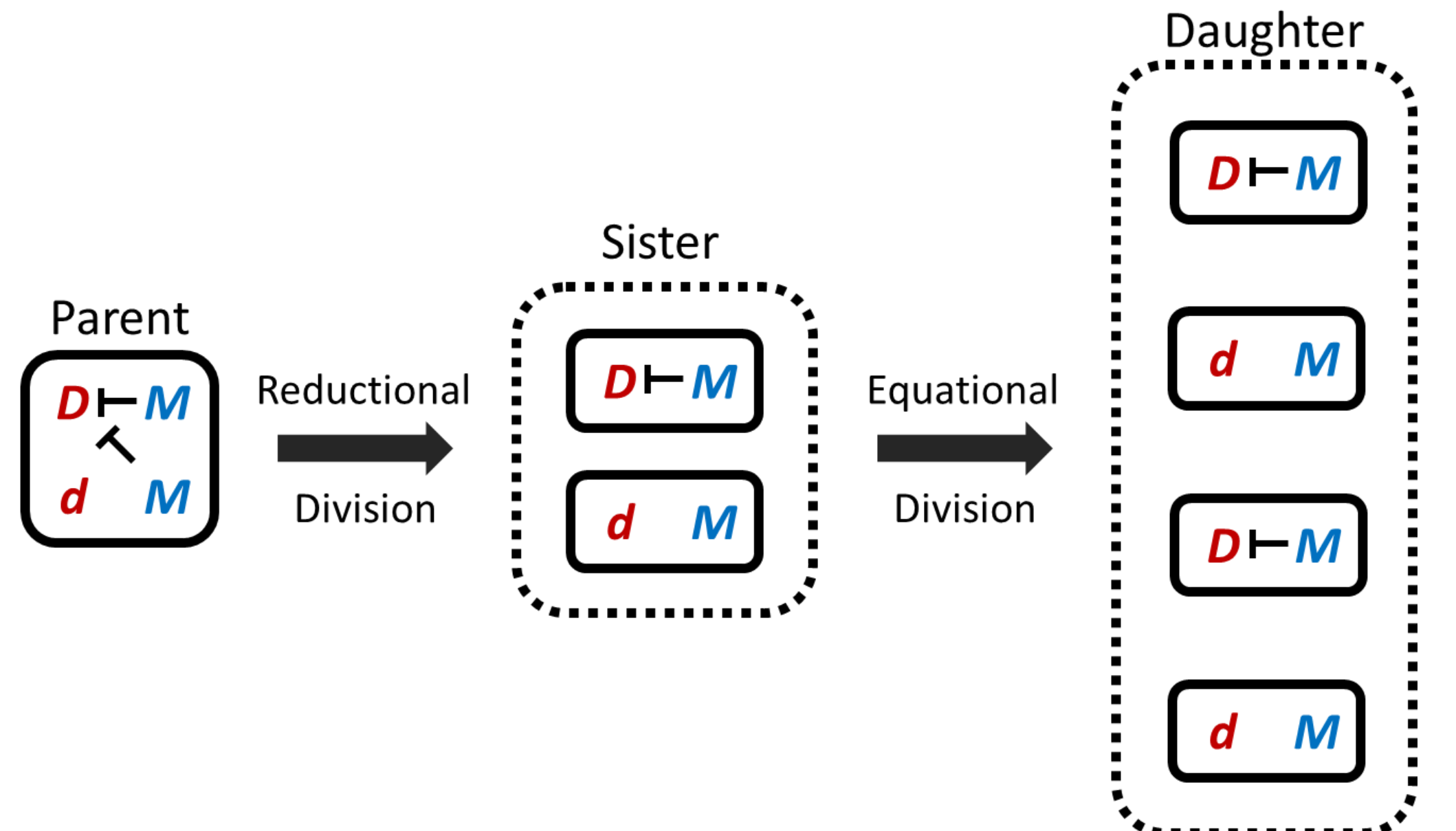 |
| $dd$ | 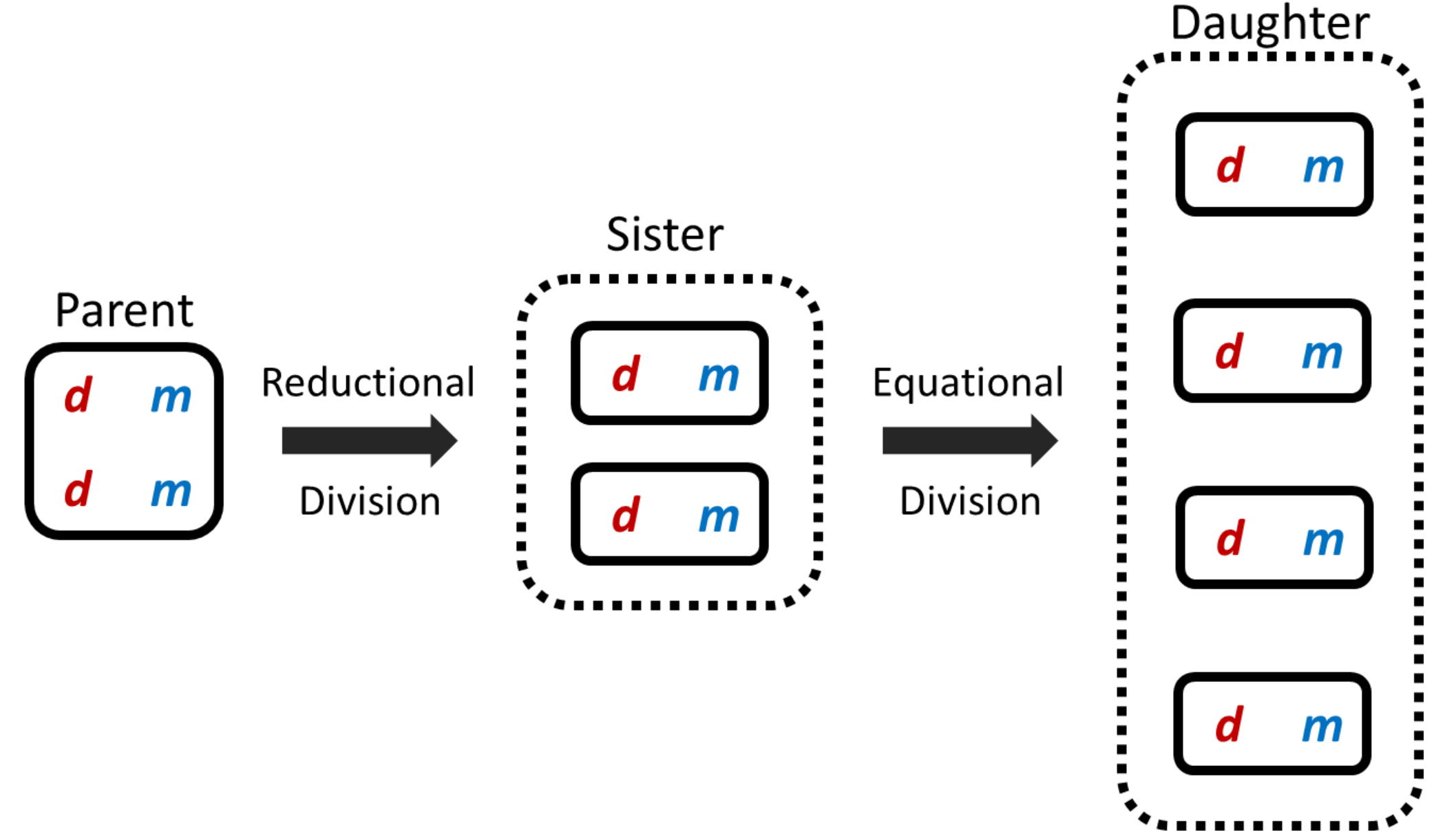 | 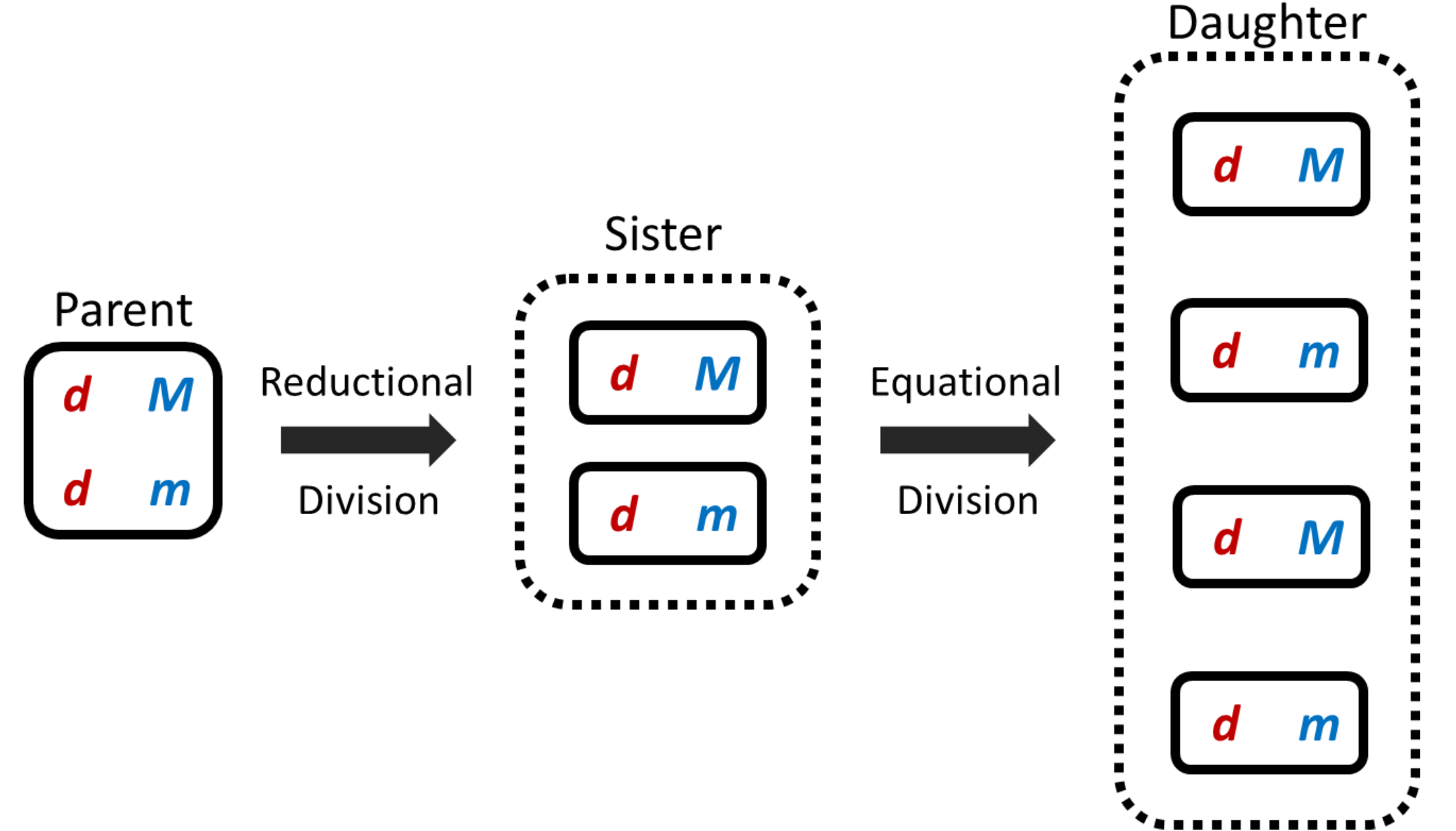 | 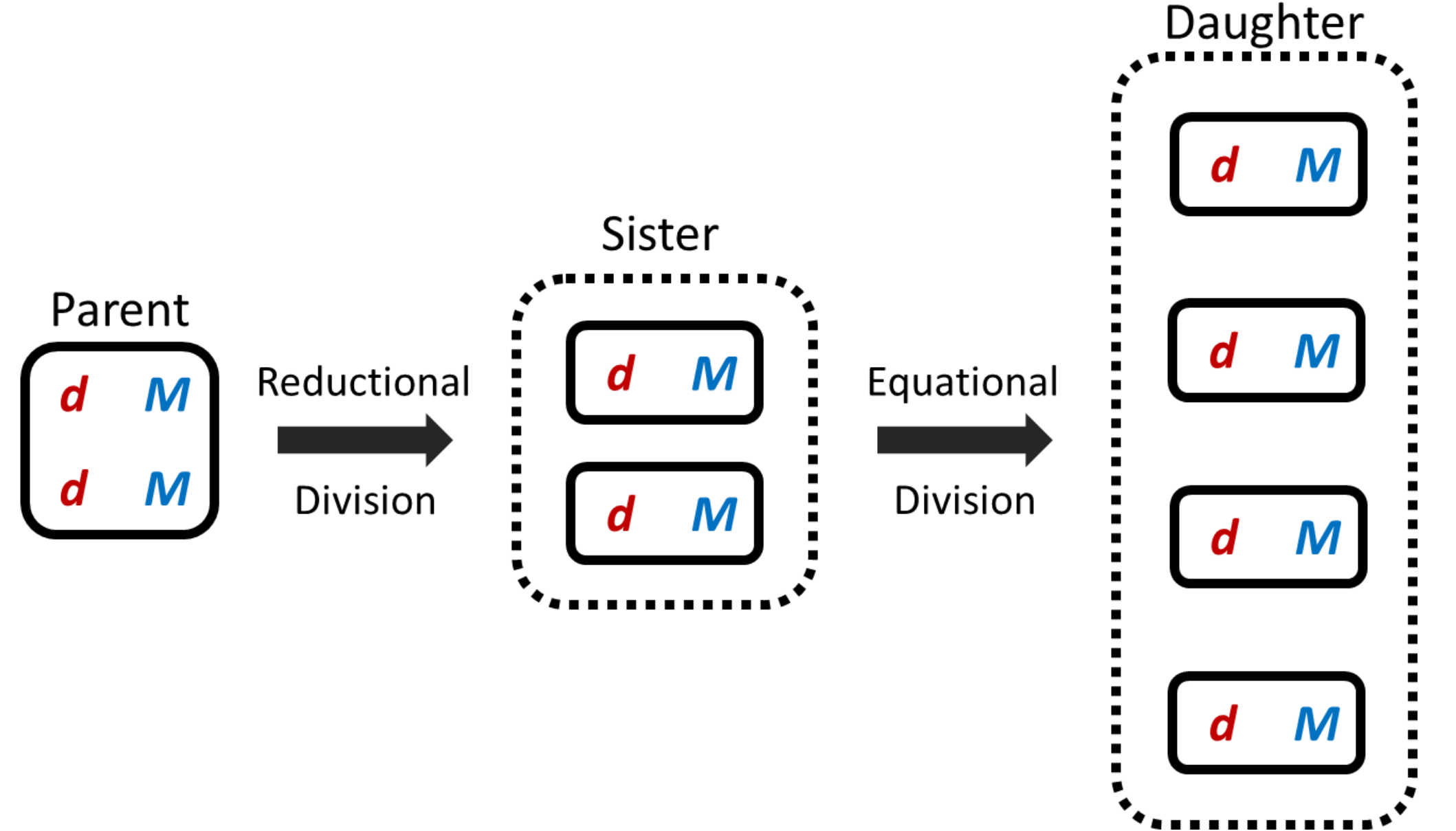 |

Supplement: Supplementary file 1 — Figure S1. Simplified diagrams of different types of gamete‐killing gene drive throughout the steps of the meiotic divisions across all genotypic combinations. [file EVL3-5-541-s001.pdf]
