# Supplementary material for: Evolutionary robustness of killer meiotic drives
Source: Evol Lett. 2021 Sep 12;5(5):541–50. doi: 10.1002/evl3.255 (PMC8484726; doi:10.1002/evl3.255)
Supplement: Supplementary file 2 — Table S1. Allelic fitness of drive, nondrive and resistant alleles in a single locus system. Table S2. Allelic fitness across pre‐, post‐ and meso‐meiotic killer scenarios in the interlocus systems. Table S3. Fitness of gametes produced by each of the two‐locus genotypes under pre‐meiotic killing. Table S4. Fitness of gametes produced by each of the two‐locus genotypes under post‐meiotic killing. Appendix A. Additional model description, explanation and analysis. Appendix B. Analysis of impact of linkage disequilibrium, recombination, and crossovers. [file EVL3-5-541-s002.pdf]

Supplement to ‘Evolutionary robustness of killer meiotic drives’

Authors: Madgwick, P. G.[1*] & Wolf, J. B.[1]

[1] Milner Centre for Evolution, Department of Biology and Biochemistry, University of Bath, UK

* Corresponding author: philipgmadgwick@gmail.com

Figure S1. Simplified diagrams of different types of gamete-killing gene drive throughout the steps of the meiotic divisions across all genotypic combinations.

Table S1. Allelic fitness of drive, nondrive and resistant alleles in a single locus system.

Table S2. Allelic fitness across pre-, post- and meso-meiotic killer scenarios in the interlocus systems.

Table S3. Fitness of gametes produced by each of the two-locus genotypes under pre-meiotic killing.

Table S4. Fitness of gametes produced by each of the two-locus genotypes under post-meiotic killing.

Table S5. Fitness of gametes produced by each of the two-locus genotypes under meso-meiotic killing.

Appendix A. Additional model description, explanation and analysis.

Appendix B. Analysis of impact of linkage disequilibrium, recombination, and crossovers.

Supplementary Figure 1. Simplified diagrams of different types of gamete-killing gene drives throughout the steps of the meiotic divisions across all genotypic combinations (indicated in a tabular form with respect to the drive and modifier loci). Different cells are presented with solid outlines, and different interactions between cells are presented with dashed outlines (except for the possible interaction between the parent cell and the sister cells it produced). Alleles at the drive locus are presented in red (D = drive and d = nondrive) and alleles at the modifier locus are presented in blue (M = modifier and m = nonmodifier). A drive allele for a poison-antidote pair can distort its transmission to the next generation by producing a poison (indicated by out-facing arrows from the producer cell) that leads to the presence of the poison within that given interaction (indicated by the light-grey shading in dashed outlined box). The presence of the poison kills gametes that do not also produce the poison with cell death indicated by the dark-grey shading in solid outlined boxes). The modifier allele (M) only has an effect on the drive (D) allele if it is within the same cell, which is indicated by the turnstile symbol ⊢ that indicates that the drive allele has blocked some function of the drive allele (with different scenarios illustrated in A and B). As such, in the double heterozygote scenario presented here, there is no potential for pre-meiotic killing, where the parent cell produces the poison that kills sister or daughter cells, because the modifier blocks poison production. However, there is potential for meso-meiotic killing between sister cells (of which there are two potential genotypic combinations represented by different interactions in dashed boxes) and post-meiotic killing between daughter cells (ignoring the potential for daughter cells to kill sister cells in this simplified diagram). **A)** The case of a fully suppressing modifier that blocks poison and antidote production by the drive allele, rendering it susceptible to the poison produced by other cells. **B)** The scenario of a partially suppressing modifier that blocks poison production only, and so remains resistant to the poison produced by other cells.

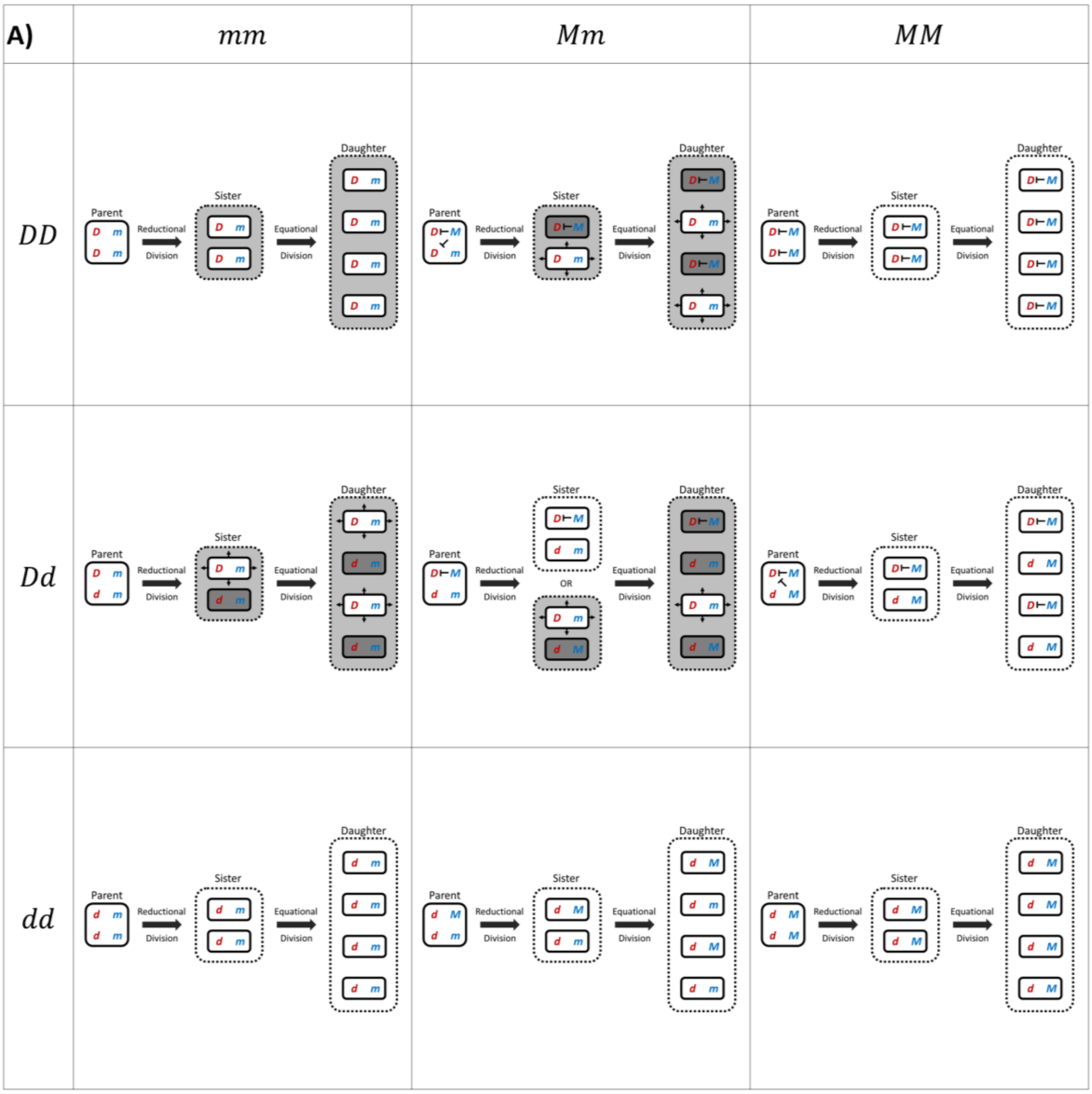
A)
mm
Mm
MM
DD
Dd
dd
Parent
Reductional Division
Sister
Equational Division
Daughter
OR

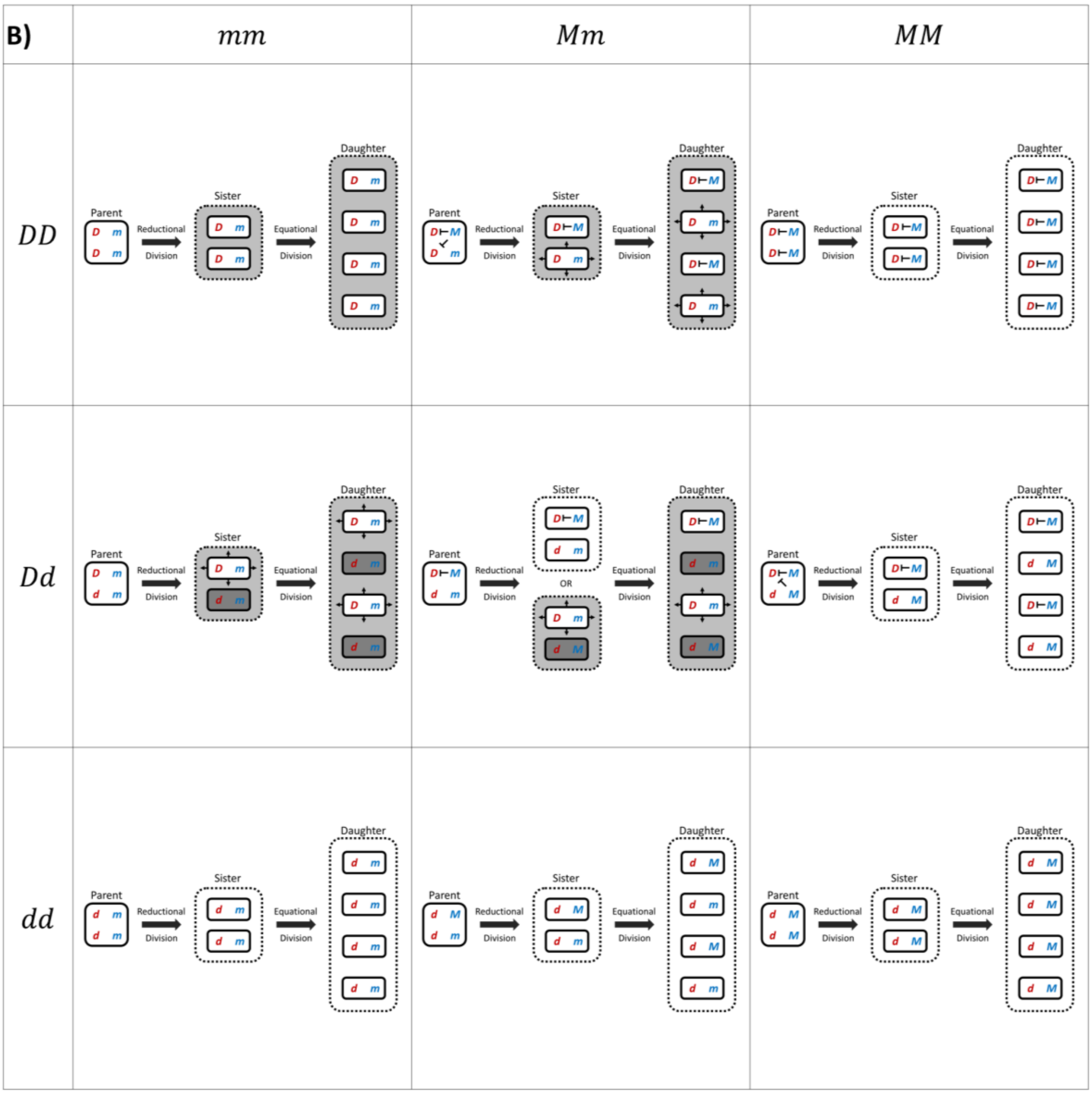
B)
mm
Mm
MM
DD
Dd
dd
Parent
Reductional Division
Sister
Equational Division
Daughter
OR

Table S1. Allelic fitness of drive (D), nondrive (d) and resistant (δ) alleles in the intralocus system.

Allele	Fitness
D	$1 - s(f_D + f_d h) + f_d e(1 - hs)$
d	$1 - f_D\big(hs + e(1 - hs)\big)$
δ	$1 - f_D hs$

Table S2. Allelic fitness across pre-, post- and meso-meiotic killer scenarios in the interlocus systems. The fitness of the drive (D) and nondrive (d) alleles are unchanged by the type of suppression (full or partial), whilst the fitness of the modifier (M) and nonmodifier (m) allele are given specific to the type of suppression.

**Allele**	**Pre-meiotic killer**	**Post-meiotic killer**	**Meso-meiotic killer**
D	$1+f_m{}^2\left(-f_D s+f_d\left(-hs+e(1-hs)\right)\right)$	$1-f_D\left(2f_M f_m h+f_m{}^2\right)s$ $+f_d\left(1-f_M{}^2\right)\left(-hs+e(1-hs)\right)$	$1-f_D\left(2f_M f_m h+f_m{}^2\right)s$ $+f_d f_m\left(-hs+e(1-hs)\right)$
d	$1-f_D f_m{}^2\left(hs+e(1-hs)\right)$	$1-f_D\left(1-f_M{}^2\right)\left(hs+e(1-hs)\right)$	$1-f_D f_m\left(hs+e(1-hs)\right)$
M (full)	1	$1-f_m\left(1-f_d{}^2\right)\left(hs+e(1-hs)\right)$	$1-f_D f_m\left(hs+e(1-hs)\right)$
m (full)	$1-f_m(f_D{}^2+2f_D f_d h)s$	$1-f_m\left(f_D{}^2+2f_D f_d h\right)s$ $+f_M\left(1-f_d{}^2\right)\left(-hs+e(1-hs)\right)$	$1-f_m\left(f_D{}^2+2f_D f_d h\right)s$ $+f_D f_M\left(-hs+e(1-hs)\right)$
M (part.)	1	$1-f_m\left(1-f_d{}^2\right)hs$	$1-f_D f_m\left(hs+f_d e(1-hs)\right)$
m (part.)	$1-f_m(f_D{}^2+2f_D f_d h)s$	$1-(f_D{}^2(f_M h+f_m)+2f_D f_d h)s$	$1-f_m\left(f_D{}^2+2f_D f_d h\right)s$ $+f_D f_M\left(-hs+f_d e(1-hs)\right)$

Table S3. Fitness of gametes produced by each of the two-locus genotypes under pre-meiotic killing, where the drive allele produces the poison in the parent (or somatic) cell making the modifier allele dominant to the nonmodifier allele (which is equivalent with modifiers for both full and partial suppression). The 'Genotype' column lists the nine possible unordered genotypes, whilst the 'gametes' column refers to the alleles within interacting cell types. The 'Fitness' column gives the production of the different gametes contributed to the next generation by that genotype. There are two loci (drive and modifier) that each have two alleles: the drive locus has a drive allele D, which encodes a poison and antidote, and nondrive allele d, while the modifier locus has a modifier allele M, which blocks the production of both the poison and antidote, and a nonmodifier allele m. Within an interaction, when the poison is produced it leads to a toxicity effect (s) to all gametes and a transmission advantage ($+e$) for those gametes that also produce the antidote, alongside a corresponding transmission disadvantage ($-e$) for those gametes that do not produce the antidote. Shading is used to separate the sets of gametes produced by each individual's diploid genotype.

**Genotype**	**Gametes**	**Fitness**
$DDMM$	DM	1
$DDMm$	DM	1
	Dm	1
$DDmm$	Dm	$1-s$
$DdMM$	DM	1
	dM	1
$DdMm$	DM	1
	Dm	1
	dM	1
	dm	1
$Ddmm$	Dm	$(1+e)(1-hs)$
	dm	$(1-e)(1-hs)$
$ddMM$	dM	1
$ddMm$	dM	1
	dm	1
$ddmm$	dm	1

Table S4. Fitness of gametes produced by each of the two-locus genotypes under post-meiotic killing, where the drive allele produces the poison in the daughter cells (*i.e.* the gametes) making the modifier allele (for either full or partial suppression) recessive to the nonmodifier allele. The 'Genotype' column lists the nine possible unordered genotypes, whilst the 'gametes' column refers to the alleles within interacting cell types. The 'Fitness' column gives the production of the different gametes contributed to the next generation by that genotype. There are two loci (drive and modifier) that each have two alleles: the drive locus has a drive allele D, which encodes a poison and antidote, and nondrive allele d, while the modifier locus has a modifier allele M and a nonmodifier allele m. Within an interaction, when the poison is produced it leads to a toxicity effect (s) to all gametes and a transmission advantage ($+e$) for those gametes that also produce the antidote, alongside a corresponding transmission disadvantage ($-e$) for those gametes that do not produce the antidote. Shading is used to separate the sets of gametes produced by each individual's diploid genotype. **A)** The modifier allele, M, causes full suppression of the drive locus by blocking the production of the poison and the antidote. **B)** The modifier allele, M, causes partial suppression of the drive locus by blocking the production of the poison but not the antidote.

A)

**Genotype**	**Gametes**	**Fitness**
$DDMM$	DM	1
$DDMm$	DM	$(1-e)(1-hs)$
	Dm	$(1+e)(1-hs)$
$DDmm$	Dm	$1-s$
$DdMM$	DM	1
	dM	1
$DdMm$	DM	$(1-e)(1-hs)$
	Dm	$(1+3e)(1-hs)$
	dM	$(1-e)(1-hs)$
	dm	$(1-e)(1-hs)$
$Ddmm$	Dm	$(1+e)(1-hs)$
	dm	$(1-e)(1-hs)$
$ddMM$	dM	1
$ddMm$	dM	1
	dm	1
$ddmm$	dm	1

B)

Genotype	Gametes	Fitness
$DDMM$	DM	1
$DDMm$	DM	$1-hs$
	Dm	$1-hs$
$DDmm$	Dm	$1-s$
$DdMM$	DM	1
	dM	1
$DdMm$	DM	$(1+e)(1-hs)$
	Dm	$(1+e)(1-hs)$
	dM	$(1-e)(1-hs)$
	dm	$(1-e)(1-hs)$
$Ddmm$	Dm	$(1+e)(1-hs)$
	dm	$(1-e)(1-hs)$
$ddMM$	dM	1
$ddMm$	dM	1
	dm	1
$ddmm$	dm	1

Table S5. Fitness of gametes produced by each of the two-locus genotypes under meso-meiotic killing, where the drive allele produces the poison in the sister cells (for either full or partial suppression). The ‘Genotype’ column lists the nine possible unordered genotypes, whilst the ‘gametes’ column refers to the alleles within interacting cell types. The ‘Fitness’ column gives the production of the different gametes contributed to the next generation by that genotype. There are two loci (drive and modifier) that each have two alleles: the drive locus has a drive allele D, which encodes a poison and antidote, and nondrive allele d, while the modifier locus has a modifier allele M, which blocks the production of both the poison and antidote, and a nonmodifier allele m. Within an interaction, when the poison is produced it leads to a toxicity effect (s) to all gametes and a transmission advantage ($+e$) for those gametes that produce the antidote, alongside a corresponding transmission disadvantage ($-e$) for those gametes that do not produce the antidote. Shading is used to separate the sets of gametes produced by each individual’s diploid genotype. **A)** The modifier allele, M, causes full suppression of the drive locus by blocking the production of the poison and the antidote. **B)** The modifier allele, M, causes partial suppression of the drive locus by blocking the production of the poison but not the antidote.

A)

**Genotype**	**Gametes**	**Fitness**
$DDMM$	DM	1
$DDMm$	DM	$(1-e)(1-hs)$
	Dm	$(1+e)(1-hs)$
$DDmm$	Dm	$1-s$
$DdMM$	DM	1
	dM	1
$DdMm$	DM	1
	Dm	$(1+e)(1-hs)$
	dM	$(1-e)(1-hs)$
	dm	1
$Ddmm$	Dm	$(1+e)(1-hs)$
	dm	$(1-e)(1-hs)$
$ddMM$	dM	1
$ddMm$	dM	1
	dm	1
$ddmm$	dm	1

B)

Genotype	Gametes	Fitness
$DDMM$	DM	1
$DDMm$	DM	$1 - hs$
	Dm	$1 - hs$
$DDmm$	Dm	$1 - s$
$DdMM$	DM	1
	dM	1
$DdMm$	DM	1
	Dm	$(1 + e)(1 - hs)$
	dM	$(1 - e)(1 - hs)$
	dm	1
$Ddmm$	Dm	$(1 + e)(1 - hs)$
	dm	$(1 - e)(1 - hs)$
$ddMM$	dM	1
$ddMm$	dM	1
	dm	1
$ddmm$	dm	1

Appendix A. Additional model description, explanation, and analysis

To support the brief presentation of the model in the main text we provide a further description and discussion of several of the claims and assumptions made therein. These various points are described, explained, and analysed under subheadings in the order that these issues arise in the main text.

Assumption that poison-antidote and killer-target drive systems are equivalent in our model

In the *Introduction* we comment that a poison-antidote system is equivalent to a killer-target drive system under the model scenarios we examine such that our model can apply to either scenario. In the poison-antidote drive system, a gene encodes a diffusible poison and its non-diffusible antidote. The poison diffuses away from cells that produce it, but the antidote remains cell bound. Consequently, the poison only kills cells that do not produce the antidote. Alternatively, in a killer-target drive system, a gene encodes a specific killer element but not its target element, and cells with the killer element only kill other cells that have the target element. The central difference between poison-antidote and killer-target drive systems is whether gametes are killed based on the absence of the antidote or the presence of the target element, respectively. The poison-antidote mechanism can be conceptualised as killing based on not-self recognition because any entity with the gene that does not encode the antidote would be susceptible to the poison. Similarly, the killer-target mechanism can be conceptualised as non-self recognition because any entity without the target element (and without the killer element) would be immune to the killer element. The poison-antidote and killer-target drive systems are equivalent in our single-locus model because the drive locus only has two variants: the drive and nondrive alleles. As such, a cell with the drive allele either poisons cells that only have the nondrive allele because they do not have the right antidote, or kills cells that only have the nondrive allele because they have the target element.

The two types of drive systems could potentially show different properties when the drive locus has multiple drive or nondrive alleles (which we do not consider in our analysis). For example, if each drive allele that encodes a single poison and a single antidote then this would imply that each drive

allele would be susceptible to the poison from other drive allele(s). By contrast, a killer-target system would imply that each drive allele would be immune to the killer element from the other drive allele(s) because, by being a different allele, having a different killer element would imply not having the target element. Consequently, each drive allele would have a transmission advantage against one only one other allele (*i.e.*, against the allele that possesses the target). It is unlikely that the target element could be the consequence of an allele at a different (freely-recombining) locus because then a cell with the killer element would often kill itself, which could prevent it from being favoured by natural selection. However, it is possible that a different locus could act as a modifier of the drive locus, which can alter the equivalency of the two types of drive. Following the terminology of the main text, a modifier is assumed to only influence the expression of a drive allele when they are in the same cell by blocking poison and/or antidote production. Arguably, it does not make good sense to consider a modifier that blocks the killer element's expression and also leads to the expression of the target element, which means that the killer-target system may be better described as only having partially-suppressing modifiers that block poison production. However, as the main text considers both, it provides a general description of both the poison-antidote and killer-target drive mechanisms. Additionally, perhaps it would be possible for a modifier to make a cell not express the target element, whether or not it contains the drive allele, which the analysis in the main text does not permit. Although such a modifier allele would evolve at a separate locus, this would make it phenotypically similar to a resistant allele, which the main text describes (and as is shown below) as being able to invade any drive system. Whether or not such a modifier would be possible within the biology of killer-target systems remains unclear. Yet, as the *Introduction* states, we know that this cannot be sufficient to explain why some meiotic drivers are able to evade suppression whilst others are not because ancient meiotic drivers have been identified that use either mechanism.

Assumption that drive does not affect sex ratio

In-keeping with the classic model that the main text builds upon (Leigh, 1971), we assume that the drive locus is autosomal and has no effect on sex ratio. There are many meiotic drivers on sex

chromosomes that alter sex ratio (Jaenike, 2001), which affords them a predictable set of evolutionary properties. As the sex ratio becomes more unequal, the selective advantage for a modifier (or resistant allele) that prevents sex chromosome drive becomes larger (Hamilton, 1967; see also Holman *et al.*, 2015). Consequently, sex chromosome drive generally results in either suppression or population extinction (Helleu *et al.*, 2015). However, it is possible for a meiotic driver on a sex chromosome to have a small effect on sex ratio, which could enable it to persist without resulting in such extreme outcomes (Burt & Trivers, 2008). While such meiotic drive could have important consequences in nature, their evolutionary dynamics are well understood and are outside of our framework (because the properties of the selection dynamics on autosomes are fundamentally different).

Assumption that drive is not sex-limited

We assume that the drive effect is not sex-limited (*i.e.* only taking place in females or males), although this could be easily incorporated into the model. There are several examples of killer meiotic drivers that are indeed not sex-limited (Rick, 1966; Endo, 1990; Turner & Perkins, 1991; Hu *et al.*, 2017; Bravo Núñez *et al.*, 2018). However, the majority of examples of killer meiotic drivers are limited to sperm killing in males, which reflects the fact that meiosis in oogenesis produces nonreproductive polar body cells and is often compartmentalised to prevent interactions between egg cells (Burt & Trivers, 2008). Due to the formation of polar bodies, the cost of female-only meiotic drive could conceivably be zero because the same number of reproductive cells may be produced if the drive mechanism never harms the cell that contains the drive allele. Any side-effects or errors from the drive mechanism would introduce a toxicity effect, as described in the main text. The compartmentalisation of egg cells from independent meiotic divisions limits the scope of post-meiotic killing, which may have evolved as a general suppression mechanism. By contrast, the meiotic divisions that produce the much smaller sperm cells are often pooled to store the sperm together. The molecular details of meiosis in different sexes and species could provide avenues for future research. Yet, for the purposes of our analysis, which assumes equal sex ratio, sex-limited meiotic drive does not alter the fundamental evolutionary properties of the system and could easily be incorporated by simply multiplying the effects

of the drive by ½, which accounts for the fact that meiotic drive only occurs in half of the individuals (*i.e.* of one sex) within the population. This change would not alter the evolutionary predictions, it would simply slow the rate of evolutionary spread of the meiotic driver and any favoured modifiers.

Further details of the basic single-locus two-allele model of meiotic drive

The basic model of meiotic drive that is briefly outlined in the main text builds from a classic model (Leigh, 1971), which we outline in more detail here. At a single locus, a drive allele (D, with frequency f_D) competes against a nondrive allele (d, with frequency f_d). The drive allele produces a poison that diffuses away from cells that produce it and an antidote that remains cell bound. The nondrive allele (dd) homozygote is assigned a baseline fitness of 1. In the drive allele (DD) homozygote fitness is reduced by a toxicity effect (s, scaled to vary between 0 and 1) because the poison kills or damages their gametes in spite of the antidote. This accounts for the fact that the poison is costly in that it is capable of killing some of the drive allele containing gametes, even though they have an antidote against the poison. In the Ddheterozygotes, fitness is reduced by the product of the toxicity effect (s) and the degree of dominance of the toxicity effect h (*i.e.* the total toxicity effect is hs; where h can vary between 0 and 1), where the latter captures the dosage effect of the poison. The drive allele could potentially suffer reduced fitness if it is in linkage with deleterious mutations, which occurs when meiotic drivers are formed from large non-recombining regions (*e.g. SD*, *t*-haplotype). However, this would differ from the toxicity cost we consider since it would only reduce fitness of the drive allele carrying cells (and so reduces the mean fitness of drive allele carrying cells independent of the toxicity effect or transmission effect). Therefore, we do not consider the potential impact of this scenario (though it would logically simply reduce the ability of a driver to invade and spread). The fact that the toxicity effect is suffered equally by all gametes (regardless of whether they are carrying the drive allele or the nondrive allele) implies that the diffusible poison is costly and the non-diffusible antidote is cost-free (since it would otherwise appear as a cost specific to cells containing the drive allele). The interaction between the drive and nondrive alleles in Dd heterozygotes produces a transmission effect (e, scaled to

vary between 0 and 1) from the selective killing of gametes with the nondrive allele, which secures a transmission advantage ($+e$) for the drive allele whilst imposing an equal and opposite transmission disadvantage ($-e$) on the nondrive allele. The transmission effect (e) only arises in Dd heterozygotes and fully describes the possible range of the selective killing of gametes on the scale from 0 for a fair meiosis to a maximum of 1 (where only drive allele carrying gametes survive). Consequently, in Dd heterozygotes, the components of fitness from the toxicity effect (s) and transmission effect (e) are multiplicative, with the former determining the number of viable gametes produced and the latter determining the proportion of those gametes that contains the focal allele.

Assuming an infinite panmictic population, the overall fitness of the drive allele is the frequency-weighted average of its fitness when homozygous (where it suffers the toxicity effect) and its fitness when heterozygous (where it pays the toxicity effect weighted by its dominance h, but also gains the transmission advantage), with corresponding implications for the nondrive allele (see Table S1). To understand the conditions that favour the drive allele, we can derive the change in the frequency of the drive allele from its covariance with relative fitness:

$$\Delta f_D = f_D f_d[e(1-hs) - s(f_D + h(1-2f_D))]/\bar{w} \qquad \text{(S1)}$$

Therefore, the drive allele can invade whenever:

$$e(1-hs) - hs > 0 \qquad \text{(S2)}$$

When the toxicity effect is dominant ($h = 1$), equation (2) recapitulates a classic result (Leigh, 1971) that the drive allele is always able to spread when the product of the fitness components from the toxicity and transmission effects are greater than one (*i.e.* $(1+e)(1-s) > 1$; or $e > s/(1-s)$; see Figure 1A in the main text). Consequently, the drive allele is more likely to be favoured (and selection favouring

it would be stronger) the smaller the toxicity effect (s) and the larger the transmission effect (e). Relaxing the assumption of dominance ($h < 1$, but especially $h < ½$) enables the drive allele to spread when the toxicity effect (s) is larger and the transmission effect (e) is smaller (Figure 1B in the main text).

Drive systems can potentially harbour polymorphism at the drive locus. The equilibrium can be solved by finding the drive allele frequency where the change in drive allele frequency is zero ($\Delta f_D = 0$). There is one non-trivial (polymorphic) stable equilibrium for the drive locus:

$$\widehat{f_D} = \frac{e(1 - hs) - hs}{s(1 - 2h)} \tag{S3}$$

The numerator is the invasion criterion (see eqn. S2) and must be greater than zero for the drive allele to invade. For polymorphism, drive allele frequency must be within the range of 0 and 1, and so a polymorphic equilibrium is only possible when there is a large but recessive toxicity effect, such that the driver is prevented from reaching fixation owing to self-poisoning, which is well-known from others models of meiotic drive (*e.g.* Lewontin and Dunn 1960; Curtsinger and Feldman 1980; Holman *et al.* 2015). When a polymorphic equilibrium is possible, it is also stable because, when substituting this equilibrium ($\widehat{f_D}$) into the expression for the change in drive allele frequency (eqn. S1), such deviations from the equilibrium allele frequency are counteracted by its multiplied effect of $(-1 + 2h)s$; as a recessive toxicity effect means that h must be small, an increase in drive allele frequency from this equilibrium has a negative effect on the change in drive allele frequency, and a decrease in drive allele frequency has a positive effect. Therefore, if a drive allele can invade then it is expected either to spread to fixation or to reach a stable equilibrium against the nondrive allele when there is a large but recessive toxicity effect.

As noted above, in this scenario we explicitly assume that the reductive division that separates homologous chromosomes produces two homozygous sister cells. However, the presence of crossovers

in heterozygotes can mean that the two sister chromatids on the same chromosome contain different alleles, which can render the sister cells heterozygous at the drive locus. We do not include a formal quantitative analysis of the impact of crossovers on evolution in this system since their influence is obvious based on a qualitative consideration, which is analysed in the Appendix B and summarised here. Crossing-over can potentially reduce the strength of selection favouring a drive allele (D) if it gains its advantage at the sister cells stage, which would make it a meso-meiotic killer. This is because a meso-meiotic killer gains its transmission advantage in the sister cells containing the driver allele over the sister cells that contain the nondrive allele (d). However, a crossover could result in both sister cells containing one copy of the drive allele and one copy of the nondriver allele on sister chromatids (so they are both effectively Dd), which eliminates the ability of the driver to have a transmission advantage (since all sister cells contain both alleles). Therefore, crossover events reduce the advantage to a driver, and hence they should restrict the conditions that favour killer meiotic drivers when their effect is meso-meiotic. This leads to a well-known prediction that a meiotic driver is more robust in the absence of crossing-over, which is more likely when the drive locus is closer to the centromere or is in a region, such as an inversion, that shows restricted recombination (Pardo-Manuel De Villena & Sapienza, 2001; Malik & Henikoff, 2002; Burt & Trivers, 2008; Haig, 2010).

Further details of intralocus suppression by a resistant allele

When a resistant allele (δ) competes against the other alleles at that locus (the drive D and nondrive d alleles), we can again derive its fitness (Table S1) and calculate the change in the frequency of the drive allele from its covariance with relative fitness:

$$\Delta f_D = f_D[f_d e(1-hs) - (f_d + f_\delta)s(f_D + h(1-2f_D))]/\bar{w} \quad \text{(S4)}$$

Herein, a salient feature is that the drive allele is favoured against the nondrive allele (d) but disfavoured against the resistant allele (δ). Under this condition, there is necessarily only one stable equilibrium for this system of alleles: the fixation of the resistant allele. This is most apparent from recognising that the fitness of the resistant allele is always greater than the fitness of the nondrive allele because it does not suffer a transmission disadvantage, though a cost of resistance could mean that, in the absence of the drive allele, the resistant allele might be less fit than the nondrive allele (because it pays a cost for being resistant, but resistance has no benefit). The fitness of the resistant allele is also always greater than the fitness of the drive allele in the absence of the nondrive allele because it suffers a dominance-reduced toxicity cost (Table S1; given that h ranges between 0 and 1) and so the resistant allele is always favoured by selection. This is true even if there is a cost for being resistant, since that cost is presumably also paid by the drive allele (since it has to have resistance to the poison), and hence it does not create a difference in the fitness of the resistant and drive alleles. Hence, in this scenario, the drive allele is expected to rapidly spread through a population of nondrive alleles but, if a resistant allele were then to arise by mutation, it would rapidly spread leading to the drive allele's extinction (after which it could potentially be lost if it pays some cost for being resistant). Additionally, this result need not assume an absence of crossing-over, because crossovers would not impact the fitness of cells containing the resistant allele (since its success does not depend on different sister cells having different genotypes). As such, a drive allele is always susceptible to intralocus suppression by a resistant allele.

Assumptions about interlocus modifiers

A meiotic driver could be suppressed by a modifier elsewhere in the genome, which potentially offers a faster evolutionary route to resistance than intralocus suppression because the rest of the genome is simply a larger mutational target than a single locus. A modifier could potentially suppress the drive allele through numerous different pathways, but the main text outlined several simplifying restrictions to focus the analysis. In keeping with previous suggestions (*e.g.* Hurst *et al.* 1996) we only consider cost-free modifiers. Other studies have demonstrated that modifiers are only favoured if they have a sufficiently low cost (*e.g.* Scott and West 2019) and we do not expect the cost to interact with

the different drive mechanisms in an interesting way. A cost would simply increase the invasion criterion, decrease the equilibrium frequency and slow the rate of evolutionary change of the modifier allele. We also assume that a modifier can only influence expression of a drive allele when they are in the same cell. Modifiers that produce a diffusible regulator could potentially modulate expression of drive alleles in other interacting cells, but this presumably generates an arms race over the exogeneous and endogenous factors in the regulation of the drive allele, which is outside the scope of our analysis (but would presumably be won by the endogenous factors that are considered here). Finally, we only consider modifiers that completely block poison and/or antidote production. Although a modifier could potentially alter dosage, our analysis based on qualitatively different modifiers captures the fundamental evolutionary properties of the system without the added complexity introduced by the range of possible dosage effects.

Linkage disequilibrium, recombination and crossing-over

The main text focuses on results for the scenario of linkage equilibrium with free recombination. However, because the strength of selection on the drive or modifier allele can potentially be relatively strong, it can potentially generate linkage disequilibrium (LD) when it causes epistatic selection. Restricted recombination can also potentially have an impact on the evolution of this system. Therefore, to consider these potential effects we include an analysis of LD and recombination in Appendix B, which is extensive and so is briefly summarise here. We separate considerations of LD and recombination because, while LD changes the frequency of haplotype interactions in the population it does not change the frequency of haplotype interactions within an individual. In contrast, while recombination can influence evolution via its role in eroding LD, it can have an independent impact by altering the pattern of interactions among haplotypes during meiosis. Most importantly, we show that LD never changes the direction of selection on the drive or modifier allele; instead, LD can influence the relative rate of evolution. It has a complicated pattern of effects across different scenarios that most often leads to the deceleration of evolution, but it can also lead to acceleration in some scenarios and circumstances. A lack of free recombination only alters the pattern of haplotypic interactions in one

scenario: a post-meiotic killer with a fully suppressing modifier. Importantly, restricted recombination impacts selection in a predictable way because the scenario with no recombination is equivalent to the three-allele one-locus system of intralocus suppression, where the drive-modifier haplotypic combinations in the two-locus case have the same phenotypes as the individual alleles in the single-locus case (where $Dm=D$, $dM/dm=d$ and $DM=\delta$). As such, an intermediate recombination rate gives an intermediate set of results that fall in between the two extremes represented by the single-locus three-allele scenario and the two-locus case with free recombination. Hence, lower recombination rates further favour the invasion and spread of the modifier allele (as expected under the single-locus scenario). Therefore, whilst LD and a lack of free recombination can influence evolution, the basic results we focus on in the text from the simpler scenario of linkage equilibrium and free recombination capture the key properties that allow us to describe new predictions about the evolutionary robustness of killer meiotic drives based on the killing mechanism.

The main text also focuses on the scenario where there are no crossover events in production of sister cells. Similar to the single-locus case (see above), crossing-over only impacts meso-meiotic killers in the two-locus scenarios because it can change the genetic identities of the sister cells, which alters their interaction. As qualitatively analysed in Appendix B and very briefly summarised here, the impact of crossing-over is expected to shift the balance of selection toward disfavouring drive alleles and favouring modifier alleles. For this reason, we also expect the most successful and robust drivers (and modifiers) to be positioned closer to the centromere or otherwise in regions of low recombination (such as an inversion), where crossing-over will be rare and therefore will not reduce the strength of selection favouring the driver. This is consistent with a well-known prediction that a meiotic driver is more robust in the absence of crossing-over (Pardo-Manuel De Villena & Sapienza, 2001; Malik & Henikoff, 2002; Burt & Trivers, 2008; Haig, 2010).

References

Bravo Núñez, M.A., Nuckolls, N.L. & Zanders, S.E. 2018. Genetic Villains: Killer Meiotic Drivers. Trends Genet. 34: 424–433.

Burt, A. & Trivers, R. 2008. Genes in Conflict. Harvard University Press.

Curtsinger, J.W. & Feldman, M.W. 1980. Experimental and theoretical analysis of the “sex-ratio” polymorphism in Drosophila pseudoobscura. Genetics 94: 445–466.

Endo, T.R. 1990. Gametocidal chromosomes and their induction of chromosome mutations in wheat. Japanese J. Genet. 65: 135–152.

Haig, D. 2010. Games in Tetrads: Segregation, Recombination, and Meiotic Drive. Am. Nat. 176: 404–413.

Hamilton, W.D. 1967. Extraordinary Sex Ratios. Science (80-.). 156: 477–488.

Helleu, Q., GÉrard, P.R. & Montchamp-Moreau, C. 2015. Sex chromosome drive. Cold Spring Harb. Perspect. Biol. 7.

Holman, L., Price, T.A.R., Wedell, N. & Kokko, H. 2015. Coevolutionary dynamics of polyandry and sex-linked meiotic drive. Evolution (N. Y). 69: 709–720.

Hu, W., Jiang, Z.-D., Suo, F., Zheng, J.-X., He, W.-Z. & Du, L.-L. 2017. A large gene family in fission yeast encodes spore killers that subvert Mendel’s law. Elife 6: 1–19.

Hurst, L.D.., Atlan, A. & Bentsson, B.O. 1996. Genetic Conflicts. Q. Rev. Biol. 71: 317–364.

Jaenike, J. 2001. Sex Chromosome Meiotic Drive. Annu. Rev. Ecol. Syst. 32: 25–49.

Leigh, E.G. 1971. Adaptation and Diversity: natural history and the mathematics of evolution. Freeman, Cooper.

Lewontin, R.C. & Dunn, L.C. 1960. The evolutionary dynamics of a polymorphism in the house mouse. Genetics 45: 705–722.

Lindholm, A.K., Dyer, K.A., Firman, R.C., Fishman, L., Forstmeier, W., Holman, L., et al. 2016. The Ecology and Evolutionary Dynamics of Meiotic Drive. Trends Ecol. Evol. 31: 315–326.

Malik, H.S. & Henikoff, S. 2002. Conflict begets complexity: The evolution of centromeres. Curr. Opin. Genet. Dev. 12: 711–718.

Pardo-Manuel De Villena, F. & Sapienza, C. 2001. Nonrandom segregation during meiosis: The unfairness of females. Mamm. Genome 12: 331–339.

Price, T.A.R., Verspoor, R. & Wedell, N. 2019. Ancient gene drives: An evolutionary paradox. Proc. R. Soc. B Biol. Sci. 286.

Rick, C.M. 1966. Abortion of male and female gametes in the tomato determined by allelic interaction. Genetics 53: 85–96.

Scott, T.W. & West, S.A. 2019. Adaptation is maintained by the parliament of genes. Nat. Commun. 10: 1–13. Springer US.

Turner, B.C. & Perkins, D.D. 1991. Meiotic drive in Neurospora and other fungi. Am. Nat. 137: 416–429.

Appendix B. The analysis of linkage disequilibrium, recombination, and crossovers

The pattern of selection on alleles in a killer meiotic drive system depends on the pattern of interactions among cells during gametogenesis. Therefore, selection will depend on the frequencies with which different gamete types interact, which can depend on the linkage disequilibrium (LD) across genotypes, the degree of recombination within genotypes, and the pattern of crossing-over among sister cells. To understand how LD and recombination impact the pattern of selection in the two locus system (a drive and modifier locus) we analyse their influence separately. When there is LD, the level of recombination between loci can play a role in evolution by modifying the extent of LD that exists in a population, but this impact of recombination is essentially captured in an analysis focused on the role of LD because recombination impacts evolution via its influence on LD (*e.g.* restricted recombination can enhance the importance of LD by slowing its erosion, but this effect is mediated by the level of LD). Because selection on a drive allele (and on a modifier allele) depends on the pattern of interactions among haplotypes (*i.e.* gamete types) within individuals, recombination can potentially influence selection by altering the pattern of interactions among haplotypes within individuals. This effect does not depend on the presence of LD since it influences the pattern of gametes produced by a given genotype, while LD determines the distribution of haplotypes across individuals (but not the pattern of gametes produced by a given genotype). Therefore, to understand the importance of LD we analyse how selection on a meiotic drive system builds LD and how the presence of LD influences selection on the drive allele, which depends on variation in the frequencies of haplotypes across individuals. Whereas to understand the importance of recombination we analyse how restricted recombination alters the pattern of interactions among haplotypes, which can thereby modify the pattern of selection favouring a drive and modifier allele. As the pattern of selection can differ across the scenarios of pre-, meso- and post-meiotic killing, the roles of LD and restricted recombination are analysed for each scenario separately. By contrast, a crossover would alter the genetic structure of the interaction by modifying the combinations of drive and modifier alleles present in sister cells for meso-meiotic killing, which is explored separately at the end.

The role of linkage disequilibrium

To isolate the important of linkage disequilibrium (LD) we analyse each scenario to understand how that scenario builds LD and how the existence of LD influences evolution. We denote the frequencies of the four haplotypes as $H_{DM} = f_D f_M + L$, $H_{Dm} = f_D f_m - L, H_{dM} = f_d f_M - L$, and $H_{dm} = f_d f_m + L$ (Lewontin, 1974), where the parameter L gives a deviation from the random assortment of alleles into genotypes (note that, while LD is typically denoted by a *D* or *d*, we avoid this convention here by using L to clearly differentiate LD from the use of those labels as the allelic identifiers at the drive locus):

$$L = H_{DM} H_{dm} - H_{Dm} H_{dM} \quad \text{(S1)}$$

As such, a positive value indicates that haplotypes that contain the drive and modifier alleles together (and there, the nondrive and nonmodifier alleles together) are more common than expected from the allele frequencies. Because even strong selection tends to build relatively low levels of LD, we simplify our analysis by assuming that terms on the order L^2 or above are negligible and can be ignored.

LD can be built by natural selection due to additive and epistatic effects (Lewontin, 1974; Hastings, 1985). LD can arise from the additive effect because of the simultaneous selection of an allele at each locus, but this form of LD is uninteresting here because it does not favour the continuation of an association between alleles due to the formation of a co-adapted haplotype that would alter the model's results from the main text (that are derived assuming linkage equilibrium). LD can also arise from epistasis where some combinations of alleles have higher fitness than would be expected due to the additive contributions of each allele. The epistatic effect (*E*) is defined as (Hastings, 1985; Nagylaki, 1993):

$$E = (\omega_{DM} + \omega_{dm}) - (\omega_{Dm} + \omega_{dM}) \quad \text{(S2)}$$

From this standpoint, the expected relationship between LD (after one generation of selection from linkage equilibrium) and epistasis is:

$$L = f_D f_d f_M f_m E / \bar{\omega}^2 \quad \text{(S3)}$$

Thus, by calculating L and E in different scenarios, we can assess the extent to which natural selection builds LD because of co-adaptation in particular haplotypes. Logically, considering the five scenarios of gamete-killing gene drive, it is the epistatic effect that is critical to describing whether or not LD would change the model's results in the main text because the modifier allele has the power to change the fitness effect of the drive allele (*i.e.* there is epistasis), which could potentially favour an association between alleles that changes the pattern of selection by making some haplotypes more common.

To explore how LD can alter the evolution of the drive system, we first calculate the fitness of the four haplotypes assuming linkage equilibrium (ω_{DM}, ω_{Dm}, ω_{dM}, ω_{dm}; see Table SA.1), which is used to calculate the epistatic effect (eqn. S2; see Table SA.2). For some types of meiotic killer, the epistatic effect has correspondence to the condition for the drive or modifier allele to be favoured by selection (Table SA.2). For all scenarios, especially those where there is no clear correspondence, numerical simulations are used to support statements about the sign and magnitude of the analytical expressions by calculating numerical outcomes over 10^7 random samples from a uniform distribution (between 0 and 1) across each of the model parameters (e, s, h, f_D, f_M). As well as providing a description, the resulting sign of the epistatic effect is explained with reference to the fitness of the four haplotypes, which usually reveals a straight-forward relationship. The impact of epistasis on selection can then be assessed by examining the expressions for the change in drive and modifier allele frequency that include LD. The full fitness expressions are complicated and so, to simplify the analysis of LD to

an informative level, we restrict our attention to describe the sign of LD that natural selection builds in a single generation from the starting condition where there is linkage equilibrium (sometimes described elsewhere as L_0). This restriction simplifies the analysis without any loss of generality (because it allows us to identify and understand the phenomena that build LD while simply avoiding the complicating impacts of secondary, higher-order processes that arise once there is existing LD). In some scenarios, the sign of LD is enough to identify the impact of LD on selection, but in other scenarios the maximum possible magnitude of LD based on the frequencies of alleles (L_{max} or L_{min} depending on the sign of LD) can be used to assess whether the presence of LD changes the direction of selection and/or the rate of evolution. Following this approach, each drive system with a type of meiotic killer and modifier suppression is taken in turn. LD will only play an appreciable role when the drive and modifier allele can invade. Within this restriction, there are multiple distinctive scenarios that can be separated on the basis of the direction of selection in the absence of LD and (sometimes within that) the sign of epistasis.

Table SA.1. The fitness of the four haplotypes of the combinations of drive/nondrive and modifier/nonmodifier alleles (ω_{DM}, ω_{Dm}, ω_{dM}, ω_{dm}) under different gamete-killing scenarios (see Tables 4, 5, 6). **A)** Pre-meiotic killing, wherein full or partial suppression by a modifier allele is equivalent. **B)** Post-meiotic killing, under the scenarios of a fully or partially suppressing modifier. **C)** Meso-meiotic killing, under the scenarios of a fully or partially suppressing modifier.

A)

Haplotype	Pre-meiotic; Full/Partial suppression
DM	1
Dm	$1 - f_m\left(f_D s + f_d\left(hs - e(1 - hs)\right)\right)$
dM	1
dm	$1 - f_D f_m\left(hs + e(1 - hs)\right)$

B)

Haplotype	Post-meiotic; Full suppression	Post-meiotic; Partial suppression
DM	$1 - f_m\left(hs + e(1 - hs)\right)$	$1 - f_m\left(hs - f_d e(1 - hs)\right)$
Dm	$1 - f_D f_m s - (1 - f_D f_m)\left(hs - e(1 - hs)\right) + 2 f_d f_M e(1 - hs)$	$1 - f_D(f_M h + f_m)s$ $- f_d\left(hs - e(1 - s)\right)$
dM	$1 - f_D f_m\left(hs + e(1 - hs)\right)$	$1 - f_D f_m\left(hs + e(1 - hs)\right)$
dm	$1 - f_D\left(hs + e(1 - hs)\right)$	$1 - f_D\left(hs + e(1 - hs)\right)$

B)

Haplotype	Meso-meiotic; Full suppression	Meso-meiotic; Partial suppression
DM	$1 - f_D f_m\left(hs + e(1 - hs)\right)$	$1 - f_D f_m hs$
Dm	$1 - f_D f_m s - (1 - f_D f_m)\left(hs - e(1 - hs)\right)$	$1 - f_D(f_M h + f_m)s$ $- f_d\left(hs - e(1 - s)\right)$
dM	$1 - f_D f_m\left(hs + e(1 - hs)\right)$	$1 - f_D f_m\left(hs + e(1 - hs)\right)$
dm	$1 - f_D f_m\left(hs + e(1 - hs)\right)$	$1 - f_D f_m\left(hs + e(1 - hs)\right)$

Table SA.2. Epistatic effect under different scenarios of gamete-killing, alongside the condition for the drive and modifier alleles to be favoured by selection. The correspondence between the epistatic effect and the condition for an allele to be favoured by selection is emphasised in blue.

**Scenario**	**Epistatic effect (E)**	**Drive allele favoured** ($\Delta f_D > 0$)	**Modifier allele favoured** ($\Delta f_M > 0$)
Pre-meiotic; Full/Partial suppression	$-f_m\big(e(1-hs)-s(f_D+h(f_d-f_D))\big)$	$e(1-hs)-s(f_D+h(f_d-f_D)) > 0$	$NA*$
Post-meiotic; Full suppression	$-\begin{pmatrix} f_d e(1-hs)+e(1+f_M)(1-hs) \\ -s(f_D f_m+h(f_M-f_D)) \end{pmatrix}$	$-f_d hs+e(1+f_M)(1-hs)$ $-s\big(f_D f_m+h(f_M-f_D)\big) > 0$	$f_M hs-e(1+f_d)(1-hs)$ $+s\big(f_D f_m+h(f_M-f_D)\big) < 0$
Post-meiotic; Partial suppression	$-\begin{pmatrix} f_M e(1-hs) \\ -s\big(f_D f_m+h(f_M-f_D)\big) \end{pmatrix}$	$-f_d hs+e(1-hs)+f_M e(1-hs)$ $-s\big(f_D f_m+h(f_M-f_D+f_d)\big) > 0$	$NA*$
Meso-meiotic; Full suppression	$-\big(e(1-hs)-s(f_D f_m(1-2h)+h)\big)$	$e(1-hs)-s(f_D f_m(1-2h)+h) > 0$	$e(1-hs)-s\big(f_D f_m(1-2h)+h\big) < 0$
Meso-meiotic; Partial suppression	$-\big(f_d e(1-hs)-s(f_D f_m(1-2h)+h)\big)$	$e(1-hs)-s(f_D f_m(1-2h)+h) > 0$	$f_d e(1-hs)-s(f_D f_m(1-2h)+h) < 0$

* The modifier allele always has higher fitness than the non-modifier allele, given that $0 \le s \le 1$, $0 \le h \le 1$ and $0 \le e \le 1$.

Pre-meiotic killers

The pattern of epistasis generated by a pre-meiotic killer is equivalent but with opposite sign to the pattern of selection that favours a meiotic driver. For pre-meiotic killing, whilst there is no evolutionary distinction in terms of fitness consequences between full and partial suppression, a modifier can always invade and is always favoured by selection in the absence of LD. Therefore, for the parameter space where both the drive and modifier allele can invade (which is where LD can have a non-trivial impact), there are two scenarios where the presence of LD can impact the selection on the drive and modifier alleles.

In the first scenario, wherein the drive allele is not favoured by selection and the modifier allele is favoured by selection in the absence of LD, the epistatic term is always positive (Table SA.2). Positive epistasis arises from the way that the *Dm* haplotype disadvantages both the *Dm* and *dm* haplotypes by producing the poison, which results in selection favouring the *DM* and *dM* haplotypes that never encounter the poison. This builds positive LD because of the relative disadvantage to the *Dm* haplotype over the *dm* haplotype. The impact of LD on the selection on the drive allele can be examined using the expression for the change in allele frequency. This expression can be broken into an LD independent part, which describes the change in the drive allele's frequency in the absence of LD and an LD dependent part, which is given here to the first-order approximation:

$$\begin{aligned}\Delta f_D \bar{\omega} = {} & f_D f_d f_m^2 [e(1-hs) - s(f_D + h(f_d - f_D))] \\ & + L f_m [e(f_D - f_d)(1-hs) + s(h + 2 f_D f_d (1-2h))]\end{aligned} \tag{S4}$$

Accordingly, with positive LD ($L > 0$), the whole LD dependent part (*i.e.* the term including L on the second line of the equation) is always positive, which is the opposite sign to the LD independent part (given that the drive allele is not favoured by selection in the absence of LD). Nonetheless, as confirmed by numerical simulation, the sign of the change in drive allele frequency (Δf_D) is always negative for

the maximum value of positive LD ($L = \min(f_D f_m, f_d f_M)$), and so epistasis cannot change the direction of selection on the drive allele (Table SA.3). As such, LD leads to a deceleration in the rate of change in allele frequency of the drive allele, but does not change the fact that selection always disfavours the drive allele in this scenario. The impact of LD on the selection on the modifier allele can be examined in a similar way:

$$\Delta f_M \bar{\omega} = f_D f_M f_m^2 s[f_D + 2h f_d] \\ +2L f_m f_M s[f_D + h(f_d - f_D)] \tag{S5}$$

With positive LD ($L > 0$), the whole LD dependent part (including L) is always positive. Consequently, as confirmed by numerical simulation, the sign of the change in modifier allele frequency (Δf_M) is always positive for the maximum value of positive LD ($L = \min(f_D f_m, f_d f_M)$), and so epistasis cannot change the direction of selection on the modifier allele. Instead, LD leads to an acceleration in the rate of change in frequency of the modifier allele. Therefore, whilst LD alters the rate of evolution of the drive and modifier alleles in this scenario, it does not change the evolutionary outcome.

In the second scenario, wherein the drive allele is favoured by selection and the modifier allele is favoured by selection in the absence of LD, the epistatic term is negative (Table SA.2). Negative epistasis occurs because the drive allele's (*D*) fitness advantage arises from the transmission advantage that the *Dm* haplotype has over the *dm* haplotype in the *Ddmm g*enotype (see Table 4). As a result, the *Dm* haplotype increases in relative frequency while the *dm* decreases, leading to negative LD. However, whilst the transmission advantage to the drive allele is entirely from the *Dm* haplotype and comes from being in a genotype with the *dm* haplotype, the *Dm* haplotype has a disadvantage in an individual with two *Dm* haplotypes with the *DDmm* genotype (see Table 4). Hence, there are opposing effects at play, which ultimately depend on how negative LD affects the relative frequency of the *Dm* haplotype compared to the *dm* haplotype and the relative frequency of the *Dm* haplotype compared to the *DM* haplotype. When the *Dm* haplotype is relatively rare compared to the *dm* haplotype, negative LD would

increase the relative frequency of the *Ddmm g*enotype wherein the *Dm* haplotypes gets its advantage, which accelerates the rate of change in the frequency of the drive allele (Δf_D); but, as the *Dm* haplotype becomes more common, negative LD would increase the relative frequency of the *DDmm* genotype where the *Dm* haplotypes gets a disadvantage and so decelerates the rate of change in the frequency of the drive allele (Δf_D). This explanation can be confirmed with the examination of the expression for the change in the drive allele's frequency (eqn. S4), where the LD dependent part has a sign-altering expression ($f_D - f_d$), which depends on which allele, the drive or nondrive allele, is more common. Although the trend is complicated by the relative magnitude of the transmission and toxicity effect (e, s), the LD dependent part is more likely to be positive with higher drive allele frequencies and more likely to be negative with lower drive allele frequencies; LD more often (66.7% of simulated runs) leads to a deceleration in the rate of the change in the frequency of the drive allele. Regardless, as confirmed by numerical simulation, the sign of the change in drive allele frequency (Δf_D) is always positive for the maximum value of negative LD ($L = \max\left(-f_D f_M, -f_d f_m\right)$), and so epistasis, which creates the LD, cannot change the direction of selection on the drive allele. A similar outcome also occurs for the impact of LD on the change in frequency of the modifier allele. This scenario has negative epistasis because the drive allele is favoured (for reasons outlined above) and consequently the LD dependent part of the expression for the change in modifier allele frequency (see eqn. S5) is always negative because there is always negative LD (as $L < 0$). Nonetheless, as confirmed by numerical simulation, the modifier allele is always favoured by selection, and so LD does not change the direction of selection – although it does decelerate the rate of change in the frequency of the modifier allele. Consequently, like the alternative possibility, whilst LD alters the rate of evolution it does not change the outcome. Therefore, overall, LD never changes the direction of evolution with a pre-meiotic killer, although it can both accelerate the rate of change in the drive allele frequency when it is rare and decelerate the rate of change when it is common. Likewise, it will accelerate the rate of change in the frequency of the modifier allele when drive allele is disfavoured by selection and decelerate the rate of change when the drive allele is favoured by selection.

Meso-meiotic killers with full suppressors

With a fully supressing modifier, the pattern of epistasis generated by a meso-meiotic killer is equivalent but with opposite sign to the pattern of selection that favours a meiotic driver. Like the case of pre-meiotic killing, the drive and the modifier allele are never favoured by selection simultaneously in the absence of LD. Therefore, for the parameter space where both the drive and modifier allele can invade (which is where LD can have a non-trivial impact), there are two scenarios where the presence of LD can impact selection on the drive and modifier alleles.

In the first scenario, wherein the drive allele is not favoured by selection and the modifier allele is favoured by selection in the absence of LD, the epistatic term is always positive (Table SA.2). As in the same scenario for pre-meiotic killers, positive epistasis arises from the way that the *Dm* haplotype disadvantages both the *Dm* and *dm* haplotypes by producing the poison, which results in selection favouring the *DM* and *dM* haplotypes that never encounter the poison. This builds positive LD because of the relative disadvantage to the *Dm* haplotype over the *dm* haplotype. As before, the impact of LD on selection on the drive allele can be examined by separating the expression for the change in allele frequency into LD independent and dependent parts (given to the first-order approximation):

$$\Delta f_D \bar{\omega} = f_D f_d f_m[e(1-hs) - s(f_D f_m(1-2h)+h)]$$
$$+\tfrac{1}{2}L[e(1-hs)(f_D - f_d) + s(4f_D f_d f_m(1-2h)+h)] \quad \text{(S6)}$$

With positive LD ($L > 0$), the whole LD dependent part (including L) is always positive, which is the opposite sign of the LD independent part (given that the drive allele is not favoured by selection in the absence of LD). Nonetheless, as confirmed by numerical simulation, the sign of the change in drive allele frequency (Δf_D) is always negative for the maximum value of positive LD ($L = \min(f_D f_m, f_d f_M)$), and so epistasis cannot change the direction of selection on the drive allele (Table

SA.3). As such, LD leads to a deceleration in the rate of the change in the frequency of the drive allele. This result is the same as for this scenario with the pre-meiotic killer. The impact of LD on the evolutionary change in the frequency of the modifier allele can be examined in a similar way:

$$\Delta f_M \bar{\omega} = f_D f_M f_m[-e(1-hs) + s(f_D f_m(1-2h) + h)] + \tfrac{1}{2}L[e(1-hs)(f_M - f_m) - s(4f_M f_m f_D(1-2h) + h) \tag{S7}$$

With positive LD ($L > 0$), the whole LD dependent part is always negative, which is the opposite sign of the LD independent part and different from this scenario with the pre-meiotic killer. Nonetheless, as confirmed by numerical simulation, the sign of the change in modifier allele frequency (Δf_M) is always positive for the maximum value of positive LD ($L = \min(f_D f_m, f_d f_M)$), and so epistasis cannot change the direction of selection on the modifier allele. Instead, LD leads to a deceleration in the rate of the change in the frequency of the modifier allele. Therefore, whilst LD alters the rate of change in the frequency of drive and modifier alleles in this scenario, it does not change the evolutionary outcome.

In the second scenario, wherein the drive allele is favoured by selection and the modifier allele is not favoured by selection in the absence of LD, the epistatic term is negative (Table SA.2) because the fitness advantage of the drive allele (*D*) comes from the transmission advantage that the *Dm* haplotype has over the other haplotypes in various *g*enotypes (see Table 6A), leading to negative LD. However, whilst the transmission advantage to the drive allele is entirely from the *Dm* haplotype, the *Dm* haplotype has a disadvantage in an individual with two *Dm* haplotypes with the *DDmm* genotype (see Table 6A). So, as in the same scenario with a pre-meiotic killer, there are opposing effects at play, such that when the *Dm* haplotype is relatively rare compared to the *dm* haplotype, negative LD would increase the relative frequency of the *g*enotypes where the *Dm* haplotypes gets its advantage; but, as the *Dm* haplotype becomes more common, negative LD would increase the relative frequency of the *DDmm* genotype where the *Dm* haplotypes gets a disadvantage. The impact of LD on selection on the drive

allele can then be examined using the expression for the change in allele frequency separated into LD independent and dependent parts (eqn. S6). With negative LD ($L < 0$), the whole LD dependent part is almost always positive (99.4% of simulated runs). Nonetheless, as confirmed by numerical simulation, the sign of the change in drive allele frequency (Δf_D) is always negative for the maximum value of negative LD ($L = \max(-f_D f_M, -f_d f_m)$), and so epistasis cannot change the direction of selection on the drive allele. As such, LD almost always (99.4%) leads to a deceleration in the rate of the change in the frequency of the drive allele. The impact of LD on selection on the modifier allele can be examined in a similar way, by decomposing the expression for the change in allele frequency into LD independent and dependent parts (eqn. S7). The sign of the LD dependent part depends on a complex interplay of a number of factors, including whether the modifier or nonmodifier allele is more common (captured by the term $f_M - f_m$) and the sign of $e(1 - hs)$. The presence of LD most often (83.5%) decelerates the rate of change in the frequency of the modifier allele, but when the modifier has higher allele frequency ($f_M > 0.8$) it can accelerate the rate of change. However, as confirmed by numerical simulation, this cannot change the direction of selection even under maximum negative LD. Therefore, for meso-meiotic killers with full suppressors, LD never changes the direction of evolution, but it can lead to a complicated pattern of accelerating and decelerating rates of evolutionary change in the frequency of the drive and modifier alleles (see Table SA.3 for a summary).

Meso-meiotic killers with partial suppressors

With a partially suppressing modifier allele, the pattern of epistasis generated by a meso-meiotic killer is equivalent but with opposite sign to the pattern of selection that favours the modifier. Given that both the drive and modifier alleles can invade (which is where LD can have a non-trivial impact), numerical simulation confirms that the drive and the modifier allele can never both be disfavoured by selection. Consequently, there are three scenarios where the presence of LD can impact the selection on the drive and modifier alleles.

In the first scenario, wherein the drive allele is not favoured by selection and the modifier allele is favoured by selection in the absence of LD, the epistatic term is positive (Table SA.2). As in the same scenario for pre-meiotic killers and meso-meiotic killers with full suppressors, positive epistasis arises from the way that the *Dm* haplotype disadvantages both the *Dm* and *dm* haplotypes by producing the poison, which results in selection favouring the *DM* and *dM* haplotypes that never encounter the poison. This builds positive LD because of the relative disadvantage to the *Dm* haplotype over the *dm* haplotype. The impact of LD on selection on the drive allele can be examined using the expression for the change in allele frequency separated into LD independent and dependent parts, which is the same for the scenario with full suppressors (eqn. 6). With positive LD ($L > 0$), the whole LD dependent part (including L) is always positive, which is the opposite sign of the LD independent part. Yet, as confirmed by numerical simulation, the sign of the change in drive allele frequency (Δf_D) is always negative for the maximum value of positive LD ($L = \min(f_D f_m, f_d f_M)$), and so epistasis cannot change the direction of selection on the drive allele (Table SA.3). As such, LD leads to a deceleration in the rate of the change in the frequency of the drive allele. The impact on the selection on the modifier allele can be analysed in a similar way, but the expression for the change in allele frequency differs from the case with a meso-meiotic killer with full suppressor:

$$\Delta f_M \bar{w} = f_D f_M f_m[-e f_d(1 - hs) + s(f_D f_m(1 - 2h) + h)]$$
$$+\frac{1}{2}L[e(1 - hs)(f_M - f_m)(f_d - f_D) - s(4 f_M f_m f_D(1 - 2h) + h) \qquad \text{(S8)}$$

With positive LD ($L > 0$), the whole LD dependent part is almost always negative (95.2% of simulated runs). The LD dependent part tends to be negative but can be positive with the right combination of allele frequencies, which requires the drive allele to be more common ($f_D > 0.5$) and the modifier allele to be rare ($f_M < 0.15$). Whilst this can lead to the acceleration in the rate of change in the frequency of

the modifier allele when it is rarer, almost always (95.2%) it leads to deceleration in the rate of change in the frequency of the modifier allele.

In the second scenario, wherein the drive allele is favoured by selection and the modifier allele is not favoured by selection in the absence of LD, the epistatic term is negative (Table SA.2). As in the same scenario with a full suppressor, negative epistasis arises because selection favours the drive allele (D) for the transmission advantage that the Dm haplotype has over the other haplotypes in various genotypes (see Table SA3), leading to negative LD. But there are opposing effects at play, such that when the Dm haplotype is relatively rare compared to the dm haplotype, negative LD would increase the relative frequency of the genotypes where the Dm haplotypes gets its advantage; but, as the Dm haplotype becomes more common, negative LD would increase the relative frequency of the $DDmm$ genotype where the Dm haplotypes gets a disadvantage. The impact of LD on selection on the drive allele can then be examined by separating the expression for the change in allele frequency into LD independent and dependent parts (eqn. S6). With negative LD ($L < 0$), the whole LD dependent part is almost always positive (98.4% of simulated runs). Nonetheless, as confirmed by numerical simulation, the sign of the change in drive allele frequency (Δf_D) is always negative for the maximum value of negative LD ($L = \max(-f_D f_M, -f_d f_m)$), and so epistasis cannot change the direction of selection on the drive allele. As such, LD almost always (98.4%) leads to a deceleration in the rate of the change in the frequency of the drive allele. The impact of LD on selection on the modifier allele can be examined in a similar way by dividing the expression for the change in allele frequency into LD independent and dependent parts (eqn. S8). The sign of the LD dependent part depends on a complex interplay of components, in particular the joint relative frequency of the alternative alleles at each locus (captured by the $(f_M - f_m)(f_d - f_D)$ term) and the sign of $e(1 - hs)$. However, the LD dependent term almost always (97.4%) decelerates the rate of change in the frequency of the modifier allele, except when the modifier has higher allele frequency ($f_M > 0.8$) where it can accelerate the rate of change in the frequency of the modifier allele. However, as confirmed by numerical simulation, this effect cannot change the direction of selection under maximum negative LD.

In the third and final scenario, which has not appeared previously, where the drive allele is favoured by selection and the modifier allele is favoured by selection in the absence of LD, the epistatic term is positive (Table SA.2). When selection favours the drive allele, its advantage comes from the transmission effect that the *Dm* haplotype has over the other *dm* and *dM* haplotypes (see Table 6A). When selection favours the modifier allele, its advantage comes from removing the transmission advantage (and against the *dm* haplotype also its toxicity effect) in the *DM* haplotype. Positive epistasis arises as a simple consequence of the fact that the modifier allele has a greater advantage in the *DM* haplotype than the drive allele has in the *Dm* haplotype. The impact of LD on selection on the drive allele can be examined using the expression for the change in allele frequency separated into LD independent and dependent parts, which is the same for the scenario with full suppressors (eqn. 6). With positive LD ($L > 0$), the whole LD dependent part is always positive, which is the same sign of the LD independent part. Consequently, as confirmed by numerical simulation, the sign of the change in drive allele frequency (Δf_D) is always positive even with the maximum value of positive LD ($L = \min(f_D f_m, f_d f_M)$), and so epistasis cannot change the direction of selection on the drive allele (Table SA.3). As such, LD leads to an acceleration in the rate of change in the frequency of the drive allele. The impact on selection on the modifier allele can be analysed in a similar way, using its own expression for the change in modifier allele frequency separated into LD independent and dependent parts (eqn. S8). With positive LD ($L > 0$), the sign of the whole LD dependent part depends on a number of factors, including the joint relative frequencies of the alleles at the two loci (captured by the product of $f_D - f_d$ and $f_M - f_m$). Whilst LD can lead to the acceleration of the rate of change in the frequency of the modifier allele when it is rarer, it almost always (87.4% of runs) leads to deceleration in the rate of change in the frequency of the modifier allele. Therefore, for meso-meiotic killers with partial suppressors, LD never changes the direction of selection, but (just like with full suppressors) it can lead to a complicated pattern of accelerating and decelerating rates of evolution at the drive and modifier loci (see Table SA.3 for a summary).

Post-meiotic killers with full suppressors

With a modifier for full suppression, the pattern of epistasis generated by a post-meiotic killer is not directly related to the conditions where a drive or modifier allele are favoured by selection. Consequently, the pattern of epistasis generated by selection is more complicated than in the previous cases because all four possible selection scenarios (in terms of the direction of selection on the drive and modifier alleles) need to be considered individually. Nonetheless, when the combinations of directions of selection are similar to the previously considered types of killer and suppressor, we see clear parallels that indicate uniformities in how selection operates.

In the first scenario (which has not appeared in the previous cases), wherein both the drive and modifier alleles are not favoured by selection in the absence of LD, epistasis is always negative (Table SA.2). This pattern of epistasis arises because of the advantage that the *Dm* haplotype has against the other haplotypes (see Table 5A), which builds negative LD that disfavours the drive allele because of its disadvantage in the *DM* haplotype. Following the same method as before, the impact of LD on selection on the drive allele can be examined by separating the expression for the change in allele frequency into LD independent and dependent parts (given to the first-order approximation):

$$\begin{aligned}\Delta f_D \bar{\omega} = {} & f_D f_d f_m\big[e(1+f_M)(1-hs) - s\big(f_D(f_m - 2h) + h(1+f_M)\big)\big] \\ & +L[ef_M(1-hs)(f_D - f_d) + sf_d(f_M h + 2f_D(f_m - h)]\end{aligned} \tag{S9}$$

With negative LD ($L < 0$), the whole LD dependent part (including L) is always negative. Therefore, as confirmed by numerical simulation, the sign of the change in drive allele frequency (Δf_D) is always negative even with the maximum value of negative LD ($L = \max\,(-f_D f_M, -f_d f_m)$). Hence, epistasis cannot change the direction of selection on the drive allele, but it does accelerate the rate of change in the frequency of the drive allele. Similarly, when examining the impact of LD on the selection on the modifier allele we can separate the change in allele frequency into LD independent and dependent parts:

$$\Delta f_M \bar{\omega} = -f_D f_M f_m[e(1+f_d)(1-hs) - s(f_D(f_m - h) + 2hf_M)]$$
$$+L[ef_d(1-hs)(f_M - f_m) - s(f_d h + 2f_M f_m(f_D - h)] \quad \text{(S10)}$$

With negative LD ($L < 0$), the whole LD dependent part is always positive. Although this counteracts the direction of selection on the LD independent part, simulations confirm that the sign of the change in modifier allele frequency (Δf_M) is always negative with the maximum value of negative LD ($L = \max(-f_D f_M, -f_d f_m)$). So, as for the drive allele, epistasis cannot change the direction of selection on the modifier allele, but (unlike the drive allele) it does decelerate the rate of change in the frequency of the modifier allele.

In the second scenario, wherein the drive allele is favoured by selection and the modifier allele is not favoured by selection in the absence of LD, the epistatic term is negative (Table SA.2). This is because selection favours the drive allele (*D*) for the transmission advantage that the *Dm* haplotype has over the other haplotypes in various *g*enotypes (see Table 5A), leading to negative LD. However, whilst the transmission advantage to the drive allele is entirely from the *Dm* haplotype, the *Dm* haplotype has a disadvantage in an individual with two *Dm* haplotypes with the *DDmm* genotype (see Table 5A). So, like the same scenario with a pre- or meso-meiotic killer, there are opposing effects at play, such that when the *Dm* haplotype is relatively rare compared to the *dm* haplotype, negative LD would increase the relative frequency of the *g*enotypes where the *Dm* haplotypes gets its advantage; but, as the *Dm* haplotype becomes more common, negative LD would increase the relative frequency of the *DDmm* genotype where the *Dm* haplotypes gets a disadvantage. The impact of LD on selection on the drive allele can then be examined using the expression for the change in allele frequency separated into LD independent and dependent parts (eqn. S9). LD almost always (99.2%) leads to a deceleration in the rate of change in the frequency of the drive allele. However, as confirmed by numerical simulation, the sign of the change in drive allele frequency (Δf_D) is always negative for the maximum value of negative

LD ($L = \max(-f_D f_M, -f_d f_m)$), and so epistasis cannot change the direction of selection on the drive allele.

The impact of LD on the selection on the modifier allele can be examined in a similar way, using the expression for the change in allele frequency separated into LD independent and dependent parts (eqn. S10). The sign of the LD dependent part critically depends on the relative frequency of the modifier and nonmodifier ($i.e.\ f_M - f_m$) and the sign of $e(1 - hs)$. However, the LD dependent term is almost always negative (94.3%) whereupon it decelerates the rate of change in the frequency of the modifier allele, except when the modifier has higher allele frequency ($f_M > 0.85$) where it can accelerate the rate of change in the frequency of the modifier allele. As confirmed by numerical simulation, this effect cannot change the direction of selection under maximum negative LD.

In the third scenario, wherein the drive allele is not favoured by selection and the modifier allele is favoured by selection in the absence of LD, the epistatic term can be positive (76.6% of simulated runs) or negative (23.4%; Table SA.2). We explore these two regions of parameter space separately. When there is positive epistasis, the explanation is similar to the previous scenarios because of the way that the *Dm* haplotype disadvantages both the *Dm* and *dm* haplotypes by producing the poison, resulting in positive epistasis. With analysis of the change in drive allele frequency (eqn. S9) with positive LD ($L > 0$), the LD dependent part counteracts the direction of selection on the LD independent part, resulting in a deceleration in the rate of change in the frequency of the drive allele. However, as confirmed by numerical simulation, the sign of the change in drive allele frequency (Δf_D) is always negative for the maximum value of positive LD ($L = \min(f_D f_m, f_d f_M)$), and so epistasis cannot change the direction of selection on the drive allele (Table SA.3). With positive epistasis and LD ($L > 0$), the change in the modifier allele frequency (eqn. S10) shows that the LD dependent part is in the opposite direction of selection as the LD independent part and hence decelerates the rate of change in the frequency of the modifier allele. As expected (given that LD decelerates evolution), numerical simulation using the maximum value of positive LD confirms that the sign of the change in modifier allele frequency (Δf_D) is always positive, which means that epistasis cannot change the direction of selection on the modifier.

When there is negative epistasis, the balance of selection is shifted against the drive allele to further disfavour the *DM* haplotype in comparison to the *dM* haplotype because the *DM* haplotype suffers an additional transmission disadvantage against the *Dm* haplotype. Therefore, the LD dependent and independent components of the change in drive allele frequency (eqn. S9) with negative LD ($L < 0$) go in the same direction, which accelerates the rate of change in the frequency of the drive allele. We see a similar result for the modifier allele (eqn. S10), where the LD dependent and independent parts share the same sign and hence LD accelerates the rate of evolution. Hence, as confirmed by numerical simulation with the maximum value of negative LD ($L = \max(-f_D f_M, -f_d f_m)$), the sign of the change in drive allele frequency (Δf_D) is always negative, while the sign of change in modifier allele frequency (Δf_M) is always positive, which means that epistasis cannot change the direction of selection on either the drive or modifier.

In the fourth scenario, wherein the drive allele is favoured by selection and the modifier allele is favoured by selection in the absence of LD, the epistatic term is negative (Table SA.2), which is similar to this scenario with the pre-meiotic killer. Negative epistasis occurs because selection favours the drive allele (*D*) for the transmission advantage that the *Dm* haplotype has over the other haplotypes (see Table 5A). With analysis of the change in drive allele frequency (eqn. S9), the LD dependent part (excluding L) is always positive, which is unlike this scenario with a pre-meiotic killer. Consequently, with negative LD ($L < 0$), the LD dependent part is in the opposite direction as the LD independent part, which decelerates the rate of change in the frequency of the drive allele. Yet, as confirmed by numerical simulation with the maximum value of negative LD ($L = \max(-f_D f_M, -f_d f_m)$), the sign of the change in drive allele frequency (Δf_D) is always positive. Therefore, epistasis cannot change the direction of selection on the drive allele (Table SA.3). In contrast, for the change in modifier allele frequency (eqn. S10) with negative LD ($L < 0$), the LD dependent part is in the same direction as the LD independent part, which accelerates the rate of evolution at the modifier locus. Hence, as confirmed by numerical simulation with the maximum value of negative LD ($L = \max(-f_D f_M, -f_d f_m)$), the sign of the change in modifier allele frequency (Δf_D) is always positive. Therefore, epistasis cannot change the direction of selection on the modifier allele (Table SA.3).

Therefore, overall for post-meiotic killers with full suppressors, there are a diversity of possible impacts of LD on the rate of evolutionary change in frequencies of the drive and modifier alleles (see Table SA.3 for a summary). However, while it can affect the rate of evolution, critically we see that the presence of LD can never change the direction of evolution.

Post-meiotic killers with partial suppressors

With a modifier for partial suppression, the pattern of epistasis generated by a post-meiotic killer is not directly associated with the conditions where a drive or modifier allele are favoured by selection. Although this is similar to the scenario with post-meiotic killers and full suppressors, selection in this scenario does not lead to the same diversity of possible outcomes because a modifier is always favoured by selection. In this way, like the similar case of pre-meiotic killers, there are just two scenarios based on the direction of selection on the drive allele (given that both alleles can invade).

In the first scenario, wherein the drive allele is not favoured by selection and the modifier allele is favoured by selection in the absence of LD, the epistatic term is always positive (Table SA.2). This is much like the similar pre- or meso-meiotic killer scenarios and so the explanation is also similarly due to the way that the *Dm* haplotype disadvantages both the *Dm* and *dm* haplotypes by producing the poison, resulting in positive epistasis. For the change in drive allele frequency (eqn. S9) with positive LD ($L > 0$), the LD dependent part (including L) counteracts the direction of selection on the LD independent part, which leads to a deceleration in the rate of change in the frequency of the drive allele. Yet, as confirmed by numerical simulation, the sign of the change in drive allele frequency (Δf_D) is always negative for the maximum value of positive LD ($L = \min(f_D f_m, f_d f_M)$), and so epistasis cannot change the direction of selection on the drive allele (Table SA.3).

The impact on the selection on the modifier allele can be analysed in a similar way, but the expression for the change in allele frequency differs from the case with a meso-meiotic killer with full suppressor:

$$\Delta f_M \bar{\omega} = f_D f_M f_m s[f_D(f_m - h) + 2h f_M] \\ + Ls[2 f_M f_m (h - f_D) - f_d h] \quad \text{(S11)}$$

With positive LD ($L > 0$), the LD dependent part is also in the opposite direction of selection as the LD independent part and therefore leads to a deceleration in the rate of change in the frequency of the modifier allele. However, again, as confirmed by numerical simulation, the sign of the change in modifier allele frequency (Δf_M) is always positive for the maximum value of positive LD ($L = \min(f_D f_m, f_d f_M)$), and so epistasis cannot change the direction of selection on the drive allele (Table SA.3).

In the second scenario, wherein the drive allele is favoured by selection and the modifier allele is favoured by selection in the absence of LD, the epistatic term can be positive (16.7% of simulated runs) or negative (80.3%; Table SA.2), which is unlike the same scenario with pre- or meso-meiotic killers. We explore these two possible outcomes separately. In each case, the explanation depends on the balance of whichever haplotypes are more strongly favoured by selection: whether the *Dm* haplotype is more favoured because of its transmission advantage against other haplotypes or whether the *DM* haplotype is more favoured because of its does not pay a full toxicity cost (Table 5B) against the *Dm* haplotype. For the change in drive allele frequency (eqn. S9) with positive LD ($L > 0$), the LD dependent part is in the same direction of selection as the LD independent part and hence can accelerate the rate of evolution at the drive locus. However, as confirmed by numerical simulation with the maximum value of positive LD ($L = \min(f_D f_m, f_d f_M)$), the sign of the change in drive allele frequency (Δf_D) is always positive and hence epistasis cannot change the direction of selection on the drive allele (Table SA.3). In contrast, with positive epistasis and LD ($L > 0$), for the change in the modifier allele frequency (eqn. S11) the LD dependent part is in the opposite direction of selection as the LD independent part, and hence LD decelerates the rate of evolution at the modifier locus. Yet, as confirmed by numerical simulation with the maximum value of positive LD, the sign of the change in modifier

allele frequency (Δf_D) is always positive, which also means that epistasis cannot change the direction of selection on the modifier.

When there is negative epistasis, the LD dependent part of the change in drive allele frequency (eqn. S9) can accelerate (58.4% of runs) or decelerate the rate of change in the frequency of the drive allele. The negative LD dependent part (*i.e.* deceleration) only occurs with lower drive allele frequencies ($f_D < 0.5$). However, as confirmed by numerical simulation, the sign of the change in drive allele frequency (Δf_D) is always negative for the maximum value of negative LD ($L = \max(-f_D f_M, -f_d f_m)$), and so epistasis cannot change the direction of selection on the drive allele. In contrast, we see that the LD dependent part of the change in the frequency of the modifier allele (eqn. S10) is always in the same direction of selection as the LD independent part, and hence accelerates the rate of evolution. Therefore, as confirmed by numerical simulation using the maximum value of negative LD, the sign of the change in modifier allele frequency (Δf_M) is always positive.

Overall, for post-meiotic killers with partial suppressors, we see that, while LD can lead to complicated pattern of accelerating or decelerating the rate of evolution of the drive and modifier alleles (see Table SA.3 for a summary), it never changes the direction of selection on the drive or modifier loci.

Table SA.3. Results from numerical analysis of different expressions from 10^7 randomly sampled sets of model parameters. The 'Sign of Δf_D or Δf_M (when $L = 0$)' refers to the sign of the change in allele frequency at the driver and modifier locus respectively in the absence of LD (*i.e.* is not multiplied by L), the 'Sign of Epistasis' refers to the sign of epistasis, and hence the sign of LD, created by selection (*i.e.* the sign of L), and the '% where LD part of $\Delta f_D > 0$' (or Δf_M) refers to the percentage of randomly sampled sets of parameter values where there is a positive sign for the part of the expression for the change in allele frequencies that depends on LD (*i.e.* the whole expression that is multiplied by L). The 'Impact on Driver Evolution' (or Modifier) summarises how LD impacts the rate of change in the frequency of the drive (or modifier) allele. For example, the second row corresponds to the numerical analysis of the pre-meiotic killer with a modifier showing either a full or partial suppression (since the outcome is the same) for the conditions where the drive allele and modifier allele are both favoured by selection (*i.e.* $\Delta f_D > 0$ and $\Delta f_M > 0$). This row shows that, in one-third of the randomly sampled sets of parameters, the LD dependent part of the change in the drive allele frequency is positive and hence accelerates the rate of evolution, while in none of the cases is the LD dependent part of the change in the modifier allele frequency positive, meaning that, for all sampled parameter values, LD decelerates the rate of evolution at the modifier locus.

**Killer Type**	**Modifier Type**	**Sign of Δf_D***	**Sign of Δf_M ***	**Sign of Epistasis**	**% where LD part of Δf_D>0**	**% where LD part of $\Delta f_M > 0$**	**Impact on Driver Evolution**	**Impact on Modifier Evolution**
Pre-meiotic	Full/Partial	−	+	+	100%	100%	Decelerates	Accelerates
Pre-meiotic	Full/Partial	+	+	−	33.3%	0%	Often decelerates[1]	Decelerates
Meso-meiotic	Full	−	+	+	100%	0%	Decelerates	Decelerates
Meso-meiotic	Full	+	−	−	0.6%	83.5%	Very often decelerates[1]	Often decelerates[2]
Meso-meiotic	Partial	−	+	+	100%	4.8%	Decelerates	Very often decelerates[3]
Meso-meiotic	Partial	+	−	−	1.6%	97.4%	Very often decelerates[1]	Very often decelerates[4]
Meso-meiotic	Partial	+	+	+	100%	12.6%	Accelerates	Very often decelerates[1]
Post-meiotic	Full	−	−	−	0%	100%	Accelerates	Decelerates
Post-meiotic	Full	+	−	−	0.8%	94.3%	Very often decelerates[1]	Very often decelerates[5]
Post-meiotic	Full	−	+	+	100%	0%	Decelerates	Decelerates
Post-meiotic	Full	−	+	−	0%	100%	Accelerates	Accelerates
Post-meiotic	Full	+	+	−	0%	100%	Decelerates	Accelerates
Post-meiotic	Partial	−	+	+	100%	0%	Decelerates	Decelerates
Post-meiotic	Partial	+	+	+	100%	0%	Accelerates	Decelerates
Post-meiotic	Partial	+	+	−	41.6%	100%	Often decelerates[1]	Accelerates

***** when L = 0

[1] Else 'Accelerates', which only occurs when $f_D < 0.5$.

[2] Else ‘Accelerates’, which only occurs when $f_M > 0.8$.

[3] Else ‘Accelerates’, which only occurs when $f_M < 0.15$.

[5] Else ‘Accelerates’, which only occurs when $f_M < 0.5$ or $f_M > 0.8$.

[6] Else ‘Accelerates’, which only occurs when $f_M > 0.85$.

Summary of results in support of the main text

The key finding of our analysis is that LD never changes the direction of selection across any of scenarios, which is confirmed by numerical simulation using the maximum value of LD. Across different scenarios with different meiotic drivers and suppressor modifiers, the impact of LD tends to decelerate the rate of evolution at the drive and modifier loci (see Table SA.4), which is particularly interesting when the drive or modifier alleles are favoured by selection. When the drive allele is favoured by selection, there tends to be negative epistasis that builds negative LD that accelerates the rate of change in the frequency of the drive allele when it is rarer and (more often) decelerates it when it is more common. This outcome arises across scenarios as a result of the increased frequency of the *Dm* haplotype, which happens because this is the allele combination that is the ‘active’ driver; LD creates an elevated frequency of interactions where it can gain a transmission advantage against other haplotypes when it is rare and an elevated frequency of interactions with itself where is suffers a toxicity effect when it is common. When the modifier allele is favoured by selection, there tends to be positive epistasis that builds positive LD that decelerates the rate of change in the frequency of the modifier allele by increasing the frequency of the *DM* haplotype (across scenarios) because this is the allele combination of the ‘inactive’ driver where the modifier takes its effect and can reap the benefit of removing the driver’s toxicity effect (although, sometimes, it also suffers a modified driver’s toxicity effect – and sometimes it also removes an asymmetry in the transmission advantage). When both the drive and the modifier allele are favoured, epistasis tends to be negative because the drive allele tends to have the overriding effect, as the drive allele is under stronger selection than the modifier allele due to having a transmission advantage against the nondrive allele, whereas the modifier gains against the nonmodifier allele from removing a toxicity effect.

Table SA.4. A summary of how LD impacts the rate of evolution of the drive (D) and modifier (M) system. The presence of LD never changes the direction of selection (*i.e.* the evolutionary outcome) in any scenario, but can change whether or not the rate of change in allele frequency is accelerated (↑), decelerated (↓), more often accelerated (↗) or more often decelerated (↘). Cells containing a '●' indicate scenarios that do not occur (*i.e.* the driver and modifier are never both simultaneously disfavoured for the pre-meiotic case).

**Scenario**	Driver & modifier both disfavoured [$\Delta f_D < 0; \Delta f_M < 0$]		Driver disfavoured; modifier favoured [$\Delta f_D < 0; \Delta f_M > 0$]		Driver favoured; modifier disfavoured [$\Delta f_D > 0; \Delta f_M < 0$]		Driver & modifier both favoured [$\Delta f_D > 0; \Delta f_M > 0$]	
	D	M	D	M	D	M	D	M
Pre-meiotic; Full/Partial suppression	●	●	↓	↑	●	●	↘	↓
Meso-meiotic; Full suppression	●	●	↓	↓	↘	↘	●	●
Meso-meiotic; Partial suppression	●	●	↓	↘	↘	↘	↑	↘
Post-meiotic; Full suppression	↑	↓	↘	↘	↘	↘	↓	↑
Post-meiotic; Partial suppression	●	●	↓	↓	●	●	↗	↗

The role of restricted recombination

Recombination can potentially alter the pattern of selection on a meiotic driver and any modifiers because it can dictate the composition of the gamete pool produced by double heterozygotes. In particular, it can create a difference between the gamete pools produced by the genotype composed from the *DM* and *dm* haplotypes compared to that composed from the *dM* and *Dm* haplotypes, thereby altering how these two genotypes contribute to selection on the alleles. For example, individuals composed of the *DM* and *dm* haplotypes would produce primarily *DM* and *dm* gametes when the recombination rate is low, but would produce equal numbers of the *DM*, *dM*, *Dm*, and *dm* gametes with free recombination. This influence of recombination is independent of the role that recombination plays in modulating the level of linkage disequilibrium (LD) in a population. That is, in most population genetic models, recombination matters because it erodes LD, so its entire influence is via its effect on LD. We examine the influence of LD in the separate analysis above, and so focus here specifically on how recombination influences selection through its effect on the composition of the gamete pool.

The only scenario where restricted recombination actually alters the pattern of selection on gametes is the case of post-meiotic killing with modifiers showing full suppression. This is because this scenario is the only one where an asymmetry in the fitness of gametes allows for a change in selection as the composition of the gamete pool changes. Here recombination dictates the frequency of the poison producing *Dm* genotype within the gamete pool, with limited recombination reducing its frequency in the *DM*/*dm* genotype and increasing its frequency in the *dM*/*Dm* genotype. This difference in the gamete pools produced by the reciprocal double heterozygotes alters the pattern of selection in the post-meiotic case with a fully suppressing modifier because it has asymmetrical effects on selection in the two gamete pools. Although recombination causes this same difference in the frequency of the poison producing *Dm* genotype in the gamete pools in other scenarios, it has a symmetrical effect across the reciprocal double heterozygotes that does not lead to a change in the pattern of selection.

To model the influence of recombination we formulate a model that approaches the free recombination scenario as the rate of recombination (r) approaches a half and approaches the logical

alternative scenario as r approaches zero. The case when r is a half is explored in the main text and in the analysis of LD above. For the gametes produced by the *DM*/*dm* (‘coupling’ type), as r approaches zero the frequency of the poison producing *dM* genotype approaches zero, and the fitness of the gametes approach 1 (while the frequency of the *dM* and *Dm* gametes go to zero). For the gametes produced by the *dM*/*Dm* (‘repulsion’ type), as r approaches zero the frequency of the poison producing *dM* genotype approaches a half, and therefore the fitness model approaches that of the other cases in which the *Dm* genotype is present, but where there are only two gamete types produced (*e.g.* the *DM*/*Dm* genotype). These fitness values are given in Table SA.5. The case when r is zero is also implicitly explored is the main text, where the two-locus two-allele system with perfect physical linkage correspond to the one-locus three-allele system with the drive, nondrive, and resistant alleles because, phenotypically, the *Dm* haplotype is the same as the *D* allele, the *dM* and *dm* haplotypes are the same as the *d* allele and the *DM* haplotype is the same as the δ allele. Further, the three-allele treatment of the evolution of this system is preferable to considering the properties of multiple loci because the meiotic driver is only favoured by selection as part of the *Dm* haplotype (whilst the *DM* haplotype is not a meiotic driver) so the change in the drive allele (*D*) frequency does not really capture the evolutionary outcome on phenotypes. Therefore, the main text already describes the extreme cases of free recombination and no recombination: the evolutionary outcome of free recombination is the disfavouring of the modifier allele *M* (see Table 7), whilst the evolutionary outcome of no recombination is the unconditional favouring of the resistant allele δ (or the *DM* haplotype; see Table SA.5B).

The remaining question is, therefore, what effect an intermediate level of recombination ($0 < r < ½$) has on evolution in this scenario. To understand the influence of an intermediate level of recombination, we rewrite the expressions for the evolution of the drive and modifier allele frequencies to include r. We separate the expression into a first part that corresponds to the case where $r = 0$ and a second component that accounts for the influence of recombination. These expressions are equal to those in the main text for the case where $r = ½$ (*i.e.* when there is free recombination).

$$\Delta f_D \bar{w} = f_D f_d f_m \begin{bmatrix} e(1 - f_d f_M)(1 - hs) - s\big(h + f_D f_m(1 - 2h)\big) \\ +2r f_M\big(hs(f_D - f_d) + e(1 + f_d(1 - hs) - 2hrs)\big) \end{bmatrix} \tag{S12a}$$

$$\Delta f_M \bar{w} = f_D f_M f_m \begin{bmatrix} e(f_d f_M - 1)(1 - hs) + s(f_D(f_m - 2h f_m) + h) \\ +2r f_d\big(hs(f_M(1 + e) - f_m + 2er) - e(1 + f_M)\big) \end{bmatrix} \tag{S12b}$$

These expressions illustrate the potential for recombination to change the direction of selection at some threshold. Using numerical simulations in the same manner as for the analysis of LD, the direction of the change in allele frequency with no versus free recombination is the same for 81.8% of randomly sampled sets of parameters for the drive or modifier allele (though the estimates for the drive and modifier alleles differ by a small amount). In cases where parameter combinations lead to the direction of the change in allele frequency differing between the case of no versus free recombination for the drive or modifier allele, if we also randomly sample across a uniform distribution of recombination rates ($0 < r < ½$), we find that this variable intermediate level of recombination leads to the same direction of selection as the case of free recombination in 64.6% of randomly sampled sets of parameters (though, again, the estimates for the drive and modifier alleles slightly differ). Unsurprisingly, across the range of the recombination rate ($0 < r < ½$) for the drive and modifier allele, we tend to find that the direction of selection is more likely to be in the opposite direction to the case of free recombination as the recombination rate draws closer to zero ($r \to 0$) following a negative exponential-like distribution. So, the deviation from the case of free recombination mostly arises when the recombination rate is very low.

Following equations (S12a and S12b), the invasion criteria for the drive and modifier alleles are:

$$invD = f_m \begin{bmatrix} f_m e(1 - hs) - hs \\ +2rf_M\big(2e - hs(1 + e + 2er)\big) \end{bmatrix} \tag{S13a}$$

$$invM = f_D \begin{bmatrix} e(hs - 1) + s\big(h + f_D(1 - 2h)\big) \\ +2rf_d(e(2rhs - 1) - hs) \end{bmatrix} \tag{S13b}$$

A similar analysis can also be conducted using numerical simulations to assess the impact of an intermediate rate of recombination on invasion. The outcome (*i.e.* the sign of invasion criterion) with no versus free recombination is the same for 80.3% of randomly sampled sets of parameters for the drive allele and 13.7% of randomly sampled sets of parameters for the modifier allele. Hence, the advantage to the drive allele is not very sensitive to the presence of recombination, whereas the selective advantage to the modifier is removed by a lack of recombination in most cases. For parameter combinations where the sign of the invasion criteria differs between the cases of no versus free recombination for the drive or modifier allele, intermediate levels of recombination ($0 < r <$ ½) has the same sign as the free recombination scenario in 72.6% of randomly sampled sets of parameters for the drive allele and 87.4% of randomly sampled sets of parameters for the modifier allele. Therefore, we see that the presence of some intermediate level of recombination usually generates the same results as we see for the free recombination case. In this regard, the analysis of invasion criteria shows this pattern more clearly than the same analysis on the change in allele frequencies. In-keeping with this, across the range of the recombination rate ($0 < r <$ ½) for the drive and modifier allele, we tend to find that the direction of selection is more likely to be in the opposite direction to the case of free recombination as the recombination rate approaches zero ($r \rightarrow 0$) following a negative exponential-like distribution. This is particularly notable for the modifier allele, which shows evolutionary properties that tend to differ from the free recombination case if there is absolutely no recombination (since the system collapses to effectively a single-locus system, see Table SA.5B), but mostly follows the outcome for the free recombination scenario when some level of recombination is present. This makes sense given that the no recombination extreme removes the opportunity for certain sets of interactions that

are generated by recombination in the double heterozygotes (compare Tables SA.5A and SA.5B). Therefore, overall, these results support our discussion in the main text that focuses on the more general case of free recombination, given that most scenarios with restricted recombination tend to follow the evolutionary outcome given by that scenario.

Table SA.5. Fitness of gametes produced by the different two-locus genotypes under post-meiotic killing, where the drive allele, D, produces the poison in the daughter cells (*i.e.* the gametes) and the modifier allele, M, causes full suppression of the drive locus by blocking the production of the poison and the antidote. In this scenario, the rate of recombination, r, determines the frequency of gametes produced by the double heterozygote genotypes, with restricted recombination (r < ½) leading to a difference in the frequencies of gametes produced by the alterative haplotypic combinations for the double heterozygote (DM/dm versus Dm/dM). The 'Genotype' column lists the ten possible unordered genotypes as a function of their component haplotypes. The 'gametes' column refers to the alleles within interacting cell types. The 'Fitness' column gives the relative production of the different gametes contributed to the next generation by that genotype. **A)** shows the general pattern, where fitness depends on the rate of recombination for some cells (since the rate of recombination influences the frequency of the genotype within the gamete pool. The fitness pattern is equal to that in Table 5A when r = ½. **B)** the pattern of fitness for the case of complete physical linkage, where r = 0. The '—' indicates genotypes that do not exist when there is no recombination (since recombination is required to generate them).

A)

**Genotype**	**Gametes**	**Fitness**
DM/DM	DM	1
DM/Dm	DM	$(1-e)(1-hs)$
	Dm	$(1+e)(1-hs)$
Dm/Dm	Dm	$1-s$
DM/dM	DM	1
	dM	1
DM/dm	DM	$(1-2re)(1-2rhs)$
	Dm	$(1+6re)(1-2rhs)$
	dM	$(1-2re)(1-2rhs)$
	dm	$(1-2re)(1-2rhs)$
Dm/dM	DM	$(1-e)(1-hs)$
	Dm	$(1+(1+4r)e)(1-hs)$
	dM	$(1-e)(1-hs)$
	dm	$(1-e)(1-hs)$
Dm/dm	Dm	$(1+e)(1-hs)$
	dm	$(1-e)(1-hs)$
dM/dM	dM	1
dM/dm	dM	1
	dm	1
dm/dm	dm	1

B)

Genotype	Gametes	Fitness
DM/DM	DM	1
DM/Dm	DM	$(1-e)(1-hs)$
	Dm	$(1+e)(1-hs)$
Dm/Dm	Dm	$1-s$
DM/dM	DM	1
	dM	1
DM/dm	DM	1
	Dm	—
	dM	—
	dm	1
Dm/dM	DM	—
	Dm	$(1+e)(1-hs)$
	dM	$(1-e)(1-hs)$
	dm	—
Dm/dm	Dm	$(1+e)(1-hs)$
	dm	$(1-e)(1-hs)$
dM/dM	dM	1
dM/dm	dM	1
	dm	1
dm/dm	dm	1

The role of crossovers

A crossover occurs when genetic material is exchanged between non-sister chromatids of two homologous chromosomes in between the prophase and metaphase of meiosis I, which occurs before the independent assortment of homologous chromosomes in the anaphase of meiosis I. For pre-meiotic killing among parent (or somatic) cells, a crossover would not alter the genetic structure of the interaction because (regardless of whether killing takes place before or after crossover) all of the alleles remain together in the cell but in a different combination across homologous chromosomes, and so any drive allele remains susceptible to suppression. For post-meiotic killing among daughter (or gamete) cells, a crossover could alter the genetic structure of the interaction by mixing-up drive and modifier allele combinations as an aspect of recombination, which has already been explored in the preceding analysis (and in the main text). Lastly, for meso-meiotic killing among sister cells, a crossover would alter the genetic structure of the interaction by modifying the combinations of drive and modifier alleles present in sister cells, which has not yet been explored.

Meiosis takes place with an initial doubling of the genetic material to become tetraploid (*i.e.* four alleles per locus) for parent cells in prophase I before homologous chromosomes separate into sister cells in anaphase I to have two alleles per locus and sister chromatids separate into daughter cells in anaphase II to have one allele per locus. Crossovers may occur multiple times along a chromosome, but it may only lead to the transfer of one (of either) or both drive and modifier alleles, which affords a restricted range of effects by generating new combinations of alleles in a pair of sister cells. To model the role of crossovers, the probability of different allele transfers (and combinations of allele transfers) would need to be mapped to howsoever many crossover events, which has been explored elsewhere (Haig, 2010). Because we are interest in general properties of drive systems, we do not derive a detailed analysis since it would require a model for the distributions of crossovers. Rather, we develop a qualitative analysis that evaluates the general impact that crossing-over has on selection within the framework of our existing model. Because the impact of crossovers is quite straightforward, this approach is sufficient to capture the consequences of crossovers and is consistent with what has been suggested by other studies.

Crossovers only impact the genetic structure of sister cell interactions when at least one locus is heterozygous because this gives the possibility of exchanging a non-identical allele. There are, therefore, five different genotypes where crossing-over could play a role. However, irrespective of full or partial suppression, in two cases ($DdMM$, $ddMm$), the combinations of alleles in these genotypes do not lead to the expression of the poison among sister cells in either the presence or absence of crossing-over and so these can be ignored. In individuals with $DDMm$ and $Ddmm$ genotypes, crossing-over makes sister cells genetically identical, which disrupts the meso-meiotic killing by preventing one cell from gaining a transmission advantage over the other. In the case of $DDMm$, the transfer of the modifier allele leads to full or partial suppression of the drive allele, which eliminates the expression of the poison. This would shift the balance of selection toward favouring the drive allele through eliminating the toxicity effect would shift the balance of selection toward favouring the modifier allele (M), which is no longer subject to a transmission disadvantage or the toxicity effect. For $Ddmm$, the transfer of the drive allele leads to both sister cells containing the *Ddmm* configuration and thereby expressing the poison. Because they would be genetically identical, there can be no transmission bias for any allele, while all cells would suffer a toxicity effect. This would shift the balance of selection toward disfavouring the drive and nonmodifier alleles. In individuals with $DdMm$ genotypes, there are two possible combinations of alleles among homologous chromosomes, but crossing-over leads to a similar distribution of outcomes either way. In the absence of crossing-over there could be an interaction among sister cells with $DDMM$ and $ddmm$ genotypes where the poison is not expressed – and crossing-over cannot change this outcome. This has no effect on selection on either allele. Alternatively, in the absence of crossing-over there could be an interaction among sister cells with $DDmm$ and $ddMM$ genotypes where the poison is expressed by one sister cell. The transfer of the drive allele does not change the expression of the poison, but it does dilute the transmission advantage to the drive allele with a modifier for full suppression (because crossing-over means that one of the surviving daughter cells at the end of meiosis contains the nondrive allele rather than both containing the drive allele) and prevent a transmission advantage to the drive allele with a modifier for partial suppression. This would shift the balance of selection toward disfavouring the drive allele and favouring the modifier allele. The transfer

of the modifier allele (with or without the drive allele) prevents the expression of the poison in both sister cells, which removes any transmission and toxicity effects. This would shift the balance of selection toward disfavouring the drive allele and favouring the modifier allele.

Therefore, although this qualitative analysis does not indicate the exact point at which the presence of crossing-over would change the direction of selection on a drive allele or a modifier allele with full or partial suppression in the meso-meiotic killing scenario, there is a clear directional trend. Where crossing-over changes the interaction between sister cells, it tends to alter the transmission and/or toxicity effects to decrease the likelihood that the drive allele is favoured by selection and increase the likelihood that the modifier allele is favoured by selection. This trend is always the case when the drive locus is heterozygous, which represents the scenario for the invasion of the drive allele (see Table SA.6). Linking this result to the findings in the main text where a partially suppressing modifier may only invade a drive allele for meso-meiotic killing under specific conditions, crossing-over makes a meso-meiotic killer more vulnerable to suppression. As a result, a meso-meiotic killer is more evolutionarily robust to the threat of suppression when crossing-over is rare or does not occur at all, which would predict that a more persistent meiotic driver would be closer to (and so segregate with) the centromere or in an inversion. This is an important prediction about the properties of an evolutionarily robust meiotic driver that is well-known from many models and systems of meiotic drive (Pardo-Manuel De Villena & Sapienza, 2001; Malik & Henikoff, 2002; Burt & Trivers, 2008; Haig, 2010). Therefore, although the analysis of crossing-over is qualitative, the model identifies the same basic properties that have been found elsewhere: a driver is more open to invasion and more robust against suppression from a modifier if it segregates more closely with the centromere.

Table SA.6. Fitness of gametes produced by each of the two-locus genotypes under meso-meiotic killing with/without one crossover, where the drive allele produces the poison in the sister cells (for either full or partial suppression). The ‘Parent cell’ column lists the nine possible unordered genotypes as the product of two haplotypes, whilst the ‘Sister cell’ column refers to the alleles within interacting cell types. The ‘Fitness’ column gives the production of the different gametes contributed to the next generation by that genotype. Some rows have red text, which denotes a scenario that is repeated with a crossover event, where genetic material is exchanged between non-sister chromatids on homologous chromosomes. There are two loci (drive and modifier) that each have two alleles: the drive locus has a drive allele D, which encodes a poison and antidote, and nondrive allele d, while the modifier locus has a modifier allele M, which blocks the production of both the poison and antidote, and a nonmodifier allele m. A crossover event can transfer the drive allele only, the modifier allele only or the drive and modifier alleles together. Within an interaction, when the poison is produced it leads to a toxicity effect (s) to all gametes and a transmission advantage ($+e$) for those gametes that produce the antidote, alongside a corresponding transmission disadvantage ($-e$) for those gametes that do not produce the antidote. Shading is used to separate the sets of gametes produced by each individual’s diploid genotype (including any crossovers). The fitness pattern is comparable to that in Table 6. **A)** The modifier allele M causes full suppression of the drive locus by blocking the production of the poison and the antidote. **B)** The modifier allele M causes partial suppression of the drive locus by blocking the production of the poison but not the antidote.

A)

Parent cell	Sister cell	Fitness
$DMxDM$	$DDMM$	1
$DMxDm$	$DDMM$	$(1-e)(1-hs)$
	$DDmm$	$(1+e)(1-hs)$
$DMxDm$ + crossover	$DDMm$ vs $(DDMm)$	1
$DmxDm$	$DDmm$	$1-s$
$DMxdM$	$DDMM$	1
	$ddMM$	1
$DMxdM$ + crossover	$DdMM$ vs $(DdMM)$	1
$DMxdm$ or $DmxdM$	$DDMM$	1
	$DDmm$	$(1+e)(1-hs)$
	$ddMM$	$(1-e)(1-hs)$
	$ddmm$	1
$DMxdm$ + crossover	$DdMM$ (vs $Ddmm$)	$(1-e)(1-hs)$
	$Ddmm$ (vs $DdMM$)	$(1+e)(1-hs)$
	$DDMm$ (vs $ddMm$)	1
	$ddMm$ (vs $DDMm$)	1
	$DdMm$ (vs $DdMm$)	1
$DmxdM$ + crossover	$DdMM$ (vs $Ddmm$)	$(1-e)(1-hs)$
	$Ddmm$ (vs $DdMM$)	$(1+e)(1-hs)$
	$DDMm$ (vs $ddMm$)	1
	$ddMm$ (vs $DDMm$)	1
	$DdMm$ (vs $DdMm$)	1
$Dmxdm$	$DDmm$	$(1+e)(1-hs)$
	$ddmm$	$(1-e)(1-hs)$
$Dmxdm$ + crossover	$Ddmm$ vs $(Ddmm)$	$1-s$
$dMxdM$	$ddMM$	1
$dMxdm$	$ddMM$	1
	$ddmm$	1
$dMxdm$ + crossover	$ddMm$ vs $(ddMm)$	1
$dmxdm$	$ddmm$	1

B)

Parent cell	Sister cell	Fitness
$DMxDM$	$DDMM$	1
$DMxDm$	$DDMM$	$1-hs$
	$DDmm$	$1-hs$
$DMxDm$ + crossover	$DDMm$ vs $(DDMm)$	1
$DmxDm$	$DDmm$	$1-s$
$DMxdM$	$DDMM$	1
	$ddMM$	1
$DMxdM$ + crossover	$DdMM$ vs $(DdMM)$	1
$DMxdm$ or $DmxdM$	$DDMM$	1
	$DDmm$	$(1+e)(1-hs)$
	$ddMM$	$(1-e)(1-hs)$
	$ddmm$	1
$DMxdm$ + crossover	$DdMM$ (vs $Ddmm$)	$1-hs$
	$Ddmm$ (vs $DdMM$)	$1-hs$
	$DDMm$ (vs $ddMm$)	1
	$ddMm$ (vs $DDMm$)	1
	$DdMm$ (vs $DdMm$)	1
$DmxdM$ + crossover	$DdMM$ (vs $Ddmm$)	$1-hs$
	$Ddmm$ (vs $DdMM$)	$1-hs$
	$DDMm$ (vs $ddMm$)	1
	$ddMm$ (vs $DDMm$)	1
	$DdMm$ (vs $DdMm$)	1
$Dmxdm$	$DDmm$	$(1+e)(1-hs)$
	$ddmm$	$(1-e)(1-hs)$
$Dmxdm$ + crossover	$Ddmm$ vs $(Ddmm)$	$1-s$
$dMxdM$	$ddMM$	1
$dMxdm$	$ddMM$	1
	$ddmm$	1
$dMxdm$ + crossover	$ddMm$ vs $(ddMm)$	1
$dmxdm$	$ddmm$	1

References

Burt, A. & Trivers, R. 2008. Genes in Conflict. Harvard University Press.

Haig, D. 2010. Games in Tetrads: Segregation, Recombination, and Meiotic Drive. Am. Nat. 176: 404–413.

Hastings, A. 1985. Multilocus population genetics with weak epistasis. I. Equilibrium properties of two-locus two-allele models. Genetics 109: 799–812.

Lewontin, R.C. 1974. The genetic basis of evolutionary change. Columbia University Press.

Malik, H.S. & Henikoff, S. 2002. Conflict begets complexity: The evolution of centromeres. Curr. Opin. Genet. Dev. 12: 711–718.

Nagylaki, T. 1993. The evolution of multilocus systems under weak selection. Genetics 134: 627–647.

Pardo-Manuel De Villena, F. & Sapienza, C. 2001. Nonrandom segregation during meiosis: The unfairness of females. Mamm. Genome 12: 331–339.
